# Synthesis and Antitumor Activity of 1-Substituted 1,2,3-Triazole-Mollugin Derivatives

**DOI:** 10.3390/molecules26113249

**Published:** 2021-05-28

**Authors:** Han Luo, Yong-Feng Lv, Hong Zhang, Jiang-Miao Hu, Hong-Mei Li, Shou-Jin Liu

**Affiliations:** 1 College of Pharmacy, Anhui University of Chinese Medicine, Hefei 230011, China; luohan@stu.ahtcm.edu.cn (H.L.); zhanghong@ahtcm.edu.cn (H.Z.); 2 State Key Laboratory of Phytochemistry and Plant Resources in West China, Kunming Institute of Botany, Chinese Academy of Sciences, Kunming 650201, China; lvyongfeng@mail.kib.ac.cn (Y.-F.L.); hujiangmiao@mail.kib.ac.cn (J.-M.H.); lihongmei@mail.kib.ac.cn (H.-M.L.)

**Keywords:** mollugin, triazoles, antitumor activity, synthesis

## Abstract

A new series of mollugin-1,2,3-triazole derivatives were synthesized using a copper(I)-catalyzed Huisgen 1,3-dipolar cycloaddition reaction of corresponding *O*-propargylated mollugin with aryl azides. All the compounds were evaluated for their cytotoxicity on five human cancer cell lines (HL-60, A549, SMMC-7721, SW480, and MCF-7) using MTS assays. Among the synthesized series, most of them showed cytotoxicity and most of all, compounds **14** and **17** exhibited significant cytotoxicity of all five cancer cell lines.

## 1. Introduction

Mollugin, a methyl ester derivative of naphthoquinone extracted from the roots of Rubia Cordifolia [1,2], has been known have to broad spectrum of biological activities, including neuroprotective [3], anti-inflammatory [4,5], anti-bacterial [6], and antitumor activities [7,8,9,10,11]. In particular, mollugin displays indirect antitumor activity in various tumor models. For example:

(1) It can inhibit the secretion of hepatitis B surface antigen in human hepatocellular carcinoma Hep3B cells with IC_50_ = 2.0 μg/mL [7].
(2) It can adjust the signal pathways of HER2/Akt/SREBP-1c to block the fatty acid synthase (FAS) gene expression, thus inhibits the human epidermal growth factor receptor 2 (HER2) gene expression of cancer cell proliferation and induces its apoptosis [8].
(3) It induces tumor cell apoptosis and autophagy through the PI3K/Akt/mTOR/p7-0S6K and extracellular regulated protein kinases (ERK) signaling pathways [9].
(4) It also significantly inhibits the expression of the NF-κB reporter gene which is induced by TNF-α in a dose-dependent manner to restrain tumor cell proliferation [10,11].

Although mollugin has promising anticancer activity, it has little effect on the viability of cancer cells directly. Therefore, we tried to introduce new groups based on mollugin to enhance direct cytotoxicity of mollugin on cancer cells in the further investigation. Through literature research, we found that mollugin derivatives have been synthesized through modification of the ester group (C-2) and substitution reactions (C-4, C-6, C-7, C-1’ and C-2’) [12,13]. To our surprise, the hydroxyl group (C-1) of mollugin has not been modified and we synthesized mollugin derivatives by modifying this group.

1,2,3-Triazoles are attractive connecting units, as they are stable with metabolic degradation and capable of hydrogen bonding, which can be favorable in binding of biomolecular targets and solubility [14,15]. Therefore, 1,2,3-Triazole is often used as a functional group that needs to be considered in the process of drug design [16,17]. In addition, the click reaction of copper(I)-catalyzed Huisgen 1,3-dipolar cycloaddition has been widely used to covalently link two molecular fragments between a terminal alkyne and an azide to generate substituted 1,2,3-triazoles [18,19]. It is worth mentioning that the reaction was generally regiospecific in forming only the 1,4-substituted 1,2,3-triazole, which facilitates the further purification of the target product [20,21].

**Figure 1:**
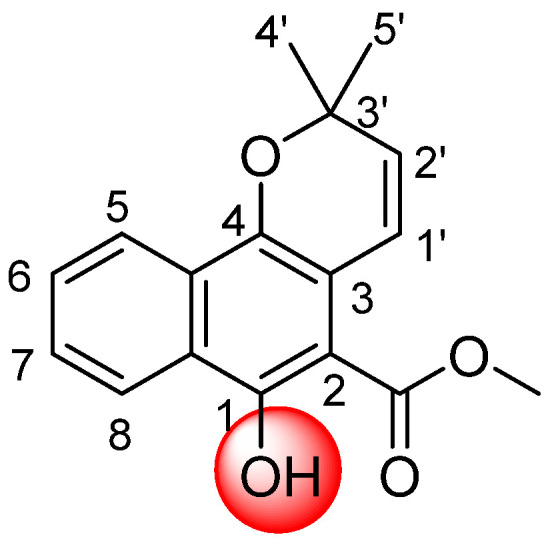
The structure of mollugin (**1**).

In this manuscript, the key intermediate was obtained by proparylation of the hydroxyl group (C-1) of mollugin (Figure 1). Then a series of mollugin derivatives were synthesized through the click chemistry approach by introducing different substituted aromatic azides [22,23,24]. Further the synthesized derivatives were screened for cytotoxicity against five different human cancer cell lines (HL-60, A549, SMMC-7721, SW480, and MCF-7).

## 2. Results and Discussion

### 2.1. Chemistry

**Scheme 1:**
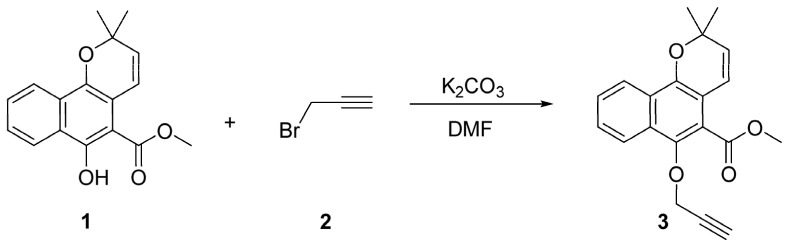
Preparation of *O*-propargylated mollugin (**3**).

The key intermediate (**3**) was obtained as shown in Scheme 1. In the presence of potassium carbonate, treatment of mollugin (**1**) with 3-bromoprop-1-yne (**2**) in anhydrous DMF yielded the *O*-propargylated mollugin (**3**) in 85% yield [25,26]. 

**Scheme 2:**
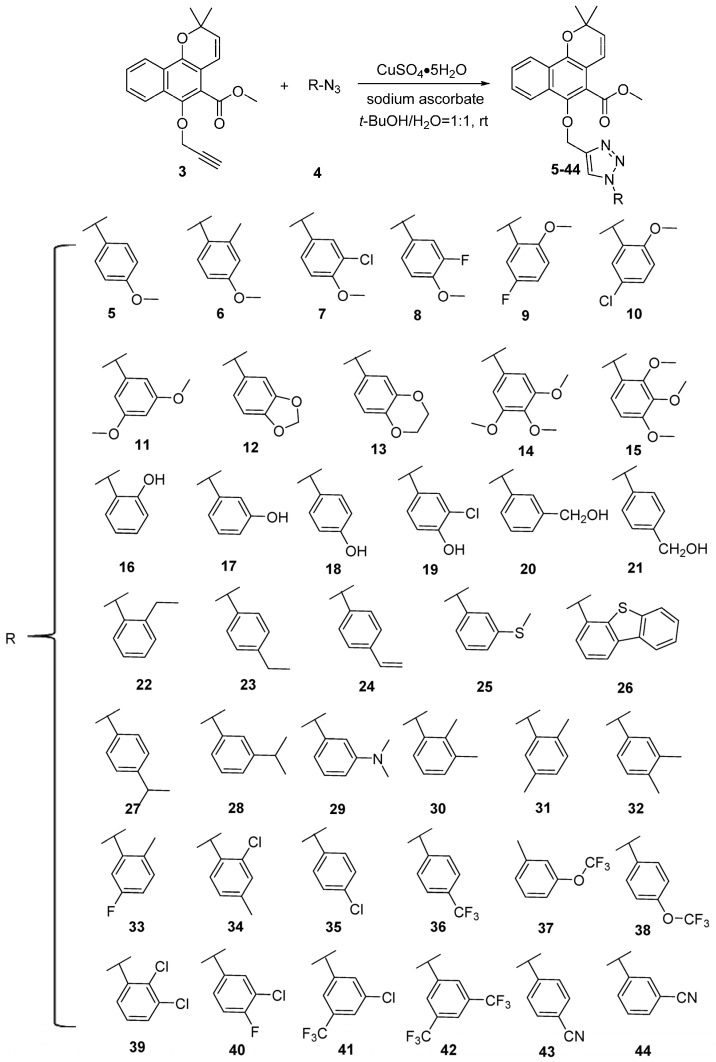
Preparation of 1-substituted 1,2,3-triazole-mollugin derivatives (**5–44**).

The 1-substituted 1,2,3-triazole-mollugin derivatives were synthesized using a copper(I)-catalyzed Huisgen 1,3-dipolar cycloaddition reaction of the corresponding *O*-propargylated mollugin (**3**) with different substituted aromatic azides (Scheme 2) [18,19]. In addition, all aromatic azides were prepared from corresponding boronic acid with sodium azide in the presence of CuSO_4_ in methanol (MeOH) without further purification [27,28].

As we can see from Scheme 2, 40 mollugin derivatives were obtained via the key click reaction. All the compounds present different substituents at the triazole moiety to evaluate their influence on the antitumor activity. Thus, mollugin derivatives with an aromatic ring with electron-donating groups or electron-withdrawing groups were prepared. All the synthesized triazolyl derivatives (**5–44**) were characterized by ^1^H NMR, ^13^C NMR, and HRMS spectroscopic study (see Supplementary Materials).

### 2.2. Evaluation of Biological Activity

Compound **1** and its synthesized derivatives were screened against a group of five different human cancer cell lines (HL-60, A549, SMMC-7721, SW480, and MCF-7) to evaluate their cytotoxic potential using MTS assay [29,30]. Cisplatin (DDP) and Taxol (TAX) were taken as reference drugs and their IC_50_ data were present in Table 1. More than half of the derivatives exhibited better cytotoxic activity than mollugin.

Some of derivatives displayed good cytotoxicity (IC_50_ < 20 μM) and even more potent than the control drug DDP, compounds **5**, **9**, **11**, **14**, **15**, **16**, **17**, **18**, **19**, **20**, **21** and **29** showed maximum inhibition effects against liver cancer cell line (SMMC-7721). Against the breast cancer cell line (MCF-7), compounds **11**, **14**, **16**, **17**, and **20** demonstrate cytotoxicity. Compounds **11**, **14**, **17**, **218** and **19** displayed maximum inhibition effects against leukemia cells (HL-60). Compounds **14**, **16**, **17** and **36** displayed maximum inhibition effects against lung cancer cells (A549) whereas compounds **14** and **17** sensitized colon cancer cells (SW480) the most. Overall, the cytotoxicity of the derivatives was generally stronger than the parent molecule, the SMMC-7721 cell line was most sensitive to these compounds and compounds **14** and **17** exhibited significant inhibition effects against all the experimental cancer cell lines. 

These data have allowed us to carry out a structure and activity relationship (SAR) study on the influence of the modifications of different group in the cytotoxicity. The main results can be summarized as follows: derivatives containing electron-donating groups such as hydroxyl, methoxy, and alcohol hydroxyl groups tend to have good cytotoxicity. By comparing IC_50_ value of compounds **5**, **11**, **14**, and 15, it could be concluded that cytotoxicity increased with the growth of methoxy group number in those derivatives. According to the experimental results, derivatives that contain electron-withdrawing groups do not have cytotoxicity except for compound **36**. Compound **36** possesses notable cytotoxicity against A549 cancer cells with IC_50_ value of 4.82 ± 0.84 μM, which is triple and quadruple improvement in cytotoxicity compared to the control drug DDP.

## 3. Materials and Methods

### 3.1. General Experimental Procedures

All the reagents and solvents used for purification and synthesis were purchased from Meryer. All synthesized derivatives were purified by column chromatography (silica gel, petroleum ether/ethyl acetate, 20:1 to 1:1 and petroleum ether/acetone, 20:1 to 1:1) and their structures were elucidated by ^1^H NMR, ^13^C NMR, high-resolution mass spectrometry (HR-ESIMS). Mass spectra were performed on UPLC-IT-TOF (Shimadzu, Kyoto, Japan) spectrometer. NMR spectra were recorded on AVANCE III 400 MHz (Bruker, Bremerhaven, Germany) and Avance III 600 MHz (Bruker, Bremerhaven, Germany) instruments using CDCl_3_, CD_3_OD or acetone-*d*_6_ as the solvent with TMS as the internal standard. Chemical shifts (*δ*) were reported in parts per million (ppm) and the coupling constants (*J*) were given in Hertz. Column chromatography was performed on silica gel (200–300 and 300–400 mesh, Qingdao Makall Group CO., Qingdao, China). All chemical reactions were monitored by TLC on silica gel 60 F254 plates and the spots were visualized by UV light and sprayed with 10% H_3_PO_4_·12MoO_3_ in EtOH, followed by heating. All compounds were named using the ACD40 Name-Pro program, which is based on IUPAC rules. Azides (**4**) were synthesized according to procedures previously described in the literature [27,28].

*prop-1-yne-O-mollugin* (**3**). To a solution of mollugin (1.00 g, 3.52 mmol, 1.0 eq) in DMF (15 mL) was added K_2_CO_3_ (725 mg, 5.28 mmol, 1.5 eq) slowly. The reaction mixture was stirred at rt for 15 min, and propargyl bromide (0.37 mL, 4.23 mmol, 1.2 eq) was added dropwise at rt. The reaction mixture was stirred at rt for 8 h before it was quenched by saturated NH_4_Cl aqueous solution (20 mL), and the mixture was extracted with ethyl acetate (3 × 20 mL). The combined organic layer was washed with brine (2 × 40 mL), and dried over Na_2_SO_4_, and filtered. After removal of the solvent under vacuum, the residue was purified by flash column chromatography on silica gel (12:1 to 8:1 petroleum ether/EtOAc) provided compound **3** (964 mg, 82% yield) as a yellow solid, R_f_ = 0.3 (petroleum ether/EtOAc = 10:1) [25].

### 3.2. General Procedures for the Preparation of 1-Substituted 1,2,3-Triazole-Mollugin Derivatives

To a solution of 0.2 mmol of the corresponding azide in 3 mL mixed solution (*t*-BuOH/H_2_O = 1:1, *v/v*) was added *O*-propargylated mollugin (0.2 mmol), sodium ascorbate (0.02 mmol), CuSO_4_·5H_2_O (0.02 mmol). The reaction mixture was stirred for 48 h at room temperature before it was quenched by saturated NH_4_Cl aqueous solution (4 mL), and the mixture was extracted with ethyl acetate (3 × 6 mL). The combined organic layer was washed with brine (2 × 15 mL), and dried over Na_2_SO_4_, and filtered [31,32]. After removal of the solvent under vacuum, the residue was purified by flash column chromatography on silica gel (10/1 to 2/1 petroleum ether/EtOAc) provided compound **5–44**. 

*1-O-((1-(4-methoxyphenyl)-1H-1,2,3-triazol)-4-yl)methyl)-mollugin (**5**)*. Yield: 89%, yellow oil, ^1^H NMR (CDCl_3_, 400 MHz) *δ* 8.22 (dd, 1H, *J* = 6.3, 3.3 Hz), 8.17 (dd, 1H, *J* = 6.3, 3.3 Hz), 8.01 (s, 1H), 7.64 (d, 2H, *J* = 8.9 Hz), 7.52 (m, 2H), 7.02 (d, 2H, *J* = 8.9 Hz), 6.44 (d, 1H, *J* = 9.9 Hz), 5.70 (d, 1H, *J* = 9.9 Hz), 5.32 (s, 2H), 3.94 (s, 3H), 3.86 (s, 3H), 1.52 (s, 6H); ^13^C NMR (CDCl_3_, 100 MHz) *δ* 167.6, 159.9, 145.9, 145.4, 144.7, 130.5, 130.3, 127.9, 127.2, 127.1, 126.8, 122.8, 122.5, 122.4, 76.6, 69.1, 55.7, 52.5, 27.7; ESIMS: *m/z* 494 [M+Na]^+^, HRESIMS: calcd for C_27_H_25_N_3_O_5_Na [M+Na]^+^ 494.1688, found 494.1686.

*1-O-((1-(4-methoxy-2-methylphenyl)-1H-1,2,3-triazol)-4-yl)methyl)-mollugin (**6**)*. Yield: 70%, yellow solid, MP: 157–159 °C, ^1^H NMR (CDCl_3_, 400 MHz) *δ* 8.22 (dd, 1H, *J* = 6.4, 3.3 Hz), 8.15 (dd, 1H, *J* = 6.5, 3.2 Hz), 7.72 (s, 1H), 7.51 (m, 2H), 7.23 (d, 1H, *J* = 8.5 Hz), 6.84 (s, 1H), 6.82 (d, 1H, *J* = 8.5 Hz), 6.44 (d, 1H, *J* = 10.0 Hz), 5.85 (d, 1H, *J* = 10.0 Hz), 5.34 (s, 2H), 3.95 (s, 3H), 3.84 (s, 3H), 2.12 (s, 3H), 1.52 (s, 6H); ^13^C NMR (CDCl_3_, 100 MHz) *δ* 167.6, 160.4, 145.7, 145.3, 143.6, 135.4, 130.3, 129.6, 128.1, 127.3, 127.1, 127.0, 126.7, 125.3, 122.9, 122.4, 121.2, 119.9, 116.3, 112.4, 111.8, 76.6, 68.9, 55.6, 52.5, 27.7, 18.0; ESIMS: *m/z* 508 [M+Na]^+^, HRESIMS: calcd for C_28_H_27_N_3_O_5_Na [M+Na]^+^ 508.1840, found 508.1843.

*1-(3-chloro-4-methoxyphenyl)-4-ethyl-1H-1,2,3-triazole-O-mollugin (**7**)*. Yield: 72%, yellow solid, MP: 125–127 °C, ^1^H NMR (CDCl_3_, 400 MHz) *δ* 8.22 (dd, 1H, *J* = 6.4, 3.3 Hz), 8.14 (dd, 1H, *J* = 6.4, 3.3 Hz), 8.00 (s, 1H), 7.78 (d, 1H, *J* = 2.6 Hz), 7.60 (dd, 1H, *J* = 8.9, 2.6 Hz), 7.52 (m, 2H), 7.03 (d, 1H, *J* = 8.9 Hz), 6.44 (d, 1H, *J* = 10.0 Hz), 5.70 (d, 1H, *J* = 10.0 Hz), 5.31 (s, 2H), 3.96 (s, 3H), 3.94 (s, 3H), 1.52 (s, 6H); ^13^C NMR (CDCl_3_, 100 MHz) *δ* 167.6, 155.4, 145.7, 145.4, 144.9, 130.5, 130.3, 127.9, 127.2, 127.1, 126.8, 123.6, 123.1, 122.7, 122.5, 121.7, 121.1, 119.8, 112.4, 76.6, 69.0, 56.5, 52.5, 27.7; ESIMS: *m/z* 528 [M+Na]^+^, HRESIMS: calcd for C_27_H_23_N_3_O_5_ClNa [M+Na]^+^ 528.1296, found 528.1297.

*1-O-((1-(3-fluoro-4-methoxyphenyl)-1H-1,2,3-triazol)-4-yl)methyl)-mollugin (**8**).* Yield: 78%, yellow oil, ^1^H NMR (CDCl_3_, 400 MHz) *δ* 8.22 (dd, 1H, *J* = 6.5, 3.3 Hz), 8.14 (dd, 1H, *J* = 6.5, 3.2 Hz), 8.01 (s, 1H), 7.52 (m, 3H), 7.44 (m, 1H), 7.07 (t, 1H, *J* = 8.8 Hz), 6.44 (d, 1H, *J* = 9.9 Hz), 5.70 (d, 1H, *J* = 9.9 Hz), 5.32 (s, 2H), 3.95 (s, 3H), 3.94 (s, 3H), 1.52 (s, 6H); ^13^C NMR (CDCl_3_, 100 MHz) *δ* 167.6, 153.6, 151.1, 148.2, 145.7, 145.5, 144.9, 130.3, 127.9, 127.2, 127.1, 126.8, 122.7, 122.5, 121.7, 121.1, 119.8, 116.6, 113.8, 112.4, 109.9, 76.6, 69.0, 56.5, 52.5, 27.7; ESIMS: *m/z* 512 [M+Na]^+^, HRESIMS: calcd for C_27_H_24_N_3_O_5_FNa [M+Na]^+^ 512.1594, found 512.1592.

*1-O-((1-(5-fluoro-2-methoxyphenyl)-1H-1,2,3-triazol)-4-yl)methyl)-mollugin (**9**).* Yield: 48%, yellow oil, ^1^H NMR (CDCl_3_, 400 MHz) *δ* 8.26 (s, 1H), 8.22 (dd, 1H, *J* = 6.4, 3.3 Hz), 8.17 (dd, 1H, *J* = 6.4, 3.3 Hz), 7.65 (dd, 1H, *J* = 8.7, 3.1 Hz), 7.52 (m, 2H), 7.13 (m, 1H), 7.03 (m, 1H), 6.44 (d, 1H, *J* = 9.9 Hz), 5.69 (d, 1H, *J* = 9.9 Hz), 5.33 (s, 2H), 3.95 (s, 3H), 3.88 (s, 3H), 1.52 (s, 6H); ^13^C NMR (CDCl_3_, 100 MHz) *δ* 167.7, 157.8, 155.4, 147.1, 145.9, 145.4, 143.8, 130.3, 128.0, 127.1, 127.0, 126.8, 125.3, 122.9, 122.5, 121.1, 119.8, 116.2, 113.3, 112.7, 112.5, 76.6, 69.0, 56.6, 52.5, 27.7; ESIMS: *m/z* 512 [M+Na]^+^, HRESIMS: calcd for C_27_H_24_N_3_O_5_FNa [M+Na]^+^ 512.1590, found 512.1592.

*1-O-((1-(5-chloro-2-methoxyphenyl)-1H-1,2,3-triazol)-4-yl)methyl)-mollugin (**10**)*. Yield: 92%, yellow solid, MP: 159–161 °C, ^1^H NMR (CDCl_3_, 400 MHz) *δ* 8.24–8.20 (m, 2H), 8.17 (dd, 1H, *J* = 6.3, 3.4 Hz), 7.86 (d, 1H, *J* = 2.6 Hz), 7.52 (m, 2H), 7.38 (dd, 1H, *J* = 8.9 Hz, 2.6 Hz), 7.01 (d, 1H, *J* = 8.9Hz), 6.44 (d, 1H, *J* = 9.9 Hz), 5.70 (d, 1H, *J* = 9.9Hz), 5.33 (s, 2H), 3.95 (s, 3H), 3.89 (s, 3H), 1.52 (s, 6H); ^13^C NMR (CDCl_3_, 100 MHz) *δ* 167.7, 149.6, 145.9, 145.4, 143.8, 130.3, 129.7, 128.0, 127.1, 127.0, 126.9, 126.8, 126.3, 125.3, 122.9, 122.5, 121.1, 119.8, 113.4, 112.4, 76.6, 69.1, 56.4, 52.5, 27.7; ESIMS: *m/z* 528 [M+Na]^+^, HRESIMS: calcd for C_27_H_24_N_3_O_5_ClNa [M+Na]^+^ 528.1296, found 528.1297.

*1-O-((1-(3,5-dimethoxyphenyl) -1H-1,2,3-triazol)-4-yl)methyl)-mollugin(**11**)*. Yield: 84%, yellow solid, MP: 51–53 °C, ^1^H NMR (CDCl_3_, 400 MHz) *δ* 8.22 (dd, 1H, *J* = 6.4, 3.5 Hz), 8.16 (dd, 1H, *J* = 6.4, 3.5 Hz), 8.07 (s, 1H), 7.52 (m, 2H), 6.91 (d, 2H, *J* = 2.2 Hz), 6.51 (t, 1H, *J* = 2.3 Hz), 6.44 (d, 1H, *J* = 9.9Hz), 5.69 (d, 1H, *J* = 9.9 Hz), 5.32 (s, 2H), 3.94 (s, 3H), 3.85 (s, 6H), 1.52 (s, 6H); ^13^C NMR (CDCl_3_, 100 MHz) *δ* 167.6, 161.5, 145.8, 145.4, 144.8, 138.5, 130.3, 127.9, 127.2, 127.1, 126.8, 122.7, 122.5, 121.7, 121.1, 119.9, 112.4, 100.7, 99.1, 76.6, 69.1, 55.7, 52.5, 27.7; ESIMS: *m/z* 524 [M+Na]^+^, HRESIMS: calcd for C_28_H_27_N_3_O_6_Na [M+Na]^+^ 524.1793, found 524.1792.

*1-O-((1-(benzo[d][1,3]dioxol-5-yl)-1H-1,2,3-triazol)-4-yl)methyl)-mollugin (**12**)*. Yield: 72%, yellow solid, MP: 73–75 °C, ^1^H NMR (CDCl_3_, 400 MHz) *δ* 8.22 (dd, 1H, *J* = 6.5, 3.3 Hz), 8.15 (dd, 1H, *J* = 6.5, 3.3 Hz), 7.98 (s, 1H), 7.52 (m, 2H), 7.24 (d, 1H, *J* = 2.2 Hz), 7.14 (dd, 1H, *J* = 8.3, 2.2 Hz), 6.90 (d, 1H, *J* = 8.3 Hz), 6.44 (d, 1H, *J* = 9.9 Hz), 6.06 (s, 2H), 5.69 (d, 1H, *J* = 9.9 Hz), 5.31 (s, 2H), 3.94 (s, 3H), 1.52 (s, 6H); ^13^C NMR (CDCl_3_, 100 MHz) *δ* 167.6, 148.6, 148.1, 145.8, 145.4, 144.7, 131.5, 130.3, 127.9, 127.1, 127.1, 126.8, 122.8, 122.5, 121.9, 121.1, 119.9, 114.5, 112.4, 108.5, 103.0, 102.1, 76.6, 69.0, 52.5, 27.7; ESIMS: *m/z* 508 [M+Na]^+^, HRESIMS: calcd for C_27_H_23_N_3_O_6_Na [M+Na]^+^ 508.1474, found 508.1479.

*1-O-((1-(2,3-dihydrobenzo[b][1,4]dioxin-6-yl)-1H-1,2,3-triazol)-4-yl)methyl)-mollugin (**13**).* Yield: 82%, yellow solid, MP: 71–73 °C, ^1^H NMR (CDCl_3_, 400 MHz) *δ* 8.22 (dd, 1H, *J* = 6.4, 3.3 Hz), 8.16 (dd, 1H, *J* = 6.4, 3.2 Hz), 7.98 (s, 1H), 7.52 (m, 2H), 7.27 (d, 1H, *J* = 2.6 Hz), 7.17 (dd, 1H, *J* = 8.7, 2.6 Hz), 6.96 (d, 1H, *J* = 8.7Hz) 6.42 (d, 1H, *J* = 9.9Hz), 5.68 (d, 1H, *J* = 9.9 Hz), 4.29 (s, 4H), 3.92 (s, 3H), 1.52 (s, 6H); ^13^C NMR (CDCl_3_, 100 MHz) *δ* 167.6, 145.8, 145.3, 144.6, 144.1, 130.9, 130.3, 127.9, 127.1, 127.0, 126.8, 122.8, 122.5, 121.7, 121.1, 119.9, 118.1, 114.0, 112.4, 110.5, 76.6, 69.1, 64.4, 52.5, 27.7; ESIMS: *m/z* 522 [M+Na]^+^, HRESIMS: calcd for C_28_H_25_N_3_O_6_Na [M+Na]^+^ 522.1638, found 522.1636.

*1-O-((1-(3,4,5-trimethoxyphenyl)-1H-1,2,3-triazol)-4-yl)methyl)-mollugin(**14**).* Yield: 76%, yellow solid, MP: 55–57 °C, ^1^H NMR (CDCl_3_, 400 MHz) *δ* 8.22 (dd, 1H, *J* = 6.5, 3.3 Hz), 8.14 (dd, 1H, *J* = 6.4, 3.2 Hz), 8.03 (s, 1H), 7.52 (m, 2H), 6.95 (s, 2H) 6.44 (d, 1H, *J* = 9.9Hz), 5.70 (d, 1H, *J* = 9.9 Hz), 5.33 (s, 2H), 3.93 (s, 9H), 3.89 (s, 3H), 1.52 (s, 6H); ^13^C NMR (CDCl_3_, 100 MHz) *δ* 167.6, 153.9, 145.7, 145.5, 144.8, 138.4, 132.9, 130.3, 127.9, 127.2, 127.1, 126.8, 122.7, 122.5, 121.8, 121.3, 119.8, 112.4, 98.7, 76.6, 69.1, 61.1, 56.5, 52.5, 27.7; ESIMS: *m/z* 554 [M+Na]^+^, HRESIMS: calcd for C_29_H_29_N_3_O_7_Na [M+Na]^+^ 554.1899, found 554.1898.

*1-O-((1-(2,3,4-trimethoxyphenyl)-1H-1,2,3-triazol)-4-yl)methyl)-mollugin (**15**).* Yield: 47%, yellow solid, MP: 123–125 °C, ^1^H NMR (CDCl_3_, 400 MHz) *δ* 8.25–8.15 (m, 2H), 8.07 (s, 1H), 7.52 (m, 2H), 7.42 (d, 2H, 9.0 Hz), 6.79 (d, 1H, 9.0 Hz), 6.44 (d, 1H, *J* = 9.9 Hz), 5.69 (d, 1H, *J* = 9.9 Hz), 5.33 (s, 2H), 3.97 (s, 3H), 3.93 (s, 3H), 3.93 (s, 3H), 3.73 (s, 3H), 1.52 (s, 6H); ^13^C NMR (CDCl_3_, 100 MHz) *δ* 167.7, 154.4, 146.7, 145.9, 145.3, 143.7, 142.7, 130.2, 128.1, 127.1, 127.0, 126.8, 125.2, 124.5, 122.9, 122.4, 121.1, 120.1, 119.9, 112.4, 107.2, 76.5, 69.0, 61.6, 61.2, 56.2, 52.5, 27.7; ESIMS: *m/z* 554 [M+Na]^+^, HRESIMS: calcd for C_29_H_29_N_3_O_7_Na [M+Na]^+^ 554.1898, found 554.1898.

*1-O-((1-(2-hydroxyphenyl)-1H-1,2,3-triazol-4-yl)methyl)-O-mollugin (**16**).* Yield: 67%, white solid, MP: 183–185 °C, ^1^H NMR (CDCl_3_, 400 MHz) *δ* 9.59 (s, 1H), 8.24 (dd, 1H, *J* = 6.5, 3.1 Hz), 8.14 (m, 2H), 7.54 (m, 2H), 7.42 (dd, 1H, *J* = 8.1, 1.6 Hz), 7.32 (t, 1H, *J* = 7.1 Hz), 7.20 (dd, 1H, *J* = 8.3, 1.4 Hz), 6.42 (d, 1H, *J* = 9.9 Hz), 5.70 (d, 1H, *J* = 9.9 Hz), 5.35 (s, 2H), 3.93 (s, 3H), 1.53 (s, 6H); ^13^C NMR (CDCl_3_, 100 MHz) *δ* 167.7, 149.5, 145.6, 145.5, 144.2, 130.4, 130.0, 127.7, 127.2, 127.1, 126.9, 123.0, 122.6, 122.5, 121.9, 121.2, 120.4, 120.4, 119.8, 119.4, 112.3, 76.6, 68.6, 52.7, 27.7; ESIMS: *m/z* 456 [M−H]^−^, HRESIMS: calcd for C_26_H_23_N_3_O_5_Na [M−H]^−^ 456.1569, found 456.1565.

*1-O-((1-(3-hydroxyphenyl)-1H-1,2,3-triazol-4-yl)methyl)-mollugin (**17**).* Yield: 55%, white solid, MP: 182–184 °C, ^1^H NMR (CDCl_3_, 400 MHz) *δ* 9.44 (s, 1H), 8.45 (s, 1H), 8.24–8.13 (m, 3H), 7.54 (m, 2H), 7.36 (t, 1H, *J* = 8.1 Hz), 7.07 (dd, 1H, *J* = 7.9, 2.0 Hz), 7.01 (dd, 1H, *J* = 8.3, 2.4 Hz), 6.44 (d, 1H, *J* = 9.9 Hz), 5.70 (d, 1H, *J* = 9.9 Hz), 5.35 (s, 2H), 3.95 (s, 3H), 1.53 (s, 6H); ^13^C NMR (CDCl_3_, 100 MHz) *δ* 167.7, 158.6, 145.7, 145.5, 144.6, 137.5, 130.6, 130.3, 127.8, 127.3, 127.1, 126.8, 122.6, 122.6, 121.6, 121.1, 119.8, 116.9, 112.4, 110.0, 109.0, 76.6, 68.6, 52.6, 27.7; ESIMS: *m/z* 456 [M−H]^−^, HRESIMS: calcd for C_26_H_23_N_3_O_5_Na [M−H]^−^ 456.1569, found 456.1565.

*1-O-((1-(4-hydroxyphenyl)-1H-1,2,3-triazol-4-yl)methyl)-mollugin (**18**)*. Yield: 51%, yellow solid, MP: 209–211 °C, ^1^H NMR ((CD_3_)_2_CO, 400 MHz) *δ* 8.90 (s, 1H), 8.53 (s, 1H), 8.27 (m, 1H), 8.21 (m, 1H), 7.73 (d, 2H, *J* = 8.8 Hz), 7.60 (m, 2H), 7.05 (d, 2H, *J* = 8.8 Hz), 6.46 (d, 1H, *J* = 9.9 Hz), 5.85 (d, 1H, *J* = 9.9 Hz), 5.27 (s, 2H), 3.97 (s, 3H), 1.53 (s, 6H); ^13^C NMR ((CD_3_)_2_CO, 100 MHz) *δ* 167.0, 157.8, 145.6, 144.9, 144.1, 130.7, 129.9, 128.0, 127.1, 127.0, 126.5, 122.9, 122.3, 122.3, 122.2, 121.8, 119.5, 116.1, 112.4, 76.6, 68.7, 51.9, 26.9; ESIMS: *m/z* 456 [M−H]^−^, HRESIMS: calcd for C_26_H_23_N_3_O_5_Na [M−H]^−^ 456.1567, found 456.1565.

*1-O-((1-(3-chloro-4-hydroxyphenyl)-1H-1,2,3-triazol-4-yl)methyl)-mollugin (**19**).* Yield: 42%, white solid, MP: 196–198 °C, ^1^H NMR ((CD_3_)_2_CO, 400 MHz) *δ* 9.41(s,1H), 8.63 (s, 1H), 8.26 (m, 1H), 8.22 (m, 1H), 7.93 (d, 1H, *J* = 2.7 Hz), 7.75 (dd, 1H, *J* = 8.8, 2.7 Hz), 7.60 (m, 2H), 7.24 (d, 1H, *J* = 8.8 Hz), 6.46 (d, 1H, *J* = 9.9 Hz), 5.86 (d, 1H, *J* = 9.9 Hz), 5.27 (s, 2H), 3.96 (s, 3H), 1.53 (s, 6H); ^13^C NMR ((CD_3_)_2_CO, 100 MHz) *δ* 167.0, 153.4, 145.5, 144.9, 144.3, 130.7, 130.3, 128.0, 127.1, 127.1, 126.5, 122.8, 122.5, 122.4, 122.2, 121.9, 120.9, 120.7, 119.5, 117.3, 112.4, 76.6, 68.6, 51.9, 26.9; ESIMS: *m/z* 490 [M−H]^−^, HRESIMS: calcd for C_26_H_22_N_3_O_5_ClNa [M−H]^−^ 490.1174, found 490.1175.

*1-O-((1-(3-(hydroxymethyl)phenyl)-1H-1,2,3-triazol-4-yl)methyl)-mollugin (**20**).* Yield: 40%, yellow solid, MP: 151–153 °C, ^1^H NMR (CDCl_3_, 400 MHz) *δ* 8.22 (dd, 1H, *J* = 6.6, 3.1 Hz), 8.14 (dd, 1H, *J* = 6.5, 3.1 Hz), 8.10 (s, 1H), 7.76 (s, 1H)), 7.62 (dt, 1H, *J* = 8.0, 1.5 Hz), 7.51 (m, 2H), 7.46 (t, 1H, *J* = 7.8 Hz), 7.40 (d, 1H, *J* = 7.7 Hz), 6.43 (d, 1H, *J* = 9.9 Hz), 5.69 (d, 1H, *J* = 9.9 Hz), 5.30 (s, 2H), 4.78 (s, 2H), 3.93 (s, 3H), 1.52 (s, 6H); ^13^C NMR (CDCl_3_, 100 MHz) *δ* 167.7, 145.8, 145.4, 144.8, 143.4, 137.1, 130.3, 129.8, 127.9, 127.2, 127.1, 127.0, 126.8, 122.7, 122.5, 121.6, 121.1, 119.8, 119.6, 118.9, 112.4, 76.6, 69.0, 64.3, 52.5, 27.7; ESIMS: *m/z* 494 [M+Na]^+^, HRESIMS: calcd for C_27_H_25_N_3_O_5_Na [M+Na]^+^ 494.1686, found 494.1686.

*1-O-((1-(4-(hydroxymethyl)phenyl)-1H-1,2,3-triazol-4-yl)methyl)-mollugin (**21**).* Yield: 40%, yellow solid, MP: 194–196 °C, ^1^H NMR (CDCl_3_, 400 MHz) *δ* 8.22 (dd, 1H, *J* = 6.2, 3.3 Hz), 8.15 (dd, 1H, *J* = 6.1, 3.3 Hz), 8.08 (s, 1H), 7.71 (d, 2H, *J* = 8.0 Hz), 7.52 (m, 4H), 6.43 (d, 1H, *J* = 9.9 Hz), 5.70 (d, 1H, *J* = 9.9 Hz), 5.32 (s, 2H), 4.77 (s, 2H), 3.94 (s, 3H), 1.52 (s, 6H); ^13^C NMR (CDCl_3_, 100 MHz) *δ* 167.7, 145.8, 145.4, 144.9, 141.9, 136.2, 130.3, 128.1, 127.9, 127.2, 127.1, 126.8, 122.7, 122.5, 121.5, 121.1, 120.8, 119.8, 112.4, 76.6, 69.1, 64.4, 52.5, 27.7; ESIMS: *m/z* 494 [M+Na]^+^, HRESIMS: calcd for C_27_H_25_N_3_O_5_Na [M+Na]^+^ 494.1684, found 494.1686.

*1-O-((1-(2-ethylphenyl)-1H-1,2,3-triazol-4-yl)methyl)-mollugin(**22**).* Yield: 45%, yellow solid, MP: 73–75 °C, ^1^H NMR (CDCl_3_, 400 MHz) *δ* 8.21 (dd, 1H, *J* = 6.5, 3.3 Hz), 8.17 (dd, 1H, *J* = 6.4, 3.2 Hz), 7.76 (s, 1H), 7.52 (m, 2H), 7.47 (td, 1H, *J* = 7.4, 1.6 Hz), 7.47 (d, 1H, *J* = 7.4 Hz), 7.34 (td, 1H, *J* = 7.4, 1.6 Hz), 7.47 (dd, 1H, *J* = 8.0, 1.6 Hz), 6.44 (d, 1H, *J* = 9.9Hz), 5.70 (d, 1H, *J* = 9.9 Hz), 5.36 (s, 2H), 3.96 (s, 3H), 2.46 (q, 2H, *J* = 7.6 Hz), 1.52 (s, 6H), 1.12 (t, 3H, *J* = 7.5 Hz); ^13^C NMR (CDCl_3_, 100 MHz) *δ* 167.7, 145.7, 145.4, 143.7, 140.0, 135.9, 130.3, 130.2, 129.8, 128.1, 127.1, 127.0, 126.7, 126.4, 125.3, 122.4, 121.1, 119.9, 112.4, 76.6, 68.9, 52.5, 27.7, 24.1, 15.0; ESIMS: *m/z* 492 [M+Na]^+^, HRESIMS: calcd for C_28_H_27_N_3_O_4_Na [M+Na]^+^ 492.1893, found 492.1894.

*1-O-((1-(4-ethylphenyl)-1H-1,2,3-triazol-4-yl)methyl)-mollugin (**23**).* Yield: 65%, yellow solid, MP: 70–71 °C, ^1^H NMR (CDCl_3_, 400 MHz) *δ* 8.23 (dd, 1H, *J* = 6.4, 3.3 Hz), 8.17 (dd, 1H, *J* = 6.4, 3.3 Hz), 8.06 (s, 1H), 7.65 (d, 2H, *J* = 8.0 Hz), 7.53 (m, 2H), 7.35 (d, 2H, *J* = 8.0 Hz), 6.45 (d, 1H, *J* = 9.9 Hz), 5.70 (d, 1H, *J* = 9.9 Hz), 5.33 (s, 2H), 3.95 (s, 3H), 2.72 (q, 2H, *J =* 7.6 Hz), 1.53 (s, 6H), 1.28 (t, 3H, *J =* 7.8 Hz); ^13^C NMR (CDCl_3_, 100 MHz) *δ* 167.7, 145.9, 145.4, 145.3, 144.7, 134.9, 130.3, 129.1, 127.9, 127.2, 127.1, 126.8, 122.8, 122.5, 121.6, 121.1, 120.8, 119.9, 112.4, 76.6, 69.1, 52.5, 28.5, 27.7, 15.5; ESIMS: *m/z* 492 [M+Na]^+^, HRESIMS: calcd for C_28_H_27_N_3_O_4_Na [M+Na]^+^ 492.1896, found 492.1894.

*1-O-((1-(4-vinylphenyl)-1H-1,2,3-triazol-4-yl)methyl)-mollugin (**24**).* Yield: 45%, yellow solid, MP: 67–69 °C, ^1^H NMR (CDCl_3_, 400 MHz) *δ* 8.23 (dd, 1H, *J* = 6.3, 3.3 Hz), 8.17 (dd, 1H, *J* = 6.3, 3.3 Hz), 8.07 (s, 1H), 7.72 (d, 2H, 8.3 Hz), 7.56 (d, 2H, 8.6 Hz), 8.53 (m, 2H), 6.76 (dd, 1H, *J* = 17.6, 10.9 Hz), 6.44 (d, 1H, *J* = 9.9 Hz), 5.83 (d, 1H, *J* = 17.6 Hz), 5.70 (d, 1H, *J* = 9.9 Hz), 5.36 (d, 1H, *J* = 10.9 Hz), 5.34 (s, 2H), 3.95 (s, 3H), 1.53 (s, 6H); ^13^C NMR (CDCl_3_, 100 MHz) *δ* 167.6, 145.8, 145.4, 145.0, 138.2, 136.3, 135.5, 130.3, 127.9, 127.5, 127.2, 127.1, 126.8, 122.8, 122.5, 121.4, 121.1, 120.7, 119.9, 115.6, 112.4, 76.6, 69.1, 52.5, 27.7; ESIMS: *m/z* 490 [M+Na]^+^, HRESIMS: calcd for C_28_H_25_N_3_O_4_Na [M+Na]^+^ 490.1737, found 490.1737.

*1-O-((1-(3-(methylthio)phenyl)-1H-1,2,3-triazol-4-yl)methyl)-mollugin (**25**)*. Yield: 82%, yellow solid, MP: 87–89 °C, ^1^H NMR (CDCl_3_, 400 MHz) *δ* 8.23 (dd, 1H, *J* = 6.2, 3.5 Hz), 8.16 (dd, 1H, *J* = 6.6, 3.1 Hz), 8.09 (s, 1H), 7.64 (t, 1H, *J* = 1.9 Hz), 7.53 (m, 2H), 7.44 (m, 1H), 7.40 (d, 1H, *J* = 7.9 Hz), 7.30 (d, 1H, *J* = 7.9 Hz), 6.44 (d, 1H, *J* = 9.9 Hz), 5.70 (d, 1H, *J* = 9.9 Hz), 5.33 (s, 2H), 3.94(s, 3H), 2.55 (s, 3H), 1.52 (s, 6H); ^13^C NMR (CDCl_3_, 100 MHz) *δ* 167.6, 145.8, 145.4, 145.0, 141.4, 137.5, 130.3, 129.9, 127.9, 127.2, 127.1, 126.8, 126.4, 122.7, 122.5, 121.6, 121.1, 119.9, 118.0, 116.9, 112.4, 76.6, 69.0, 52.6, 27.7, 15.5; ESIMS: *m/z* 510 [M+Na]^+^, HRESIMS: calcd for C_27_H_25_N_3_O_4_SNa [M+Na]^+^ 510.1454, found 510.1458.

*1-O-((1-(dibenzo[b,d]thiophen-4-yl)-1H-1,2,3-triazol-4-yl)methyl)-mollugin (**26**).* Yield: 40%, yellow solid, MP: 149–151 °C, ^1^H NMR (CDCl_3_, 400 MHz) *δ* 8.22 (s, 1H), 8.26–8.20 (m, 4H), 7.95 (m, 1H), 7.71 (d, 1H, *J* = 7.7 Hz), 7.62 (d, 1H, *J* = 7.7 Hz), 7.57–7.50 (m, 4H), 6.46 (d, 1H, *J* = 9.9 Hz), 5.71 (d, 1H, *J* = 9.9 Hz), 5.41 (s, 2H), 3.98 (s, 3H), 1.54 (s, 6H); ^13^C NMR (CDCl_3_, 100 MHz) *δ* 167.7, 145.8, 145.5, 144.8, 140.2, 138.4, 134.8, 132.4, 132.2, 130.3, 128.0, 127.7, 127.2, 127.1, 126.8, 125.2, 124.9, 122.8, 122.8, 122.5, 122.4, 121.9, 121.8, 121.2, 119.9, 119.5, 112.4, 76.6, 69.0, 52.6, 27.7; ESIMS: *m/z* 570 [M+Na]^+^, HRESIMS: calcd for C_32_H_25_N_3_O_4_SNa [M+Na]^+^ 570.1457, found 570.1458.

*1-O-((1-(4-isopropylphenyl)-1H-1,2,3-triazol-4-yl)methyl)-mollugin (**27**)*. Yield: 87%, yellow solid, MP: 97–99 °C, ^1^H NMR (CDCl_3_, 400 MHz) *δ* 8.23 (dd, 1H, *J* = 6.3, 3.3 Hz), 8.17 (dd, 1H, *J* = 6.3, 3.4 Hz), 8.06 (s, 1H), 7.65 (d, 2H, *J* = 8.4 Hz), 7.53 (m, 2H), 7.38 (d, 2H, *J* = 8.3 Hz), 6.44 (d, 1H, *J* = 9.9Hz), 5.70 (d, 1H, *J* = 9.9 Hz), 5.33 (s, 2H), 3.94 (s, 3H), 2.99 (hept, 1H, *J* = 7.0 Hz), 1.53 (s, 6H), 1.30 (s, 3H), 1.29 (s, 3H); ^13^C NMR (CDCl_3_, 100 MHz) *δ* 167.7, 149.9, 145.9, 145.4, 144.7, 135.0, 130.3, 127.7, 127.2, 127.1, 126.8, 122.8, 122.5, 121.6, 121.1, 120.8, 119.9, 112.4, 76.6, 69.1, 52.5, 33.9, 27.7, 23.9; ESIMS: *m/z* 506 [M+Na]^+^, HRESIMS: calcd for C_29_H_29_N_3_O_4_Na [M+Na]^+^ 506.2052, found 506.2050.

*1-O-((1-(3-isopropylphenyl)-1H-1,2,3-triazol-4-yl)methyl)-mollugin (**28**).* Yield: 60%, yellow solid, MP: 55–57 °C, ^1^H NMR (CDCl_3_, 400 MHz) *δ* 8.23 (dd, 1H, *J* = 6.3, 3.3 Hz), 8.17 (dd, 1H, *J* = 6.3, 3.3 Hz), 8.09 (s, 1H), 7.63 (s, 1H), 7.57–7.47 (m, 3H), 7.44 (t, 1H, *J* = 7.8 Hz), 7.32 (d, 1H, *J* = 7.7 Hz), 6.45 (d, 1H, *J* = 9.9 Hz), 5.70 (d, 1H, *J* = 9.9 Hz), 5.34 (s, 2H), 3.95 (s, 3H), 1.53 (s, 6H), 1.32 (s, 3H), 1.30 (s, 3H); ^13^C NMR (CDCl_3_, 100 MHz) *δ* 167.6, 151.0, 145.9, 145.4, 144.8, 137.1, 130.3, 129.7, 128.0, 127.1, 127.1, 127.0, 126.8, 122.8, 122.5, 121.7, 121.1, 119.9, 119.1, 118.2, 112.4, 76.6, 69.2, 52.5, 34.2, 27.7, 23.9; ESIMS: *m/z* 506 [M+Na]^+^, HRESIMS: calcd for C_29_H_29_N_3_O_4_Na [M+Na]^+^ 506.2050, found 506.2050.

*1-O-((1-(3-(dimethylamino)phenyl)-1H-1,2,3-triazol-4-yl)methyl)-mollugin (**29**).* Yield: 60%, yellow solid, MP: 66–68 °C, ^1^H NMR (CDCl_3_, 400 MHz) *δ* 8.22 (dd, 1H, *J* = 6.3, 3.3 Hz), 8.17 (dd, 1H, *J* = 6.3, 3.3 Hz), 8.07 (s, 1H), 7.53 (m, 2H), 7.34 (t, 1H, *J* = 8.1 Hz), 7.10 (t, 1H, *J* = 2.3 Hz), 6.95 (dd, 1H, *J* = 7.8, 2.0 Hz), 6.76 (dd, 1H, *J* = 8.5, 2.5 Hz), 6.45 (d, 1H, *J* = 9.9 Hz), 5.70 (d, 1H, *J* = 9.9 Hz), 5.33 (s, 2H), 3.95 (s, 3H), 3.04 (s, 6H), 1.53 (s, 6H); ^13^C NMR (CDCl_3_, 100 MHz) *δ* 167.7, 151.4, 145.9, 145.4, 144.6, 138.1, 130.3, 130.1, 128.0, 127.1, 127.0, 126.8, 122.8, 122.5, 121.8, 121.1, 119.9, 112.5, 112.4, 108.1, 104.6, 76.6, 69.2, 52.5, 40.4, 27.7; ESIMS: *m/z* 507 [M+Na]^+^, HRESIMS: calcd for C_28_H_28_N_4_O_4_Na [M+Na]^+^ 507.2001, found 507.2003.

*1-O-((1-(2,3-dimethylphenyl)-1H-1,2,3-triazol-4-yl)methyl)-mollugin (**30**).* Yield: 75%, yellow solid, MP: 64–66 °C, ^1^H NMR (CDCl_3_, 400 MHz) *δ* 8.22–8.16 (m, 2H), 7.73 (s, 1H), 7.52 (m, 2H), 7.30 (d, 1H, *J* = 7.6 Hz), 7.21 (t, 1H, *J* = 7.7 Hz), 7.15 (d, 1H, *J* = 7.6 Hz), 6.45 (d, 1H, *J* = 9.9 Hz), 5.69 (d, 1H, *J* = 9.9 Hz), 5.36 (s, 2H), 3.96 (s, 3H), 2.36 (s, 3H), 2.00 (s, 3H), 1.52 (s, 6H); ^13^C NMR (CDCl_3_, 100 MHz) *δ* 167.7, 145.7, 145.4, 143.7, 138.8, 136.6, 132.8, 131.4, 130.3, 128.1, 127.1, 127.0, 126.7, 126.1, 125.3, 124.0, 122.9, 122.4, 121.2, 119.9, 112.4, 76.6, 68.9, 52.5, 27.7, 20.4, 14.3; ESIMS: *m/z* 492 [M+Na]^+^, HRESIMS: calcd for C_28_H_27_N_3_O_4_Na [M+Na]^+^ 492.1896, found 492.1894.

*1-O-((1-(2,5-dimethylphenyl)-1H-1,2,3-triazol-4-yl)methyl)-mollugin (**31**)*. Yield: 58%, yellow solid, MP: 59–61 °C, ^1^H NMR (CDCl_3_, 400 MHz) *δ* 8.33 (dd, 1H, *J* = 6.5, 3.3 Hz), 8.28 (dd, 1H, *J* = 6.5, 3.3 Hz), 7.87 (s, 1H), 7.64 (m, 2H), 7.36 (m, 2H), 6.57 (d, 1H, *J* = 9.9Hz), 5.82 (d, 1H, *J* = 9.9 Hz), 5.47 (s, 2H), 4.08 (s, 3H), 2.50 (s, 3H), 2.26 (s, 3H), 1.64 (s, 6H); ^13^C NMR (CDCl_3_, 100 MHz) *δ* 167.7, 145.7, 145.4, 143.7, 136.8, 136.2, 131.3, 130.6, 130.4, 130.3, 128.1, 127.1, 127.0, 126.7, 126.5, 124.9, 122.9, 122.4, 121.2, 119.9, 112.4, 76.6, 68.9, 52.5, 27.7, 20.7, 21.1; ESIMS: *m/z* 492 [M+Na]^+^, HRESIMS: calcd for C_28_H_27_N_3_O_4_Na [M+Na]^+^ 492.1896, found 492.1894.

*1-O-((1-(3,4-dimethylphenyl)-1H-1,2,3-triazol-4-yl)methyl)-mollugin (**32**).* Yield: 75%, yellow solid, MP: 76–78 °C, ^1^H NMR (CDCl_3_, 400 MHz) *δ* 8.22 (dd, 1H, *J* = 6.4, 3.4 Hz), 8.17 (dd, 1H, *J* = 6.3, 3.3 Hz), 8.04 (s, 1H), 7.52 (m, 3H), 7.43 (dd, 1H, *J* = 8.0, 3.3 Hz), 7.26 (d, 1H, *J* = 8.0 Hz), 6.45 (d, 1H, *J* = 10.0 Hz), 5.70 (d, 1H, *J* = 10.0 Hz), 5.33 (s, 2H), 3.95 (s, 3H), 2.35 (s, 3H), 2.32 (s, 3H), 1.53 (s, 6H); ^13^C NMR (CDCl_3_, 100 MHz) *δ* 167.6, 145.9, 145.4, 144.7, 138.4, 137.6, 135.0, 130.7, 130.3, 128.0, 127.1, 127.0, 126.8, 122.8, 122.5, 121.9, 121.5, 121.1, 119.9, 118.0, 112.4, 76.6, 69.2, 52.5, 27.7, 19.9, 19.5; ESIMS: *m/z* 492 [M+Na]^+^, HRESIMS: calcd for C_28_H_27_N_3_O_4_Na [M+Na]^+^ 492.1896, found 492.1894.

*1-O-((1-(5-fluoro-2-methylphenyl)-1H-1,2,3-triazol-4-yl)methyl)-mollugin (**33**).* Yield: 65%, white solid, MP: 61–63 °C, ^1^H NMR (CDCl_3_, 400 MHz) *δ* 8.21 (dd, 1H, *J* = 6.4, 3.3 Hz), 8.15 (dd, 1H, *J* = 6.4, 3.3 Hz), 7.76 (s, 1H), 7.52 (m, 2H), 7.32 (dd, 1H, *J* = 8.2, 5.9 Hz), 7.17–7.07 (m, 2H), 6.44 (d, 1H, *J* = 9.9 Hz), 5.70 (d, 1H, *J* = 9.9 Hz), 5.35 (s, 2H), 3.95 (s, 3H), 2.16 (s, 3H), 1.52 (s, 6H); ^13^C NMR (CDCl_3_, 100 MHz) *δ* 167.6, 162.0, 159.6, 145.5, 143.9, 132.7, 136.9, 130.3, 129.3, 128.0, 127.1, 127.1, 126.8, 124.9, 122.8, 122.5, 121.2, 119.8, 116.9, 113.5, 112.4, 76.6, 68.7, 52.6, 27.7, 17.4; ESIMS: *m/z* 472 [M−H]^−^, HRESIMS: calcd for C_27_H_24_N_3_O_4_FNa [M−H]^−^ 472.1675, found 472.1678.

*1-O-((1-(2-chloro-4-methylphenyl)-1H-1,2,3-triazol-4-yl)methyl)-mollugin (**34**)*. Yield: 92%, yellow solid, MP: 55–57 °C, ^1^H NMR (CDCl_3_, 400 MHz) *δ* 8.22 (dd, 1H, *J* = 6.5, 3.4 Hz), 8.16 (dd, 1H, *J* = 6.5, 3.3 Hz), 8.00 (s, 1H), 7.51 (m, 3H), 7.38 (s, 1H), 7.23 (d, 1H, *J* = 8.0 Hz), 6.44 (d, 1H, *J* = 9.9Hz), 5.69 (d, 1H, *J* = 9.9 Hz), 5.35 (s, 2H), 3.96 (s, 3H), 2.42 (s, 3H), 1.52 (s, 6H); ^13^C NMR (CDCl_3_, 100 MHz) *δ* 167.7, 145.8, 145.4, 143.8, 141.6, 132.4, 131.0, 130.3, 128.6, 128.3, 128.1, 127.5, 127.1, 127.0, 126.8, 125.5, 122.8, 122.4, 121.1, 119.9, 112.4, 76.6, 68.9, 52.6, 27.7, 21.1; ESIMS: *m/z* 512 [M+Na]^+^, HRESIMS: calcd for C_27_H_24_N_3_O_4_ClNa [M+Na]^+^ 512.1343, found 512.1348.

*1-O-((1-(4-chlorophenyl)-1H-1,2,3-triazol-4-yl)methyl)-mollugin (**35**).* Yield: 20%, white solid, MP: 160–162 °C, ^1^H NMR (CDCl_3_, 400 MHz) *δ* 8.23 (dd, 1H, *J* = 6.5, 3.3 Hz), 8.15 (dd, 1H, *J* = 6.5, 3.3 Hz), 8.07 (s, 1H), 7.71 (d, 2H, *J* = 8.7 Hz), 7.54–7.48 (m, 4H), 6.44 (d, 1H, *J* = 9.9 Hz), 5.70 (d, 1H, *J* = 9.9 Hz), 5.33 (s, 2H), 3.94 (s, 3H), 1.53 (s, 6H); ^13^C NMR (CDCl_3_, 100 MHz) *δ* 167.8, 145.9, 145.7, 145.4, 135.8, 134.9, 130.5, 130.2, 128.0, 127.4, 127.3, 127.0, 122.9, 122.8, 122.1, 121.7, 121.3, 120.0, 112.6, 76.8, 69.2, 52.7, 27.9; ESIMS: *m/z* 498 [M+Na]^+^, HRESIMS: calcd for C_26_H_22_N_3_O_4_ClNa [M+Na]^+^ 498.1194, found 498.1191.

*1-O-((1-(4-(trifluoromethyl)phenyl)-1H-1,2,3-triazol-4-yl)methyl)-mollugin (**36**).* Yield: 15%, white solid, MP: 168–170 °C, ^1^H NMR (CDCl_3_, 400 MHz) *δ* 8.23 (dd, 1H, *J* = 6.5, 3.3 Hz), 8.15 (m, 2H), 7.93 (d, 2H, *J* = 8.4 Hz), 7.83 (d, 2H, *J* = 8.3 Hz), 7.54 (m, 2H), 6.44 (d, 1H, *J* = 9.9 Hz), 5.71 (d, 1H, *J* = 9.9 Hz), 5.35 (s, 2H), 3.94 (s, 3H), 1.53 (s, 6H); ^13^C NMR (CDCl_3_, 150 MHz) *δ* 167.8, 145.9, 145.7, 145.4, 139.7, 131.2, 130.6, 130.2, 128.1, 127.4, 127.4, 127.3, 127.0, 122.8, 122.8, 122.1, 121.7, 121.6, 121.3, 120.9, 120.0, 112.6, 76.8, 69.1, 52.7, 27.9; ESIMS: *m/z* 532 [M+Na]^+^, HRESIMS: calcd for C_27_H_22_N_3_O_4_F_3_Na [M+Na]^+^ 532.1453, found 532.1455.

*1-O-((1-(3-(trifluoromethoxy)phenyl)-1H-1,2,3-triazol-4-yl)methyl)-mollugin (**37**).* Yield: 15%, white solid, MP: 161–163 °C, ^1^H NMR (CDCl_3_, 400 MHz) *δ* 8.23 (dd, 1H, *J* = 6.5, 3.3 Hz), 8.15 (dd, 1H, *J* = 6.4, 3.3 Hz), 8.10 (s, 1H), 7.75–7.66 (m, 2H), 7.59 (t, 1H, *J* = 8.1 Hz), 7.53 (m, 2H), 7.33 (d, 1H, *J* = 8.3 Hz), 6.44 (d, 1H, *J* = 9.9 Hz), 5.71 (d, 1H, *J* = 9.9 Hz), 5.34 (s, 2H), 3.95 (s, 3H), 1.53 (s, 6H); ^13^C NMR (CDCl_3_, 150 MHz) *δ* 167.8, 150.2, 145.9, 145.7, 145.6, 138.3, 131.4, 130.5, 128.1, 127.4, 127.3, 127.0, 122.9, 122.8, 121.7, 121.3, 121.2, 120.0, 118.9, 113.9, 112.6, 76.8, 69.2, 52.7, 27.9; ESIMS: *m/z* 548 [M+Na]^+^, HRESIMS: calcd for C_27_H_22_N_3_O_5_F_3_Na [M+Na]^+^ 548.1401, found 548.1404.

*1-O-((1-(4-(trifluoromethoxy)phenyl)-1H-1,2,3-triazol-4-yl)methyl)-mollugin (**38**).* Yield: 20%, white solid, MP: 157–159 °C, ^1^H NMR (CDCl_3_, 400 MHz) *δ* 8.23 (dd, 1H, *J* = 6.4, 3.3 Hz), 8.15 (dd, 1H, *J* = 6.5, 3.3 Hz), 8.08 (s, 1H), 7.81 (d, 2H, *J* = 8.6 Hz), 7.53 (m, 2H), 7.40 (d, 2H, *J* = 8.4 Hz), 6.44 (d, 1H, *J* = 9.9 Hz), 5.70 (d, 1H, *J* = 9.9 Hz), 5.34 (s, 2H), 3.94 (s, 3H), 1.53 (s, 6H); ^13^C NMR (CDCl_3_, 100 MHz) *δ* 167.6, 149.1, 145.7, 145.5, 145.3, 135.4, 130.3, 127.9, 127.2, 127.1, 126.8, 122.7, 122.6, 122.3, 122.2, 121.6, 121.1, 119.8, 112.4, 76.6, 69.0, 52.5, 27.7; ESIMS: *m/z* 548 [M+Na]^+^, HRESIMS: calcd for C_27_H_22_N_3_O_5_F_3_Na [M+Na]^+^ 548.1408, found 548.1404.

*1-O-((1-(2,3-dichlorophenyl)-1H-1,2,3-triazol-4-yl)methyl)-mollugin (**39**).* Yield: 70%, yellow solid, MP: 66–68 °C, ^1^H NMR (CDCl_3_, 400 MHz) *δ* 8.21 (dd, 1H, *J* = 6.4, 3.3 Hz), 8.15 (dd, 1H, *J* = 6.3, 3.3 Hz), 8.01 (s, 1H), 7.63 (dd, 1H, *J* = 8.1, 1.6 Hz), 7.56–7.47 (m, 3H), 7.38 (t, 1H, *J* = 8.1 Hz), 6.44 (d, 1H, *J* = 9.9 Hz), 5.69 (d, 1H, *J* = 9.9 Hz), 5.36 (s, 2H), 3.96 (s, 3H), 1.52 (s, 6H); ^13^C NMR (CDCl_3_, 100 MHz) *δ* 167.6, 145.6, 145.4, 143.9, 136.4, 134.6, 131.7, 130.3, 128.2, 128.0, 127.9, 127.1, 127.0, 126.8, 126.3, 125.5, 122.8, 122.5, 121.2, 119.8, 112.4, 76.6, 68.7, 52.5, 27.7; ESIMS: *m/z* 532 [M+Na]^+^, HRESIMS: calcd for C_26_H_21_N_3_O_4_Cl_2_Na [M+Na]^+^ 532.0802, found 532.0801.

*1-O-((1-(3-chloro-4-fluorophenyl)-1H-1,2,3-triazol-4-yl)methyl)-mollugin (**40**)*. Yield: 30%, white solid, MP: 179–181 °C, ^1^H NMR (CDCl_3_, 400 MHz) *δ* 8.23 (dd, 1H, *J* = 6.4, 3.4 Hz), 8.13 (dd, 1H, *J* = 6.4, 3.3 Hz), 8.03 (s, 1H), 7.86 (dd, 1H, *J* = 6.3, 2.7 Hz), 7.64 (m, 1H), 7.53 (m, 2H), 7.31 (t, 1H, *J* = 8.6 Hz), 6.44 (d, 1H, *J* = 9.9 Hz), 5.70 (d, 1H, *J* = 9.9Hz), 5.33 (s, 1H), 3.94 (s, 3H), 1.53 (s, 6H); ^13^C NMR (CDCl_3_, 100 MHz) *δ* 167.7, 156.8, 145.7, 145.5, 145.3, 133.6, 130.3, 127.8, 127.2, 127.1, 126.8, 123.3, 122.7, 122.6, 121.6, 121.1, 120.5, 119.8, 117.8, 117.6, 112.4, 76.6, 68.9, 52.5, 27.7; ESIMS: *m/z* 516 [M+Na]^+^, HRESIMS: calcd for C_26_H_21_N_3_O_4_FClNa [M+Na]^+^ 516.1096, found 516.1097.

*1-O-((1-(3-chloro-5-(trifluoromethyl)phenyl)-1H-1,2,3-triazol-4-yl)methyl)-mollugin (**41**)*. Yield: 17%, white solid, MP: 171–173 °C, ^1^H NMR (CDCl_3_, 400 MHz) *δ* 8.24 (dd, 1H, *J* = 6.5, 3.3 Hz), 8.16–8.08 (m, 2H), 8.02 (m, 1H), 7.94 (m, 1H), 7.71 (s, 1H), 7.54 (m, 2H), 6.44 (d, 1H, *J* = 9.9 Hz), 5.71 (d, 1H, *J* = 9.9Hz), 5.35 (s, 2H), 3.94 (s, 3H), 1.53 (s, 6H); ^13^C NMR (CDCl_3_, 100 MHz) *δ* 167.8, 145.9, 145.8, 138.4, 136.9, 134.1, 133.8, 130.6, 128.0, 127.4, 127.3, 127.1, 125.9, 124.1, 122.8, 122.7, 121.6, 121.4, 120.0, 115.9, 112.6, 76.8, 69.1, 52.8, 29.9, 27.9; ESIMS: *m/z* 566 [M+Na]^+^, HRESIMS: calcd for C_27_H_21_N_3_O_4_F_3_ClNa [M+Na]^+^ 566.1066, found 566.1065.

*1-O-((1-(3,5-bis(trifluoromethyl)phenyl)-1H-1,2,3-triazol-4-yl)methyl)-mollugin (**42**).* Yield: 20%, white solid, MP: 160–162 °C, ^1^H NMR (CDCl_3_, 400 MHz) *δ* 8.28–8.10 (m, 3H), 8.19 (s, 1H), 8.12 (dd, 1H, *J* = 6.5, 3.2 Hz), 7.97 (s, 1H), 7.54 (m, 2H), 6.44 (d, 1H, *J* = 9.9 Hz), 5.71 (d, 1H, *J* = 9.9 Hz), 5.36 (s, 2H), 3.94 (s, 3H), 1.53 (s, 6H); ^13^C NMR (CDCl_3_, 100 MHz) *δ* 167.6, 145.9, 145.6, 145.5, 138.0, 134.2, 133.8, 133.5, 133.2, 130.4, 127.8, 127.2, 127.1, 126.9, 123.9, 122.6, 122.5, 122.3, 121.4, 121.2, 120.6, 119.8, 112.4, 76.6, 68.7, 52.5, 27.7; ESIMS: *m/z* 600 [M+Na]^+^, HRESIMS: calcd for C_28_H_21_N_3_O_4_F_6_Na [M+Na]^+^ 600.1327, found 600.1328.

*1-O-((1-(4-cyanophenyl)-1H-1,2,3-triazol-4-yl)methyl)-mollugin (**43**)*. Yield: 47%, white solid, MP: 167–169 °C, ^1^H NMR (CDCl_3_, 400 MHz) *δ* 8.23 (dd, 1H, *J* = 6.4, 3.4 Hz), 8.17 (s, 1H), 8.12 (dd, 1H, *J* = 6.4, 3.3 Hz), 7.92 (d, 2H, *J* = 8.7 Hz), 7.84 (d, 2H, *J* = 8.7 Hz), 7.53 (m, 2H), 6.43 (d, 1H, *J* = 9.9Hz), 5.70 (d, 1H, *J* = 9.9 Hz), 5.34 (s, 2H), 3.93 (s, 3H), 1.53 (s, 6H); ^13^C NMR (CDCl_3_, 100 MHz) *δ* 167.6, 145.7, 145.6, 145.5, 139.8, 134.0, 130.4, 127.8, 127.2, 127.1, 126.8, 122.6, 122.5, 121.2, 121.1, 120.7, 119.8, 117.8, 112.5, 112.4, 76.6, 68.8, 52.5, 27.7; ESIMS: *m/z* 489 [M+Na]^+^, HRESIMS: calcd for C_29_H_29_N_3_O_4_Na [M+Na]^+^ 489.1536, found 489.1533.

*1-O-((1-(3-cyanophenyl)-1H-1,2,3-triazol-4-yl)methyl)-mollugin (**44**).* Yield: 35%, white solid, MP: 187–189 °C, ^1^H NMR (CDCl_3_, 400 MHz) *δ* 8.23 (dd, 1H, *J* = 6.5, 3.3 Hz), 8.12–8.20 (m, 4H), 7.74 (d, 1H, *J* = 7.7 Hz), 7.67 (t, 1H, *J* = 7.9 Hz), 7.53 (m, 2H), 6.43 (d, 1H, *J* = 9.9 Hz), 5.71 (d, 1H, *J* = 9.9 Hz), 5.34 (s, 2H), 3.94 (s, 3H), 1.53 (s, 6H); ^13^C NMR (CDCl_3_, 100 MHz) *δ* 167.6, 145.6, 145.5, 137.6, 132.2, 130.9, 130.4, 127.8, 127.2, 127.1, 126.8, 124.6, 123.8, 122.6, 121.4, 121.1, 119.8, 117.4, 114.2, 112.4, 76.6, 68.8, 52.5, 27.7; ESIMS: *m/z* 489 [M+Na]^+^, HRESIMS: calcd for C_27_H_22_N_4_O_4_Na [M+Na]^+^ 489.1532, found 489.1533.

### 3.3. Biological Assays

The following human cancer cell lines were used: HL-60, A-549, SMMC-7721, MCF-7, and SW-480. These cells were obtained from American type culture collection (ATCC) (Manassas, VA, USA). All the cells were cultured in RPMI-1640 or Dulbecco’s modified Eagle medium (DMEM) medium (Biological Industries, Kibbutz Beit-Haemek, Israel), supplemented with 10% fetal bovine serum at 37 °C in a humidified atmosphere with 5% CO_2_. Cell viability was assessed by conducting colorimetric measurements of the amount of insoluble formazan formed in living cells based on the reduction of 3-(4,5-dimethylthiazol-2-yl)-5-(3-carboxymethoxyphenyl)-2-(4-sulfophenyl)-2H-tetrazolium, inner salt (MTS) (Promega, Madison, WI, USA). Briefly, cells were seeded into each well of a 96-well cell culture plate. After 12 h of incubation at 37 °C, the test compound (40 μM) was added. After incubated for 48 h, cells were subjected to the MTS assay. Compounds with a growth inhibition rate of 50% were further evaluated at concentrations of 0.064, 0.32, 1.6, 8, and 40 μM in triplicate, with cisplatin and paclitaxel (MeilunBio) as positive controls. After the incubation, MTS (20 μL) was added to each well and the incubation continued for 4 h at 37 °C. After sufficient reaction, the light absorption value of each well was read by Multiskan FC at 492 nm. The IC_50_ value of each compound was calculated with Reed and Muench’s method.

## 4. Conclusions

In conclusion, 40 1-substituted 1,2,3-triazole-mollugin derivatives were synthesized through Huisgen 1,3-dipolar cycloaddition reaction and evaluated for cytotoxicity against a series of five different human cancer cell lines (HL-60, A549, SMMC-7721, SW480, and MCF-7) along with the parent molecule. Most of the derivatives showed better cytotoxicity than parent molecule. It is worth mentioning that our experiment results showed that compound **14** and **17** exhibited cytotoxicity of all five cancer cell lines significantly and compound 36 could enhance the cytotoxicity of lung cancer cells (A549) specifically. Structure and activity relationship (SAR) analysis reveals that electron-donating groups including hydroxyl, methoxy, and alcohol hydroxyl groups are essential for retaining the cytotoxicity to derivatives. In addition, for derivatives containing methoxy groups that the cytotoxicity may increase with the number of methoxy groups. Based on the SAR studies, we believe that the enhancement of cytotoxicity of the derivatives may be caused by the aromatic ring becoming electron-rich or the electron-donating atoms with lone pairs provided by electron-donating groups available to serve as hydrogen bond acceptors with the active site, which is worthy of further study.

## Figures and Tables

**Table 1:** IC_50_ value in μM of mollugin and its derivatives on the panel of human cancer cell lines.

	Tissue	Leukemia	Lung	Liver	Breast	Colon
	Cell Line	HL-60	A549	SMMC-7721	MCF-7	SW480
No.	Compound	IC_50_				
1	**1**	>40	>40	>40	>40	>40
2	**5**	28.70 ± 0.49	>40	19.28 ± 1.48	>40	>40
3	**6**	>40	>40	24.28 ± 1.47	>40	>40
4	**7**	>40	>40	33.96 ± 0.93	>40	>40
5	**8**	>40	>40	>40	>40	>40
6	**9**	>40	>40	11.19 ± 1.56	27.71 ± 1.06	>40
7	**10**	>40	>40	>40	>40	>40
8	**11**	19.17 ± 1.40	>40	12.97 ± 1.52	10.25 ± 1.28	>40
9	**12**	>40	>40	28.11 ± 0.79	>40	>40
10	**13**	>40	>40	30.13 ± 0.44	>40	>40
11	**14**	7.03 ± 0.19	5.12 ± 0.01	10.76 ± 0.10	13.91 ± 0.51	19.56 ± 0.38
12	**15**	>40	>40	18.25 ± 0.68	29.80 ± 0.84	>40
13	**16**	35.21 ± 2.66	16.90 ± 0.66	18.33 ± 0.18	19.69 ± 1.05	26.79 ± 0.89
14	**17**	16.38 ± 0.47	15.09 ± 1.00	12.61 ± 0.80	14.49 ± 0.49	17.40 ± 0.91
15	**18**	11.00 ± 0.12	29.62 ± 0.91	12.06 ± 0.59	22.25 ± 0.37	33.42 ± 0.64
16	**19**	10.50 ± 0.02	24.12 ± 0.34	12.98 ± 0.54	21.76 ± 0.77	33.77 ± 0.54
17	**20**	25.07 ± 0.49	29.01 ± 0.76	13.02 ± 0.84	15.58 ± 0.34	25.59 ± 1.70
18	**21**	21.76 ± 0.27	>40	12.80 ± 0.34	22.50 ± 1.14	>40
19	**22**	>40	>40	32.25 ± 0.83	>40	>40
20	**23**	>40	>40	>40	>40	>40
21	**24**	>40	>40	>40	>40	>40
22	**25**	>40	>40	27.84 ± 0.54	>40	>40
23	**26**	>40	>40	>40	>40	>40
24	**27**	>40	>40	>40	>40	>40
25	**28**	>40	>40	>40	21.74 ± 1.06	>40
26	**29**	>40	>40	17.89 ± 0.27	23.44 ± 1.31	>40
27	**30**	>40	>40	30.52 ± 0.46	>40	>40
28	**31**	>40	>40	30.01 ± 0.82	>40	>40
29	**32**	>40	>40	>40	>40	>40
30	**33**	>40	>40	31.56 ± 0.51	>40	>40
31	**34**	>40	>40	>40	>40	>40
32	**35**	>40	29.52 ± 0.29	>40	>40	>40
33	**36**	>40	4.82 ± 0.84	21.66 ± 0.89	>40	>40
34	**37**	>40	>40	>40	>40	>40
35	**38**	>40	>40	>40	>40	>40
36	**39**	>40	>40	>40	>40	>40
37	**40**	>40	>40	>40	>40	>40
38	**41**	>40	>40	>40	>40	>40
39	**42**	>40	>40	>40	>40	>40
40	**43**	>40	>40	>40	>40	>40
41	**44**	>40	>40	>40	>40	>40
42	DDP	1.312 ± 0.024	17.18 ± 1.36	19.97 ± 0.26	20.63 ± 0.64	15.50 ± 0.99
43	Taxol	<0.008	<0.008	0.388 ± 0.042	<0.008	<0.008

## Data Availability

NMR spectra of synthesized compounds. See electronic Supplementary Information.

## Supplementary Materials

The following are available online. NMR spectra.

## Sample Availability

Samples of the compounds 5–44 are available from the authors.
