# Supplementary material for: Synthesis and Antitumor Activity of 1-Substituted 1,2,3-Triazole-Mollugin Derivatives
Source: Molecules. 2021 May 28;26(11):3249. doi: 10.3390/molecules26113249 (PMC8198100; doi:10.3390/molecules26113249)
Supplement: Supplementary file 1 [file molecules-26-03249-s001.zip › molecules-1223965-supplementary.pdf]

Supporting Information

Synthesis and antitumor activity of 1-substituted 1,2,3-triazole-mollugin derivatives

Han Luo,[1†] Yong-Feng Lv [2†], Hong Zhang [1], Jiang-Miao Hu [2] Hong-Mei Li[2] and Shou-Jin Liu *[1]

[1] *College of Pharmacy, Anhui University of Chinese Medicine, Hefei 230011, People' s Republic of China*

[2] *State Key Laboratory of Phytochemistry and Plant Resources in West China, Kunming Institute of Botany, Chinese Academy of Sciences, Kunming 650201, People' s Republic of China*

* Corresponding author, email: shjinliu@sina.com

† Han Luo and Yong-Feng Lv contributed equally to this work.

List of supplementary material Pages

**Figure 1**	^{1}H NMR spectrum and ^{13}C NMR spectrum of compound **5**	1
**Figure 2**	^{1}H NMR spectrum and ^{13}C NMR spectrum of compound **6**	2
**Figure 3**	^{1}H NMR spectrum and ^{13}C NMR spectrum of compound **7**	3
**Figure 4**	^{1}H NMR spectrum and ^{13}C NMR spectrum of compound **8**	4
**Figure 5**	^{1}H NMR spectrum and ^{13}C NMR spectrum of compound **9**	5
**Figure 6**	^{1}H NMR spectrum and ^{13}C NMR spectrum of compound **10**	6
**Figure 7**	^{1}H NMR spectrum and ^{13}C NMR spectrum of compound **11**	7
**Figure 8**	^{1}H NMR spectrum and ^{13}C NMR spectrum of compound **12**	8
**Figure 9**	^{1}H NMR spectrum and ^{13}C NMR spectrum of compound **13**	9
**Figure 10**	^{1}H NMR spectrum and ^{13}C NMR spectrum of compound **14**	10
**Figure 11**	^{1}H NMR spectrum and ^{13}C NMR spectrum of compound **15**	11
**Figure 12**	^{1}H NMR spectrum and ^{13}C NMR spectrum of compound **16**	12
**Figure 13**	^{1}H NMR spectrum and ^{13}C NMR spectrum of compound **17**	13
**Figure 14**	^{1}H NMR spectrum and ^{13}C NMR spectrum of compound **18**	14

**Figure 15**	^{1}H NMR spectrum and ^{13}C NMR spectrum of compound **19**	15
**Figure 16**	^{1}H NMR spectrum and ^{13}C NMR spectrum of compound **20**	16
**Figure 17**	^{1}H NMR spectrum and ^{13}C NMR spectrum of compound **21**	17
**Figure 18**	^{1}H NMR spectrum and ^{13}C NMR spectrum of compound **22**	18
**Figure 19**	^{1}H NMR spectrum and ^{13}C NMR spectrum of compound **23**	19
**Figure 20**	^{1}H NMR spectrum and ^{13}C NMR spectrum of compound **24**	20
**Figure 21**	^{1}H NMR spectrum and ^{13}C NMR spectrum of compound **25**	21
**Figure 22**	^{1}H NMR spectrum and ^{13}C NMR spectrum of compound **26**	22
**Figure 23**	^{1}H NMR spectrum and ^{13}C NMR spectrum of compound **27**	23
**Figure 24**	^{1}H NMR spectrum and ^{13}C NMR spectrum of compound **28**	24
**Figure 25**	^{1}H NMR spectrum and ^{13}C NMR spectrum of compound **29**	25
**Figure 26**	^{1}H NMR spectrum and ^{13}C NMR spectrum of compound **30**	26
**Figure 27**	^{1}H NMR spectrum and ^{13}C NMR spectrum of compound **31**	27
**Figure 28**	^{1}H NMR spectrum and ^{13}C NMR spectrum of compound **32**	28
**Figure 29**	^{1}H NMR spectrum and ^{13}C NMR spectrum of compound **33**	29
**Figure 30**	^{1}H NMR spectrum and ^{13}C NMR spectrum of compound **34**	30
**Figure 31**	^{1}H NMR spectrum and ^{13}C NMR spectrum of compound **35**	31
**Figure 32**	^{1}H NMR spectrum and ^{13}C NMR spectrum of compound **36**	32
**Figure 33**	^{1}H NMR spectrum and ^{13}C NMR spectrum of compound **37**	33
**Figure 34**	^{1}H NMR spectrum and ^{13}C NMR spectrum of compound **38**	34
**Figure 35**	^{1}H NMR spectrum and ^{13}C NMR spectrum of compound **39**	35
**Figure 36**	^{1}H NMR spectrum and ^{13}C NMR spectrum of compound **40**	36
**Figure 37**	^{1}H NMR spectrum and ^{13}C NMR spectrum of compound **41**	37
**Figure 38**	^{1}H NMR spectrum and ^{13}C NMR spectrum of compound **42**	38
**Figure 39**	^{1}H NMR spectrum and ^{13}C NMR spectrum of compound **43**	39
**Figure 40**	^{1}H NMR spectrum and ^{13}C NMR spectrum of compound **44**	40

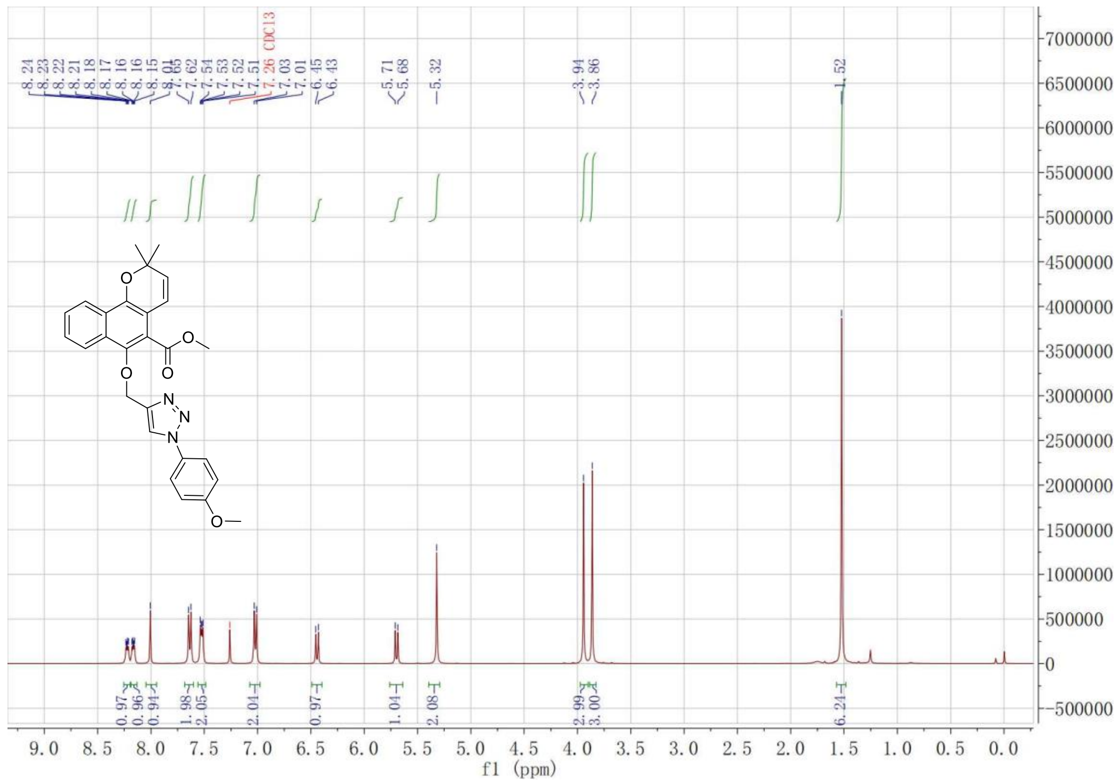

Figure 1-1. ^{1}H NMR spectrum of compound **5**

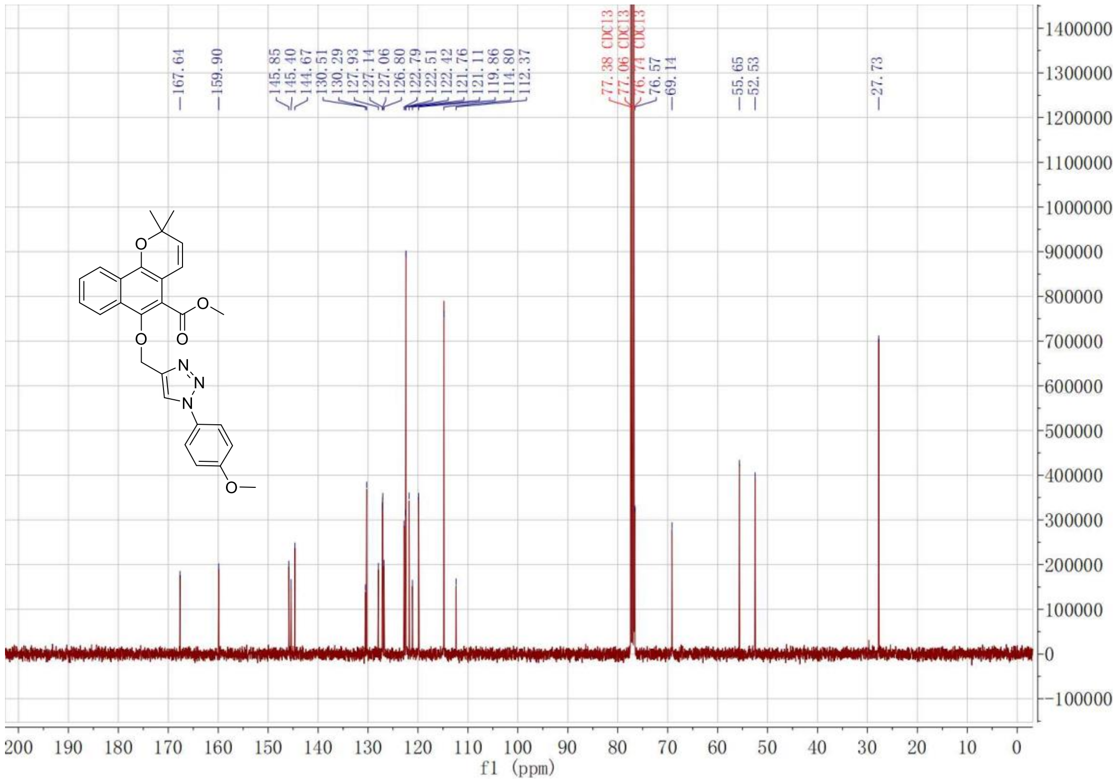

Figure 1-2. ^{13}C NMR spectrum of compound **5**

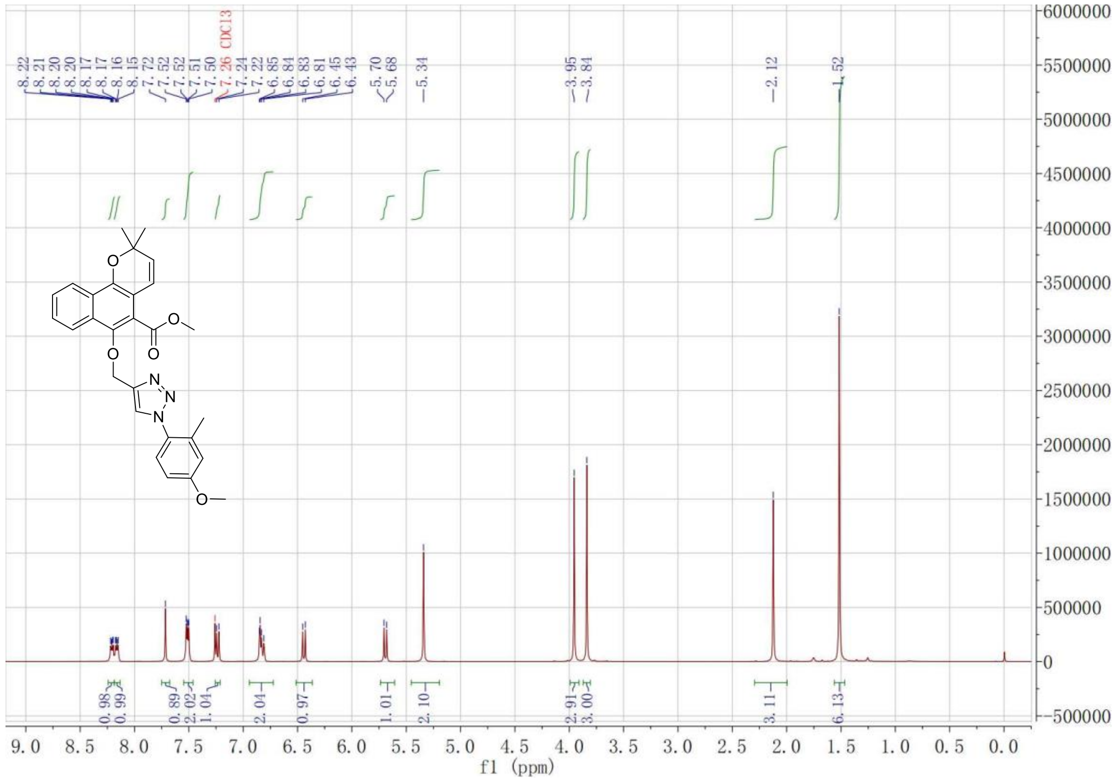

Figure 2-1. ^{1}H NMR spectrum of compound **6**

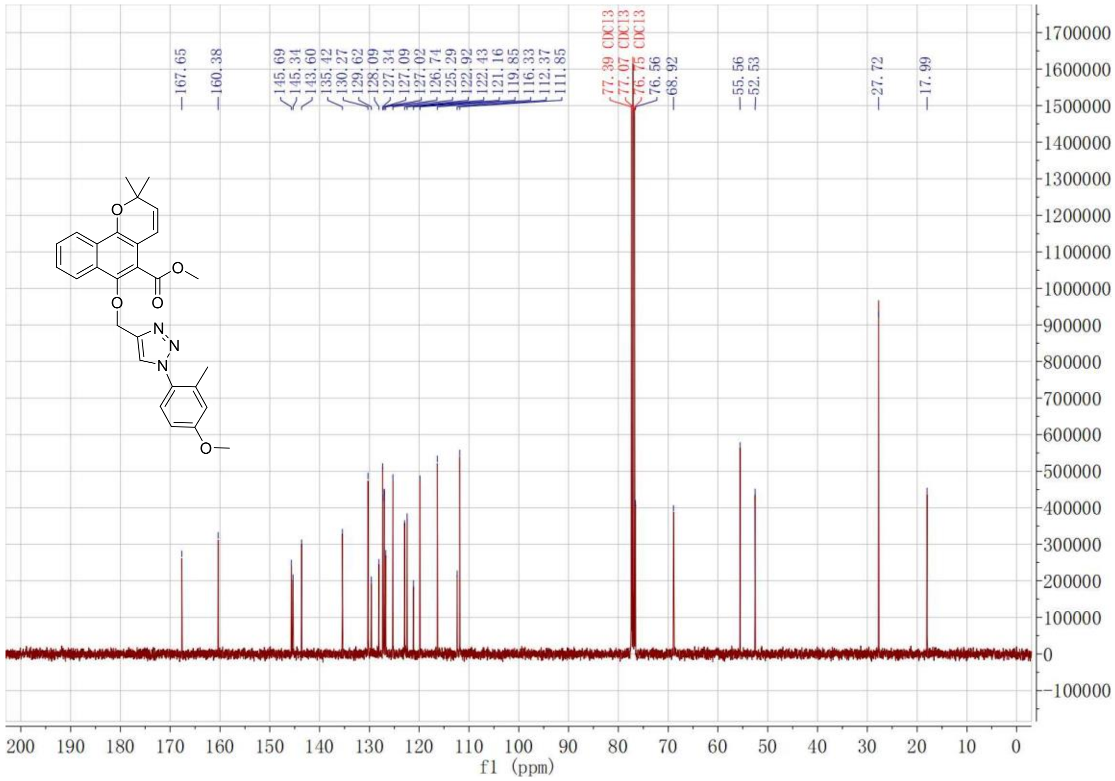

Figure 2-2. ^{13}C NMR spectrum of compound **6**

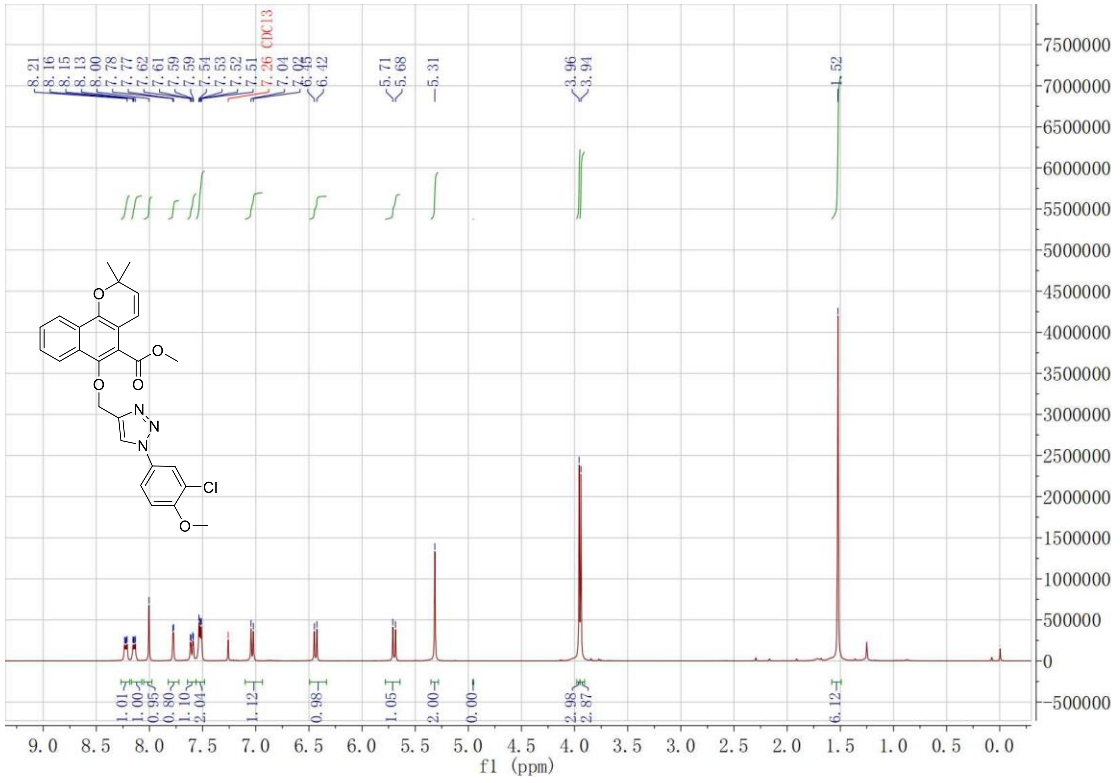

Figure 3-1. ^{1}H NMR spectrum of compound **7**

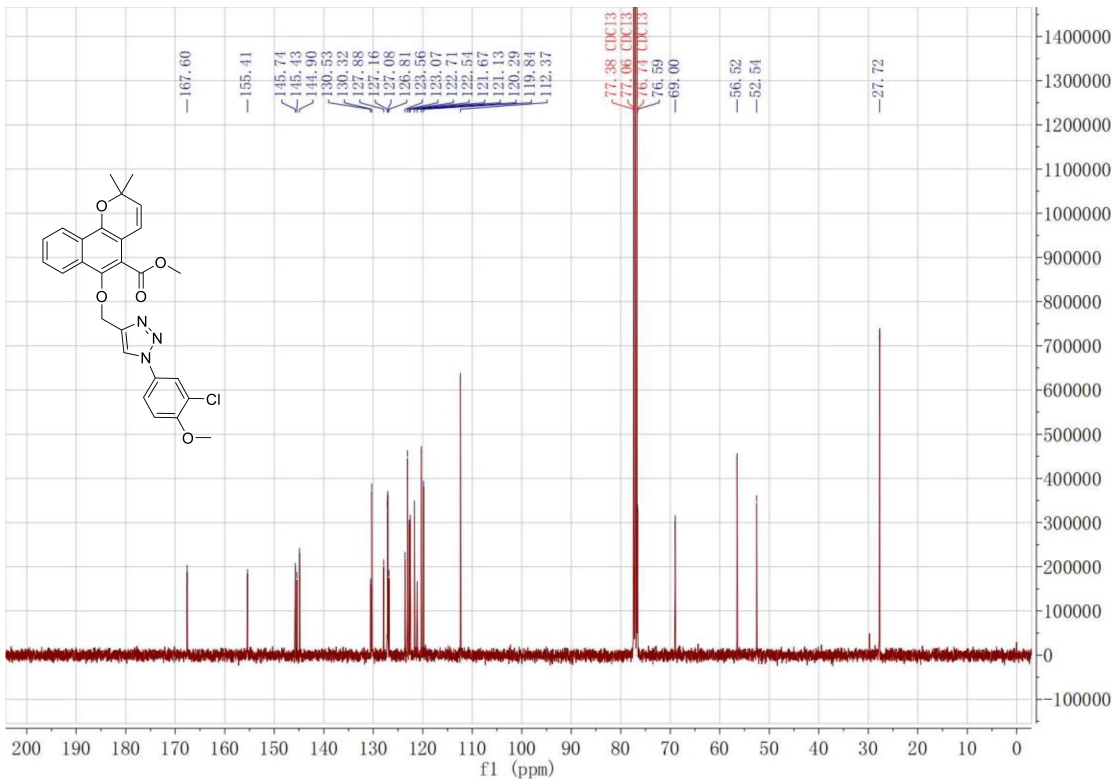

Figure 3-2. ^{13}C NMR spectrum of compound **7**

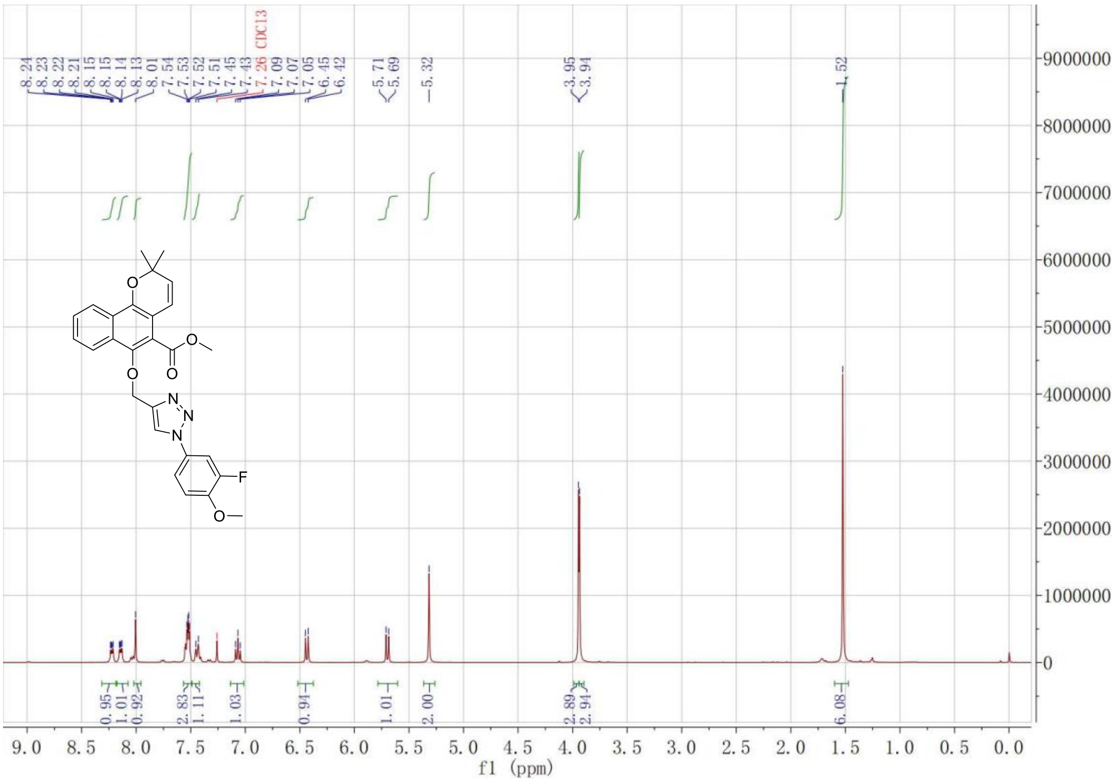

Figure 4-1. ^{1}H NMR spectrum of compound **8**

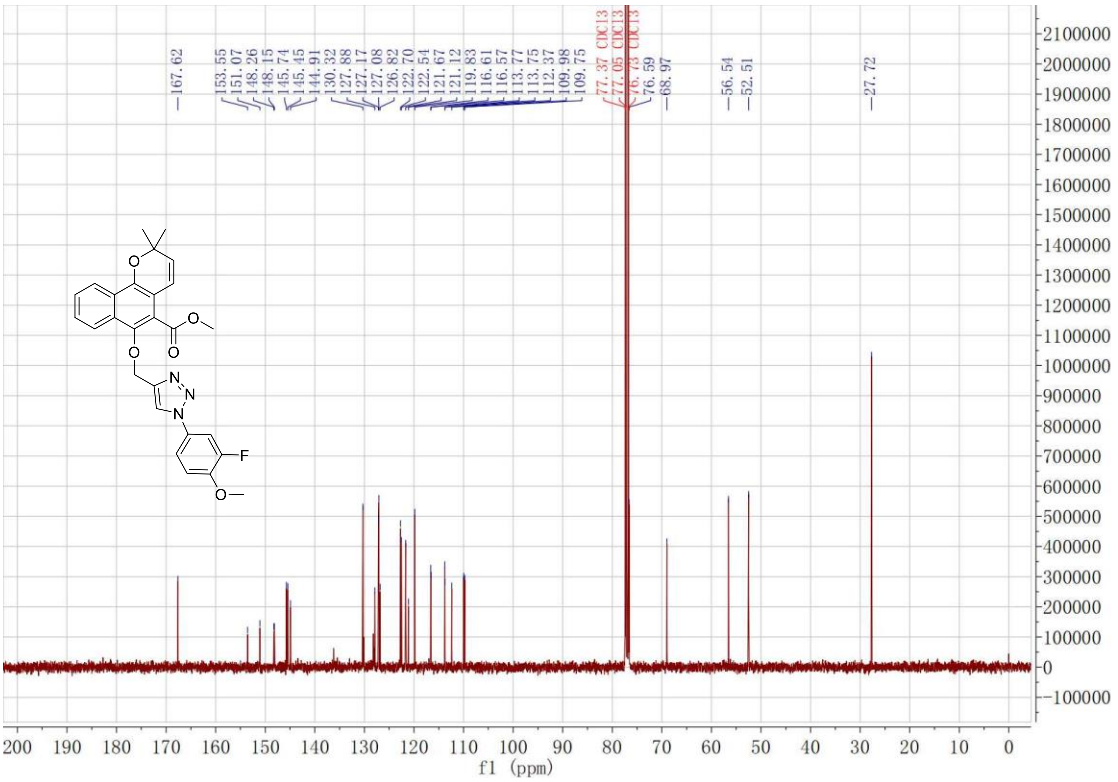

Figure 4-2. ^{13}C NMR spectrum of compound **8**

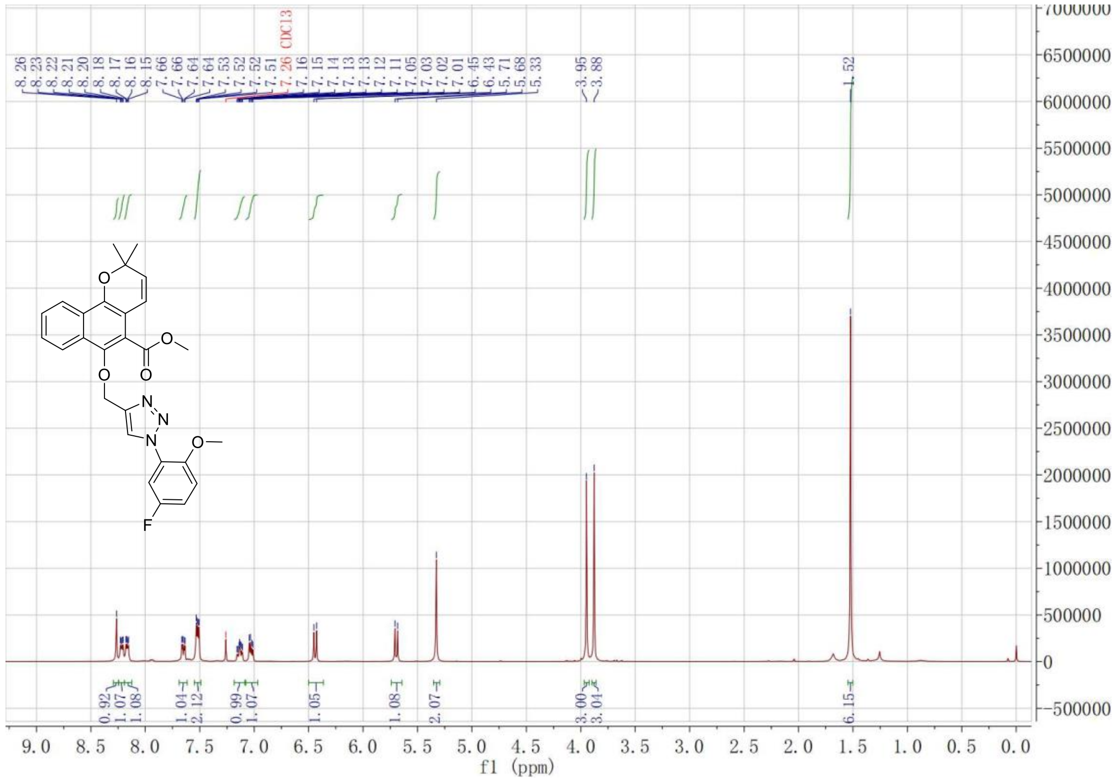

Figure 5-1. ^1H NMR spectrum of compound **9**

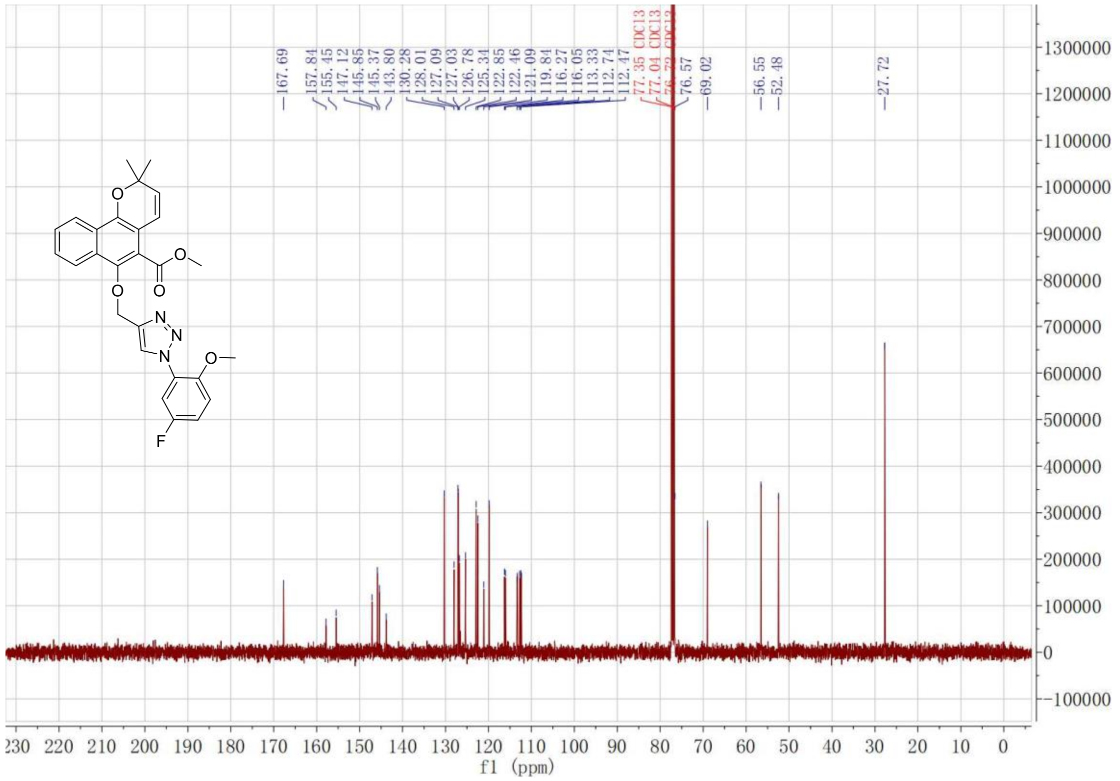

Figure 5-2. ^{13}C NMR spectrum of compound **9**

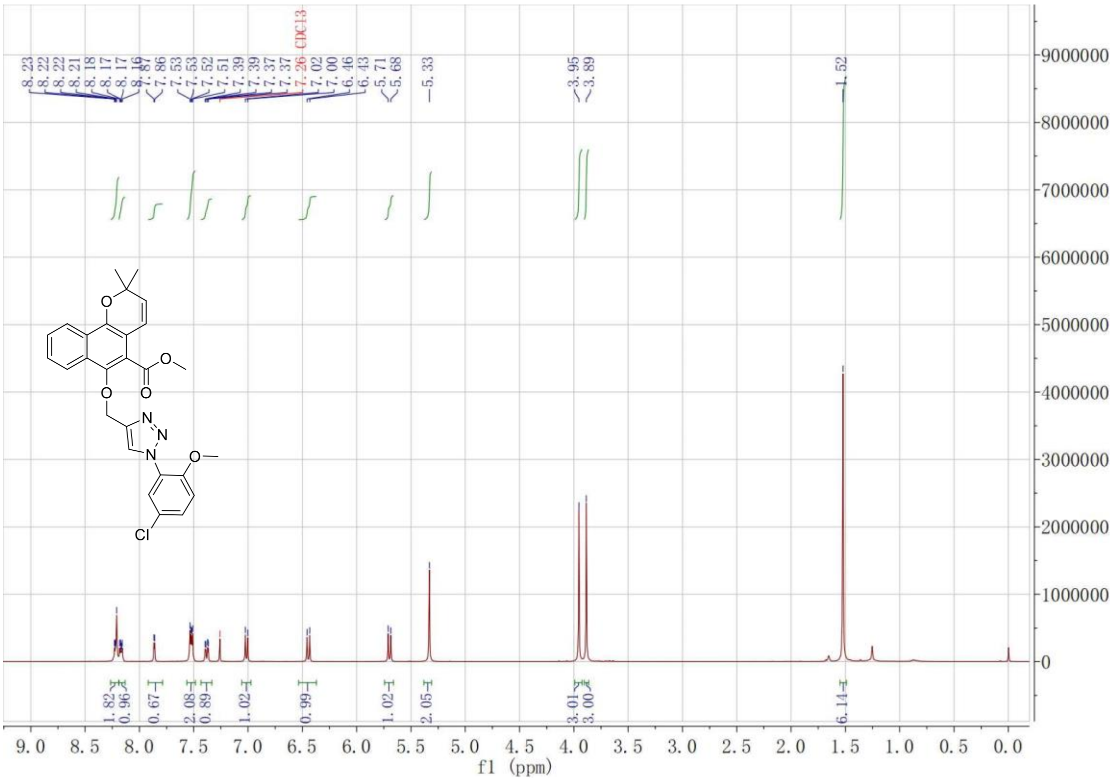

Figure 6-1. ^{1}H NMR spectrum of compound **10**

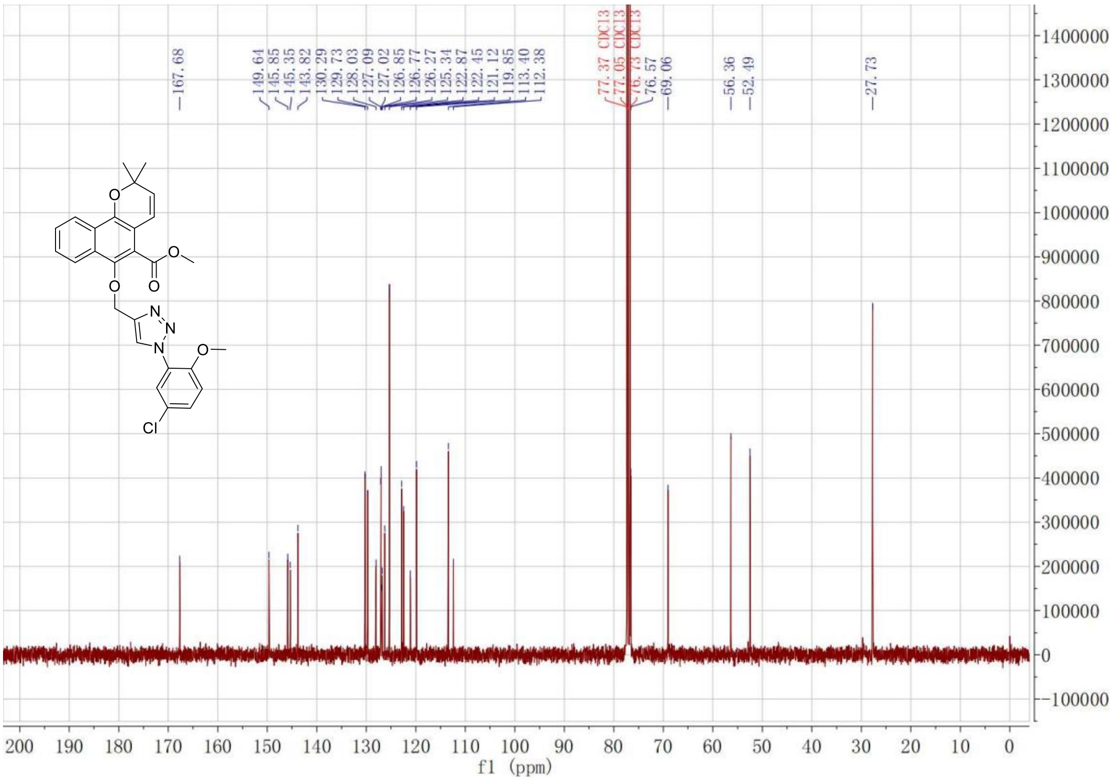

Figure 6-2. ^{13}C NMR spectrum of compound **10**

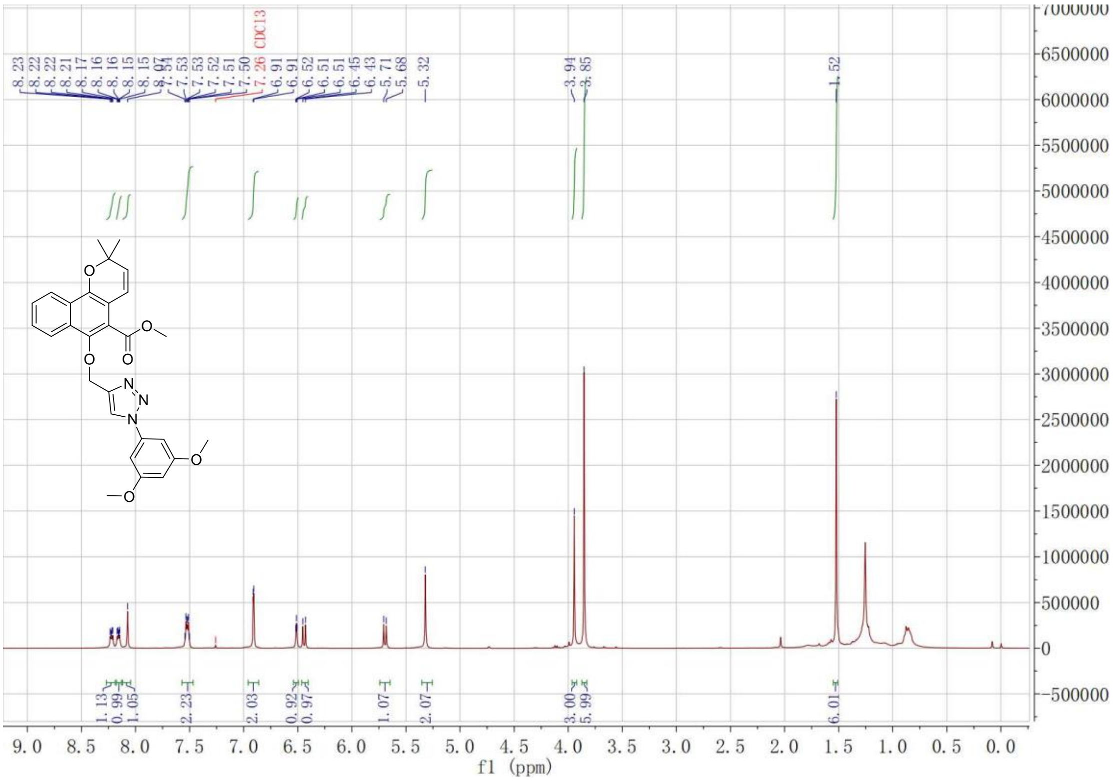

Figure 7-1. ^{1}H NMR spectrum of compound **11**

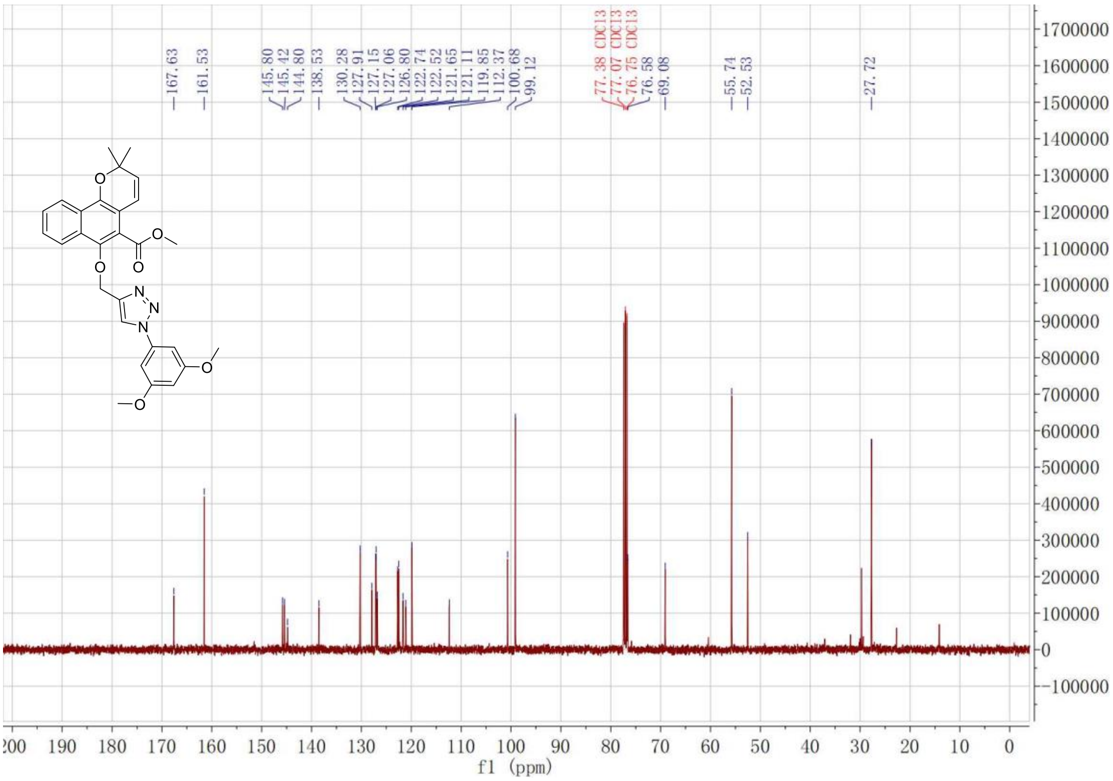

Figure 7-2. ^{13}C NMR spectrum of compound **11**

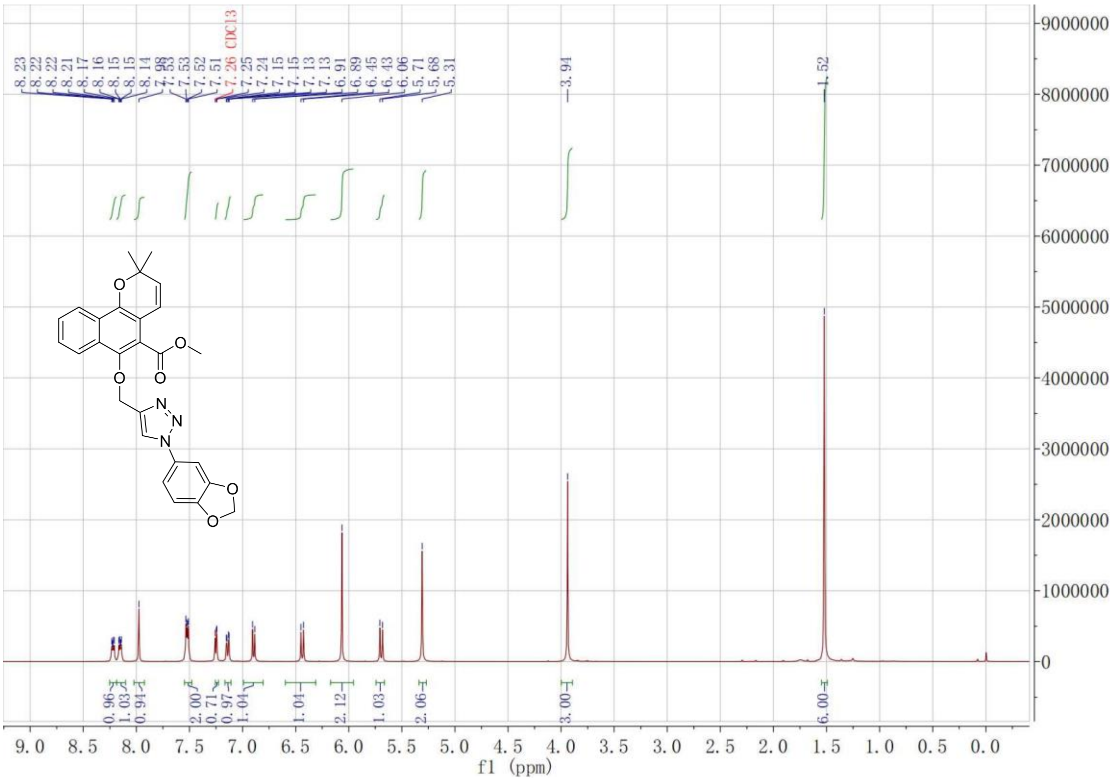

Figure 8-1. ^{1}H NMR spectrum of compound **12**

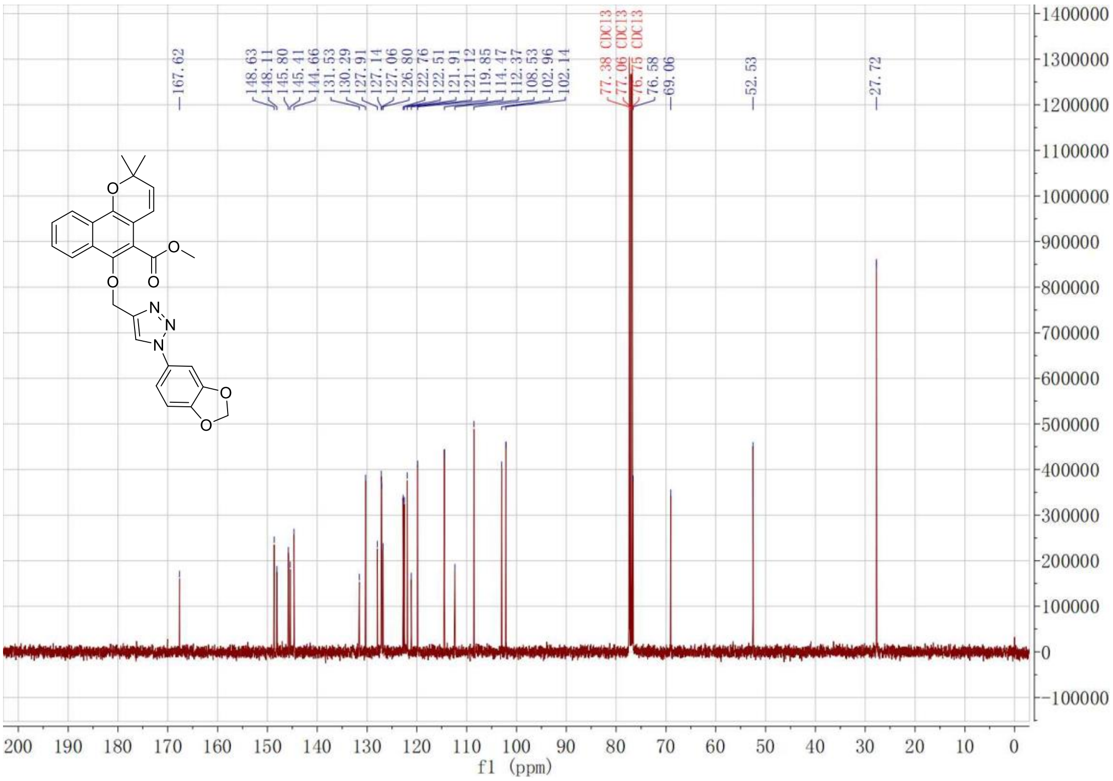

Figure 8-2. ^{13}C NMR spectrum of compound **12**

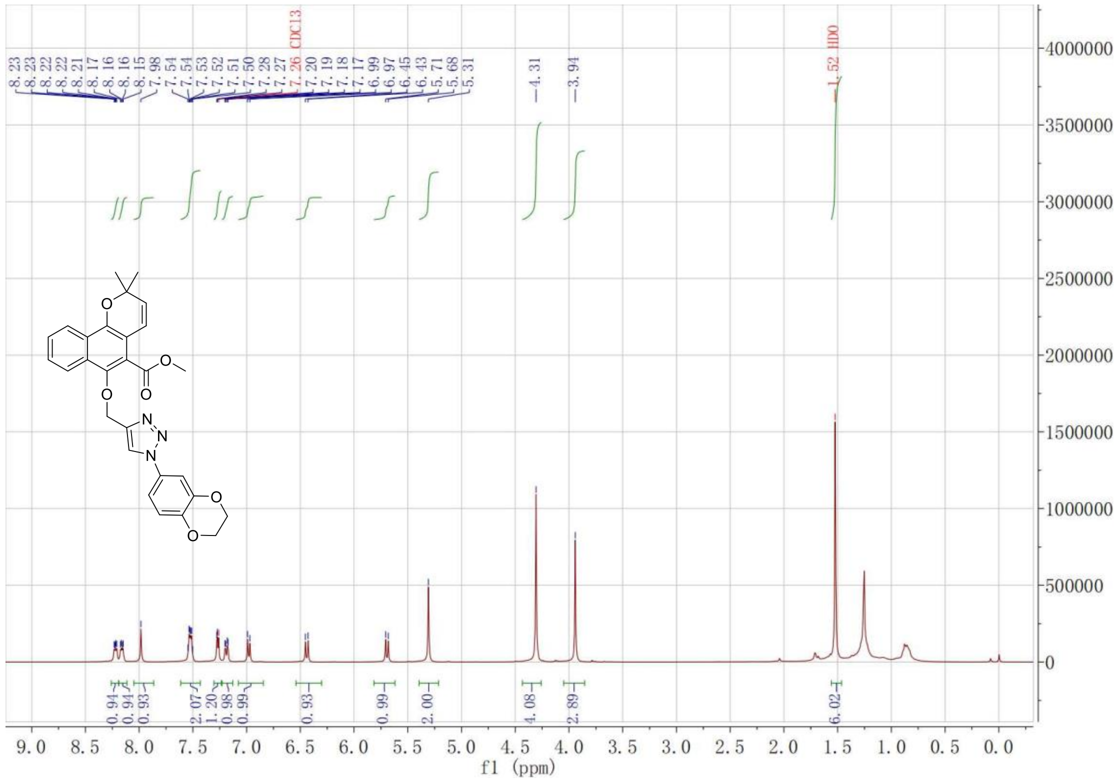

Figure 9-1. ^{1}H NMR spectrum of compound **13**

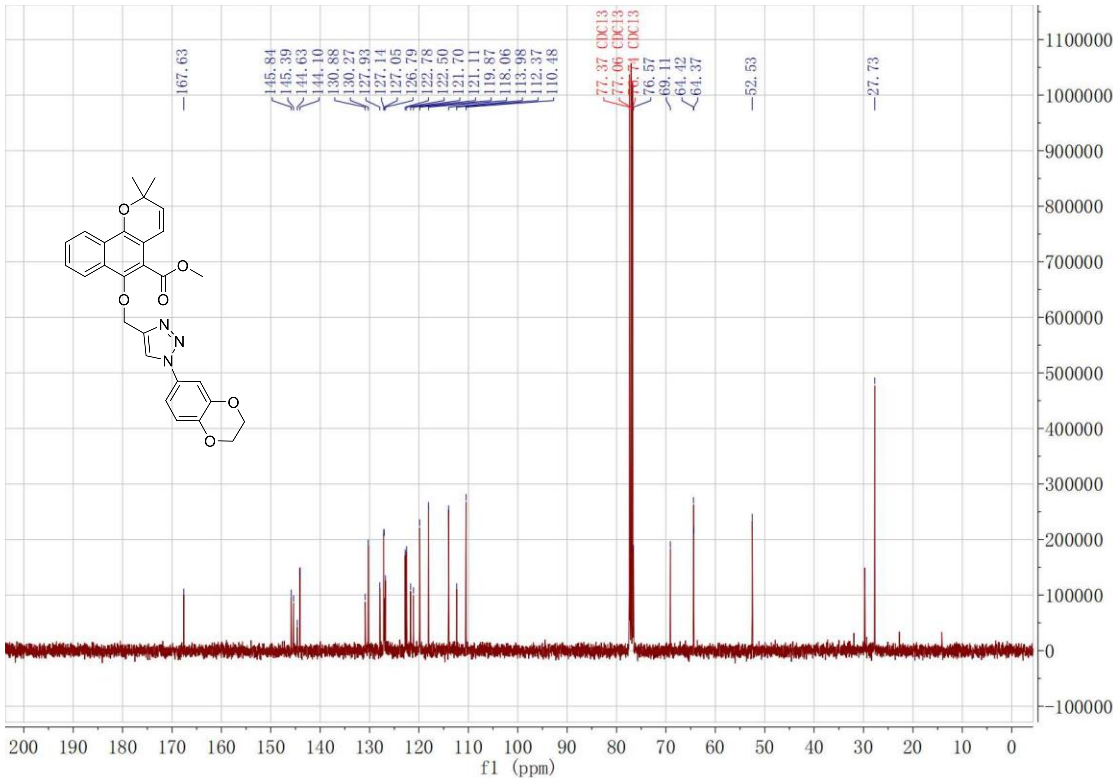

Figure 9-2. ^{13}C NMR spectrum of compound **13**

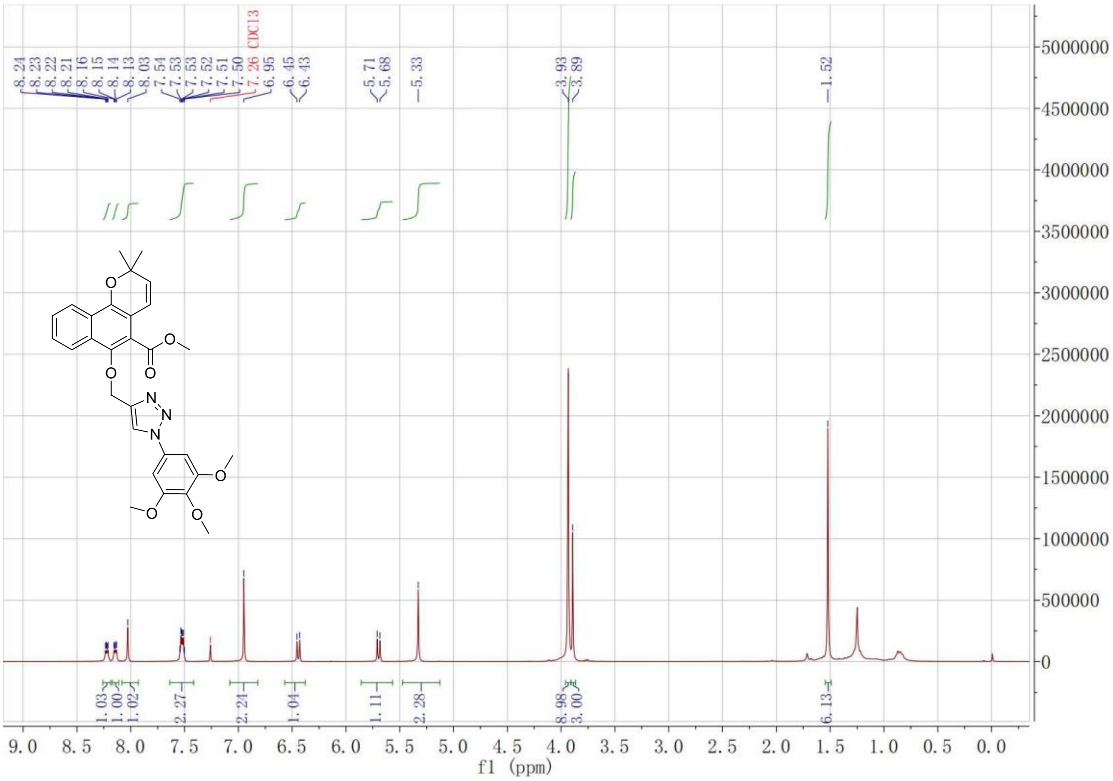

Figure 10-1. ^{1}H NMR spectrum of compound **14**

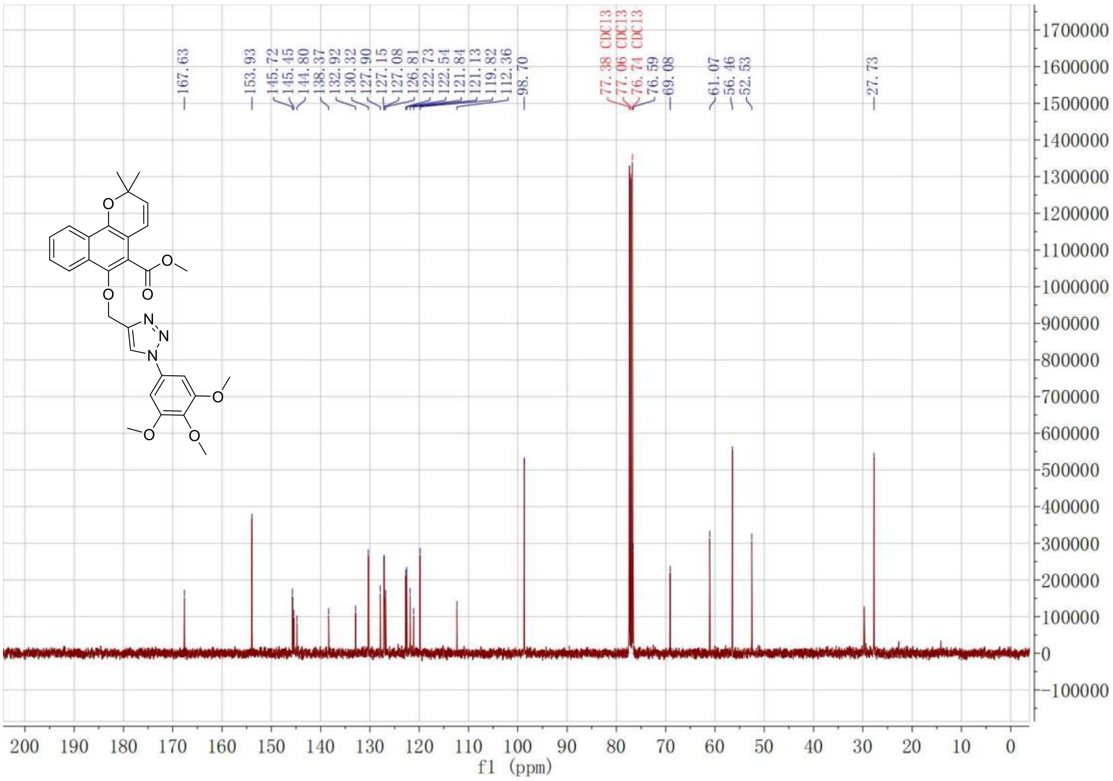

Figure 10-2. ^{13}C NMR spectrum of compound **14**

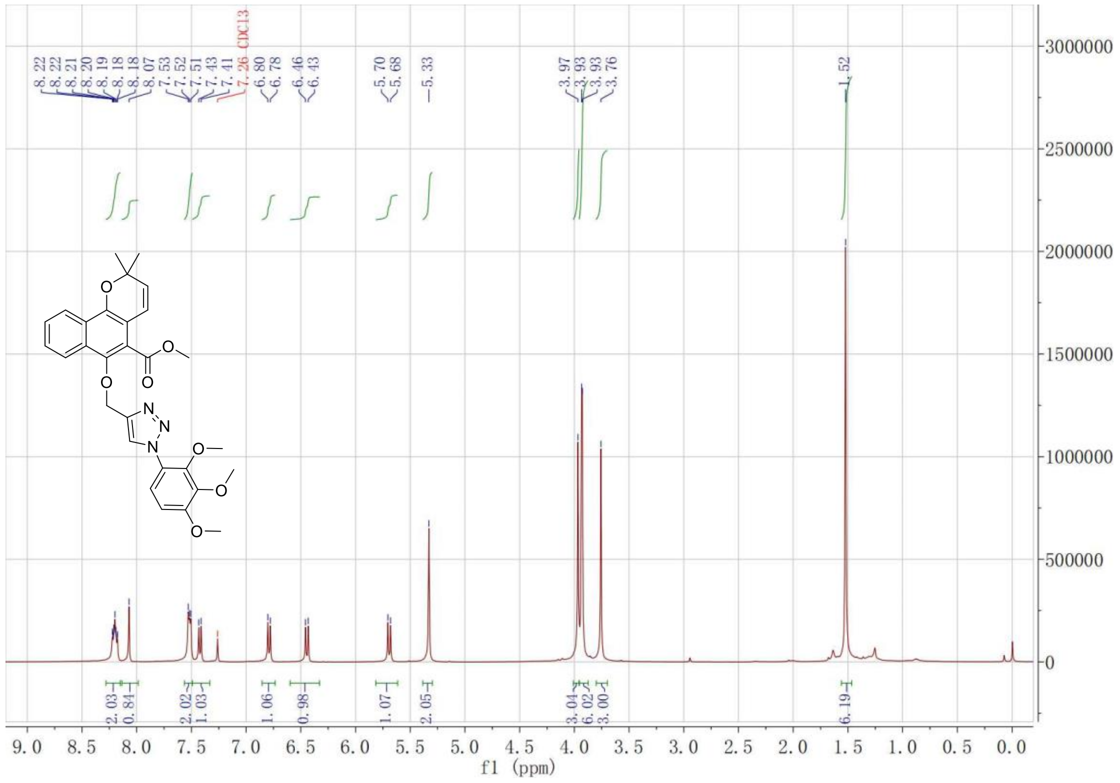

Figure 11-1. ^{1}H NMR spectrum of compound **15**

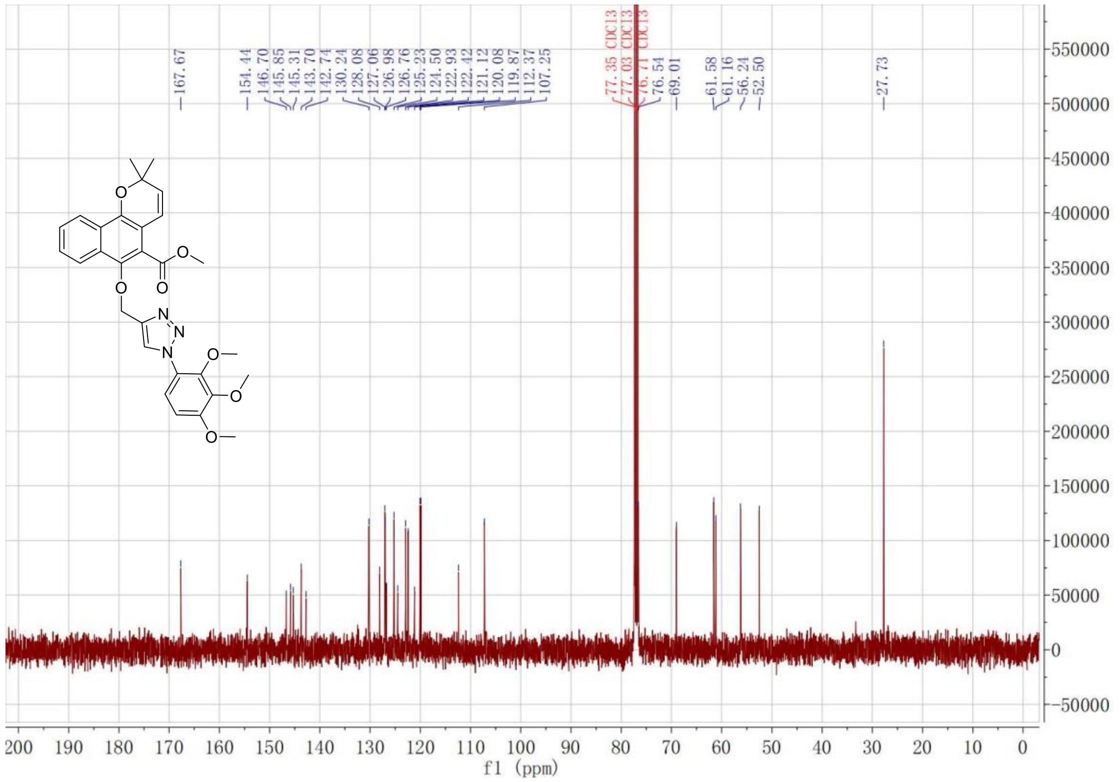

Figure 11-2. ^{13}C NMR spectrum of compound **15**

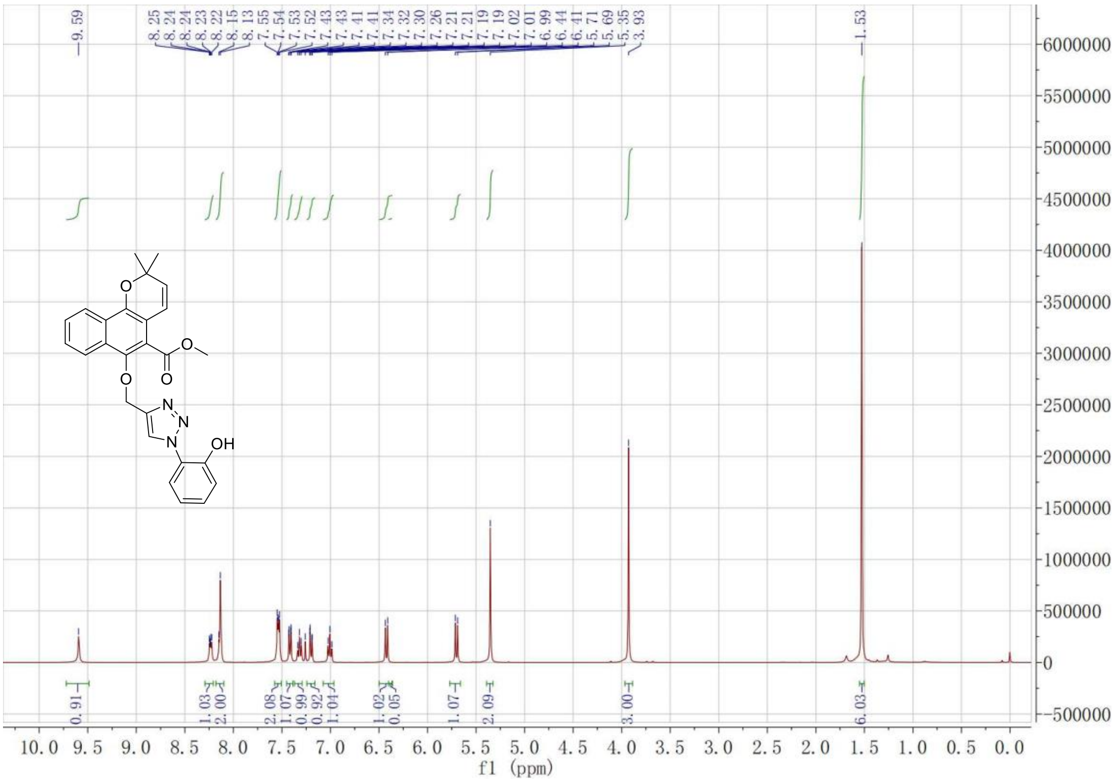

Figure 12-1. ^{1}H NMR spectrum of compound **16**

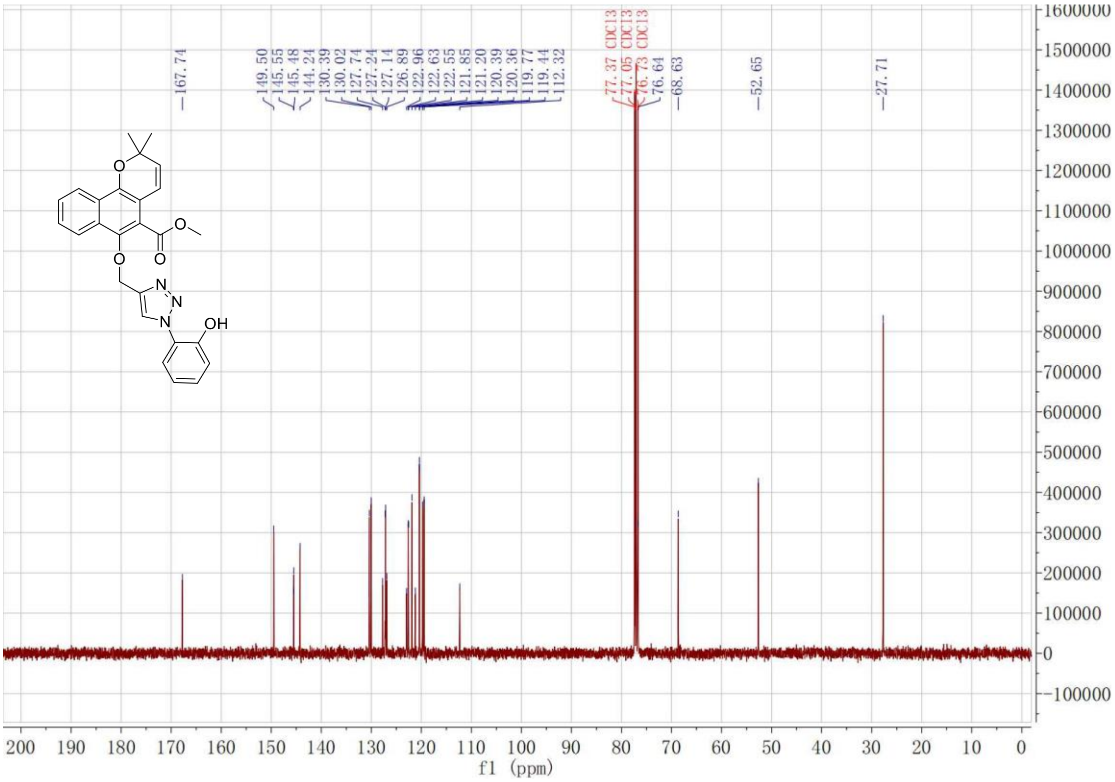

Figure 12-2. ^{13}C NMR spectrum of compound **16**

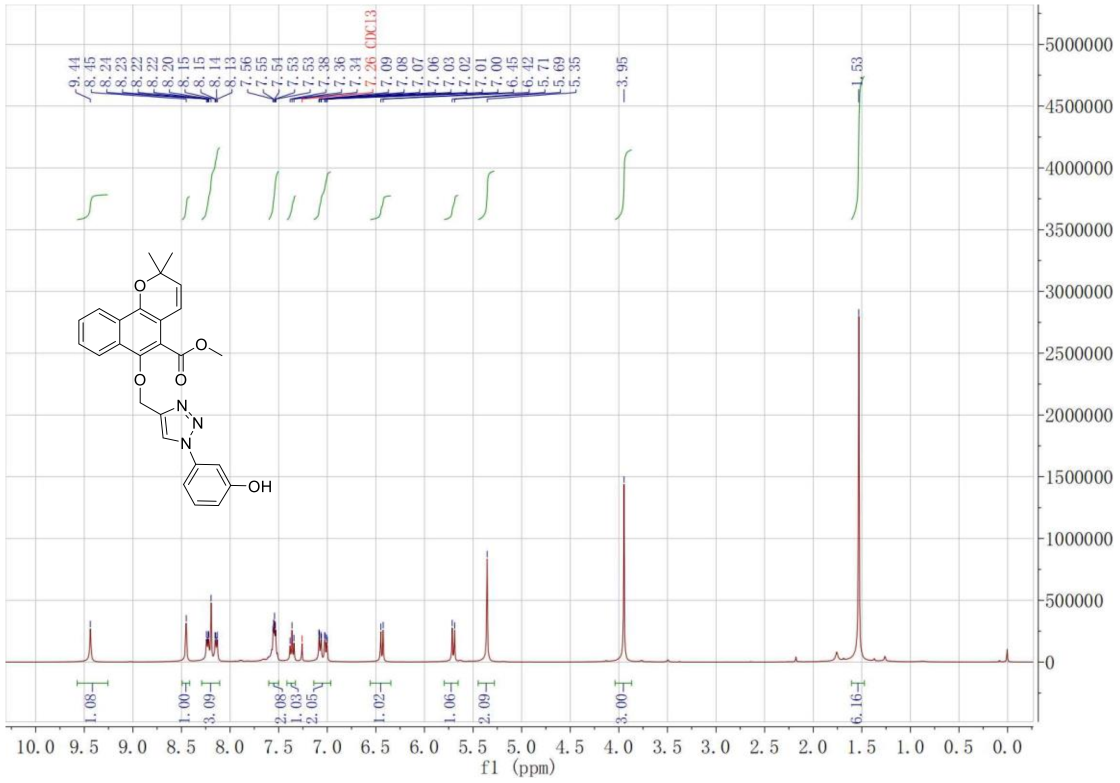

Figure 13-1. ^{1}H NMR spectrum of compound **17**

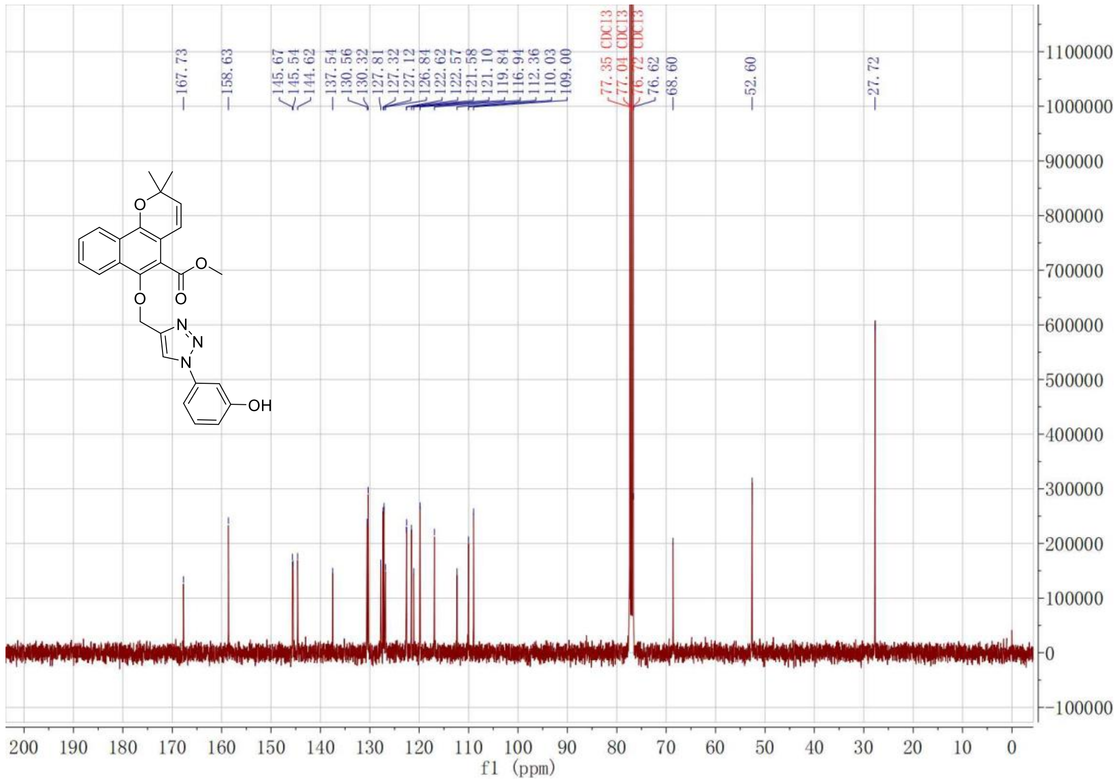

Figure 13-2. ^{13}C NMR spectrum of compound **17**

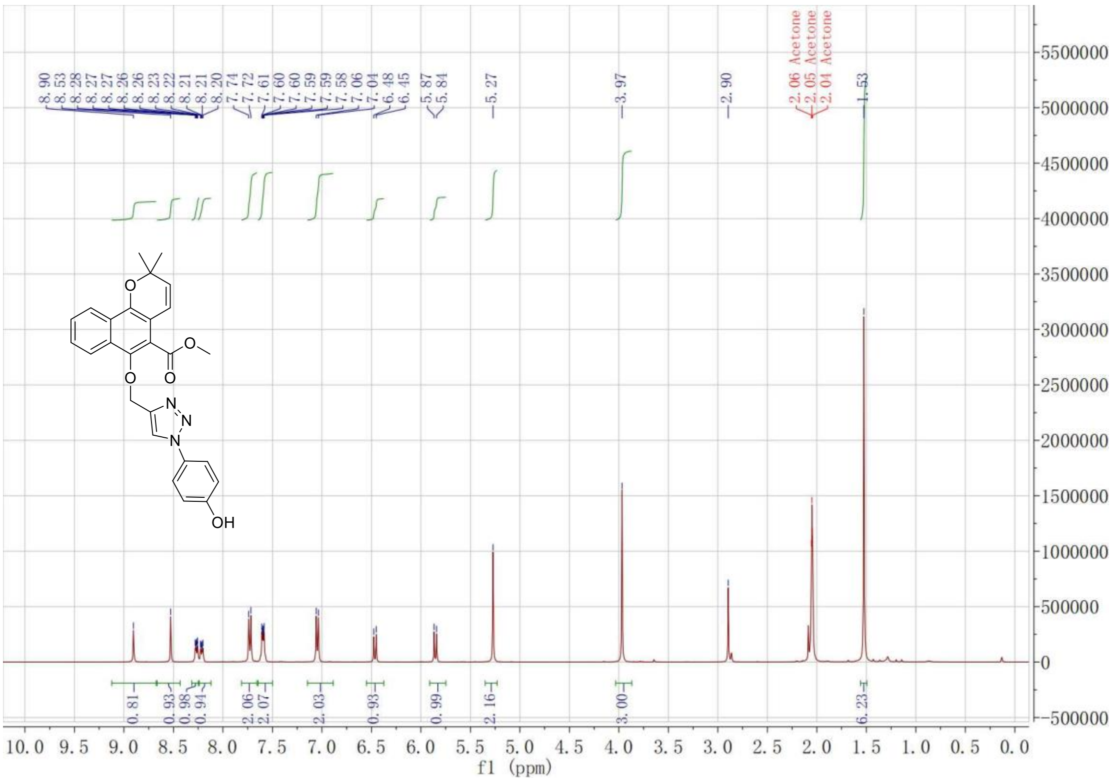

Figure 14-1. ^{1}H NMR spectrum of compound **18**

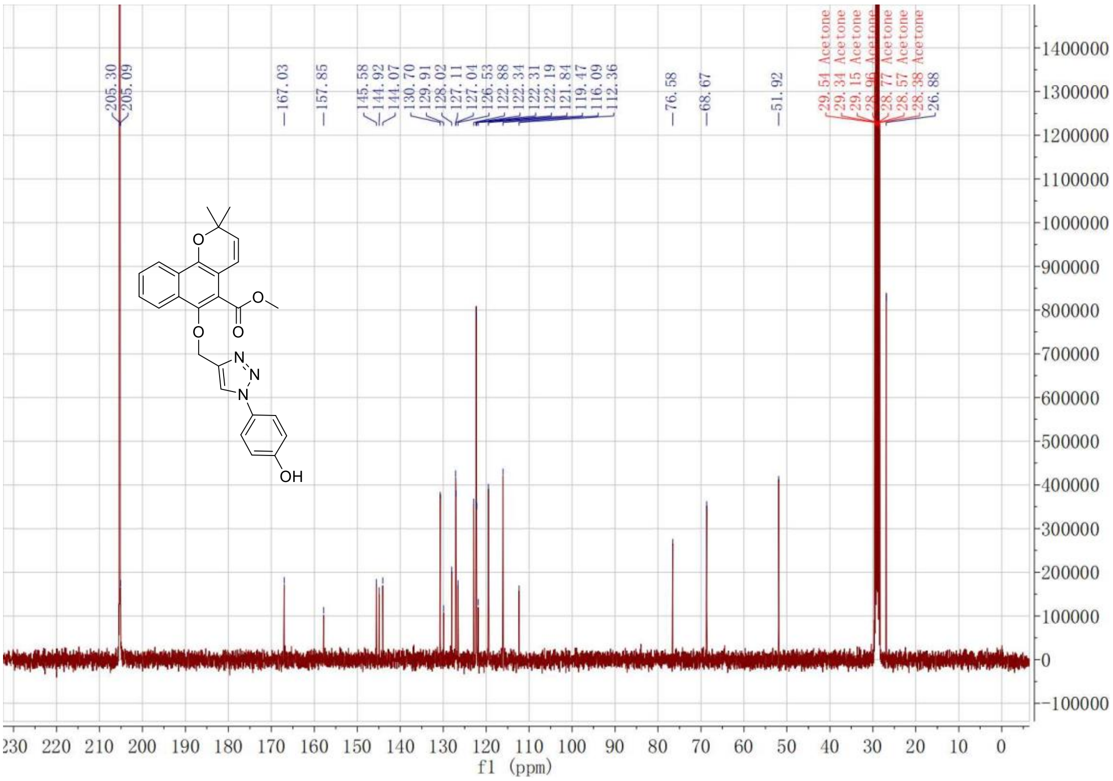

Figure 14-2. ^{13}C NMR spectrum of compound **18**

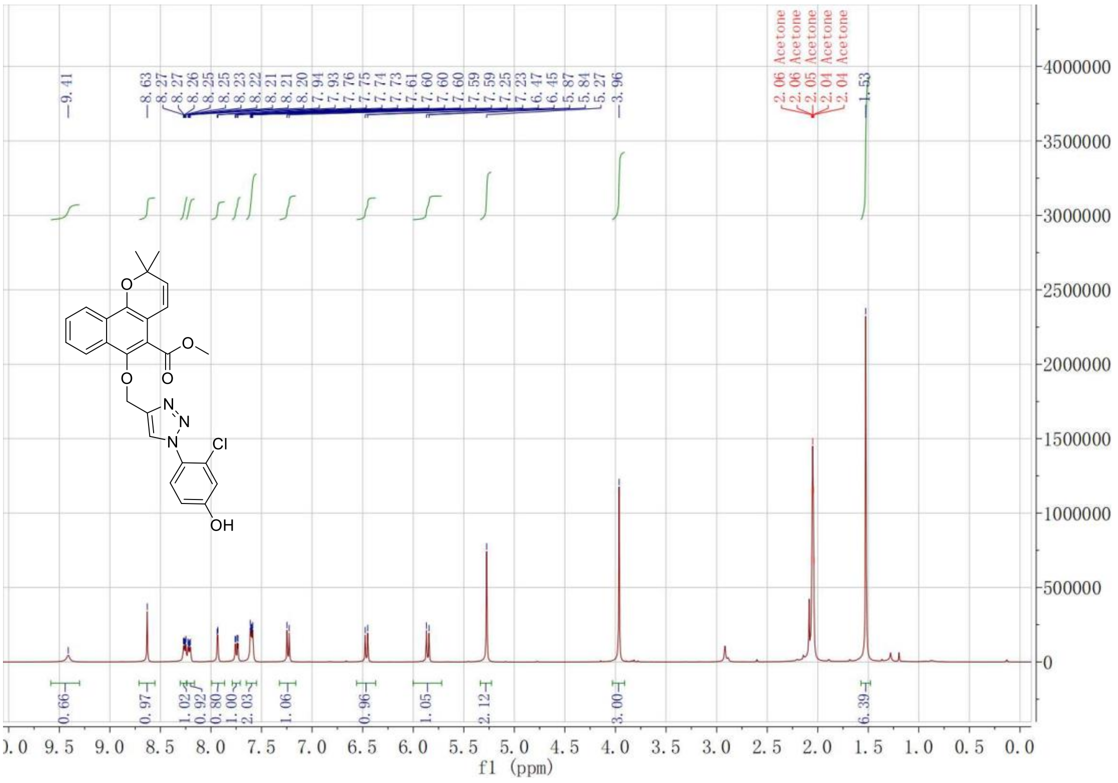

Figure 15-1. ^{1}H NMR spectrum of compound **19**

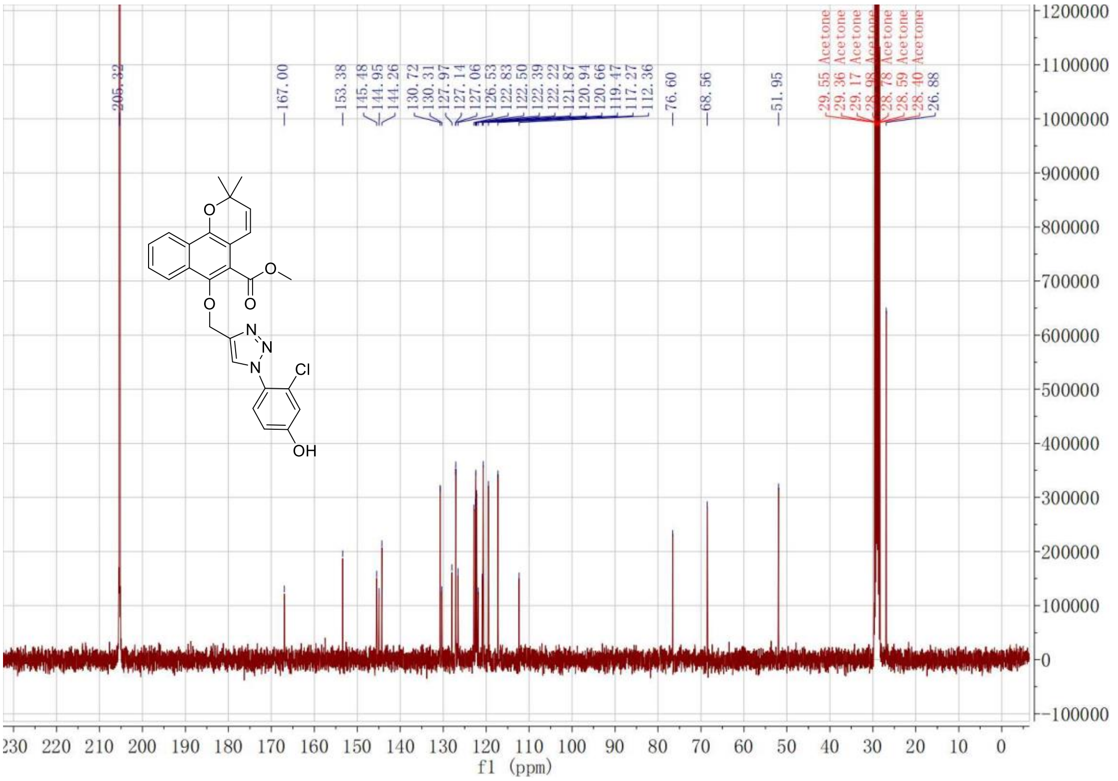

Figure 15-2. ^{13}C NMR spectrum of compound **19**

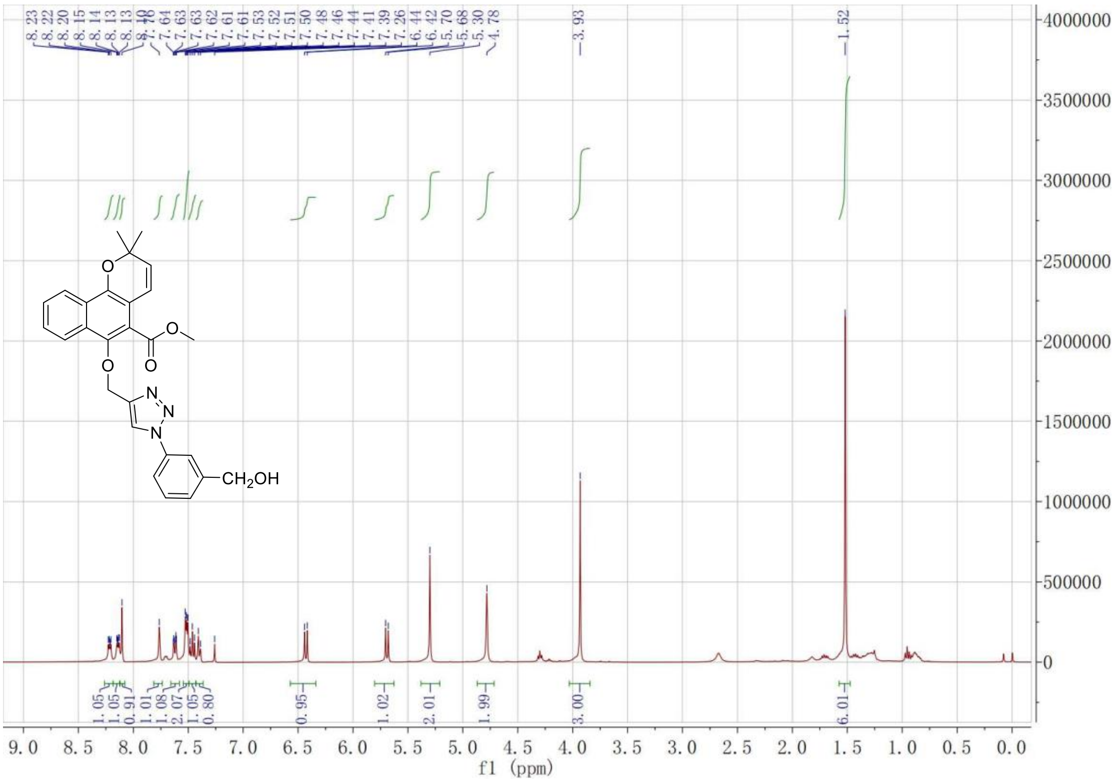

Figure 16-1. ^{1}H NMR spectrum of compound **20**

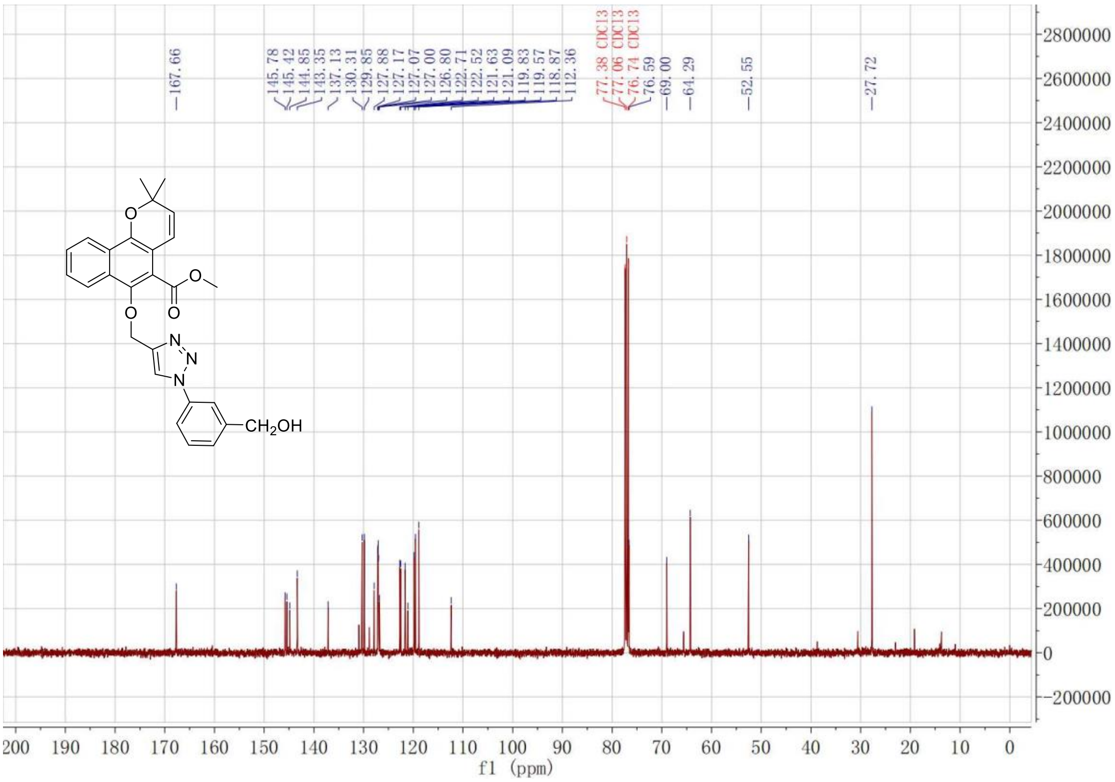

Figure 16-2. ^{13}C NMR spectrum of compound **20**

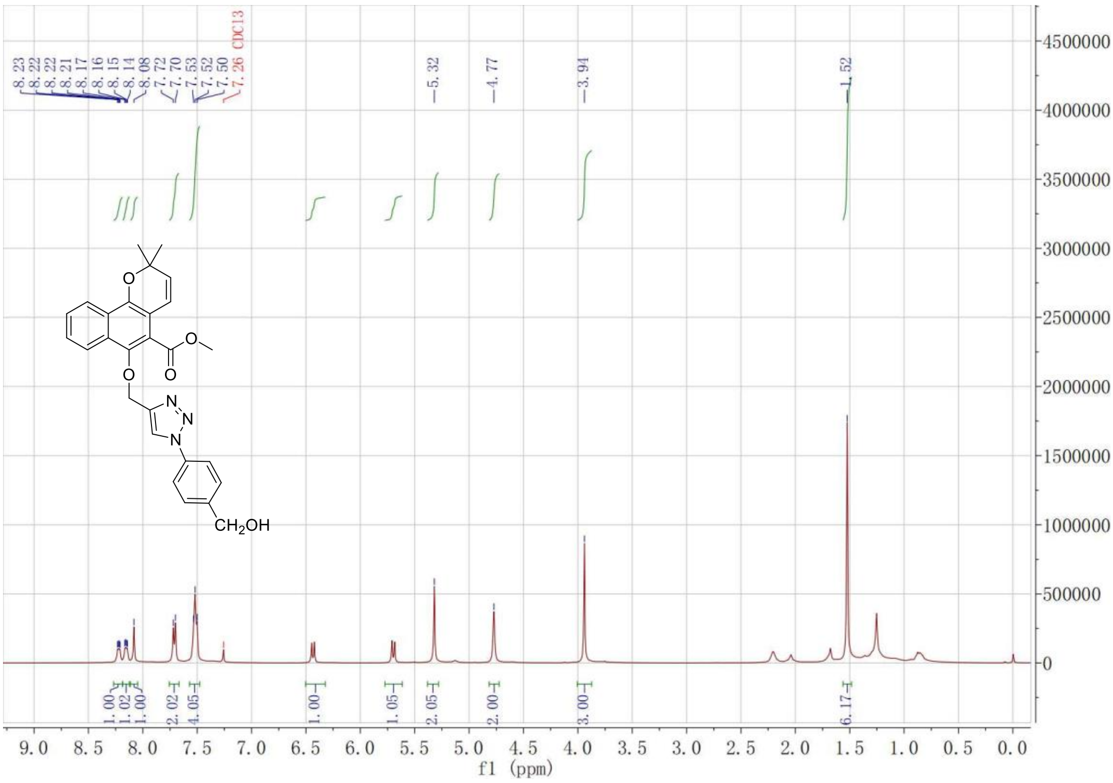

Figure 17-1. ^{1}H NMR spectrum of compound **21**

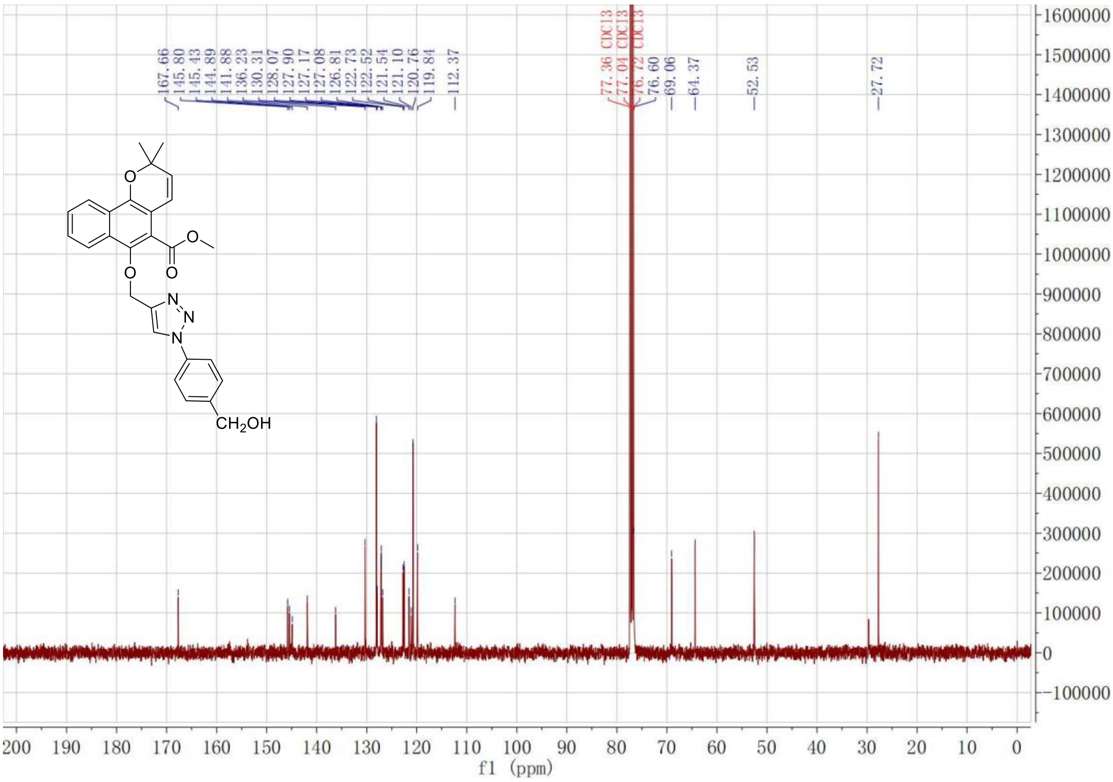

Figure 17-2. ^{13}C NMR spectrum of compound **21**

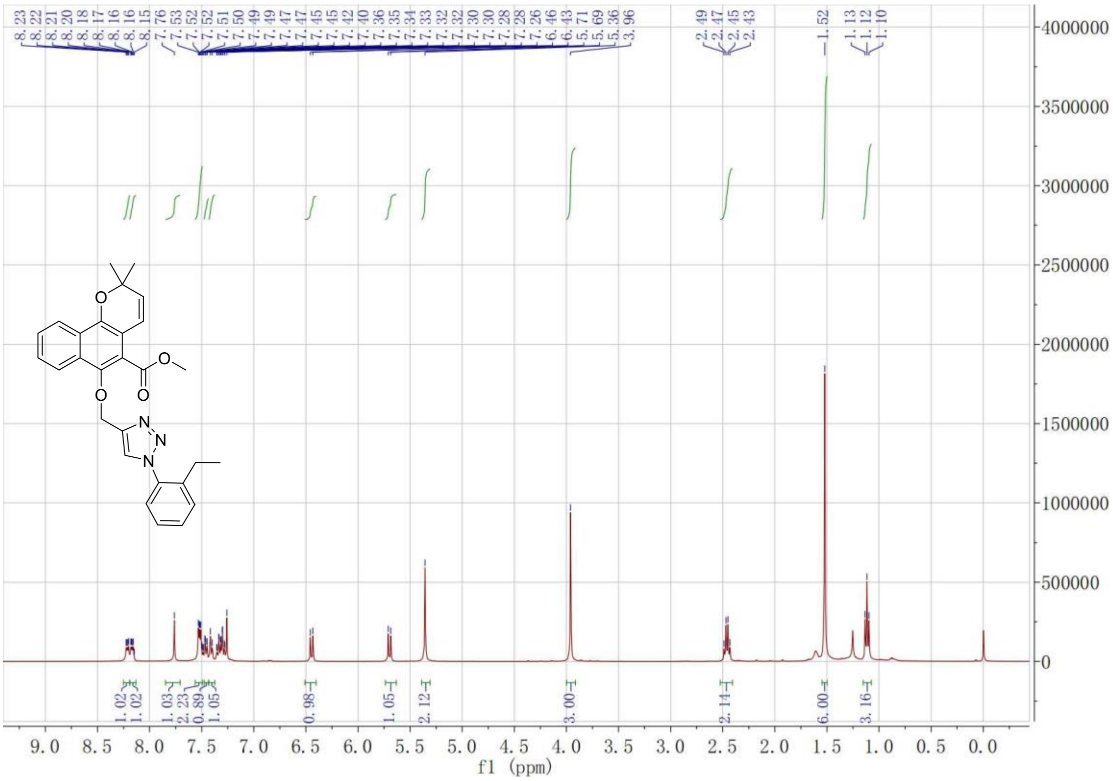

Figure 18-1. ^{1}H NMR spectrum of compound **22**

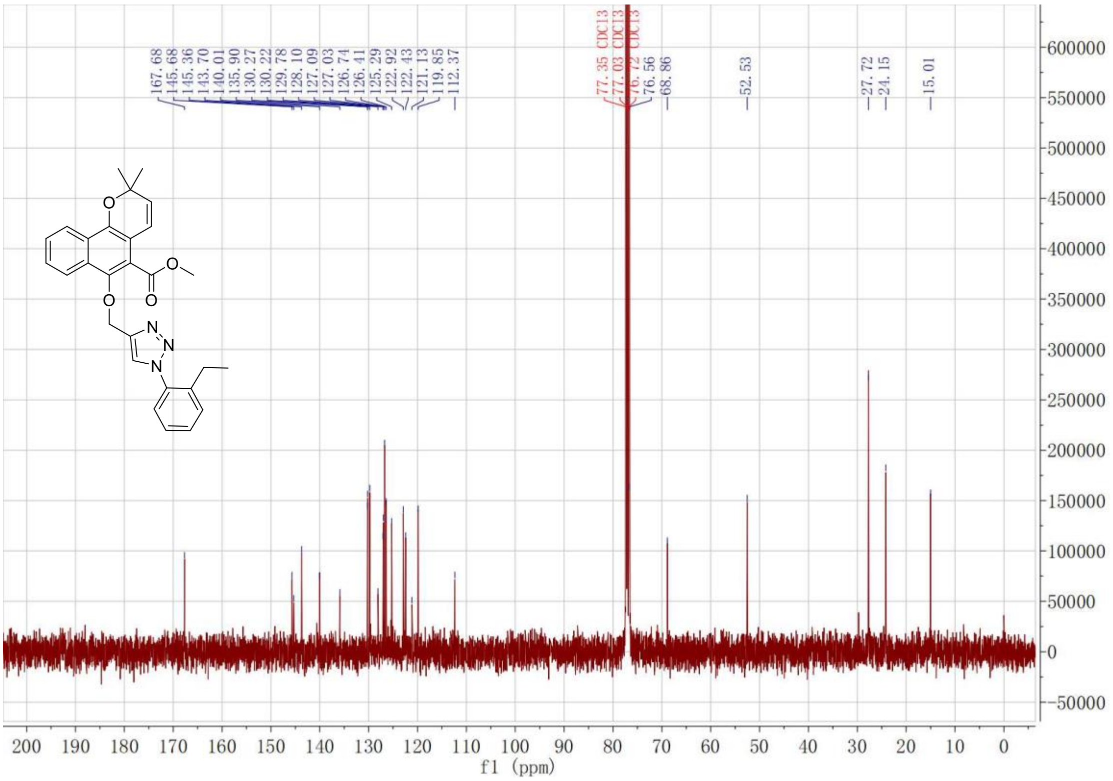

Figure 18-2. ^{13}C NMR spectrum of compound **22**

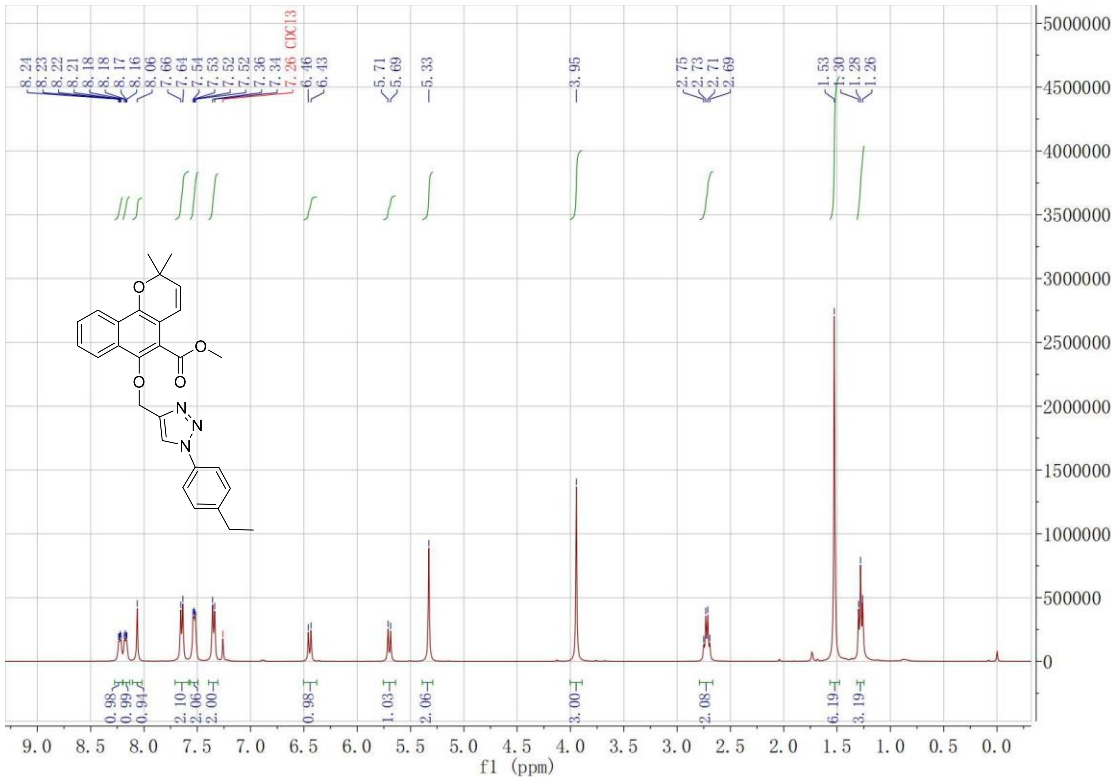

Figure 19-1. ^{1}H NMR spectrum of compound **23**

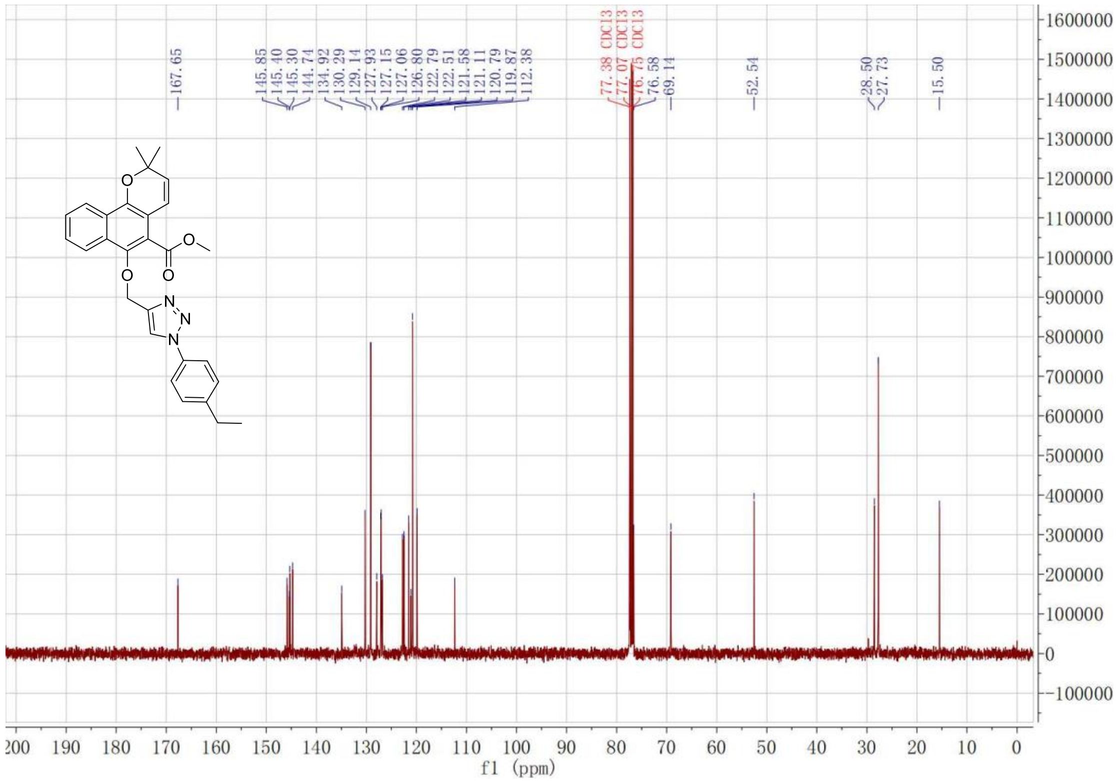

Figure 19-2. ^{13}C NMR spectrum of compound **23**

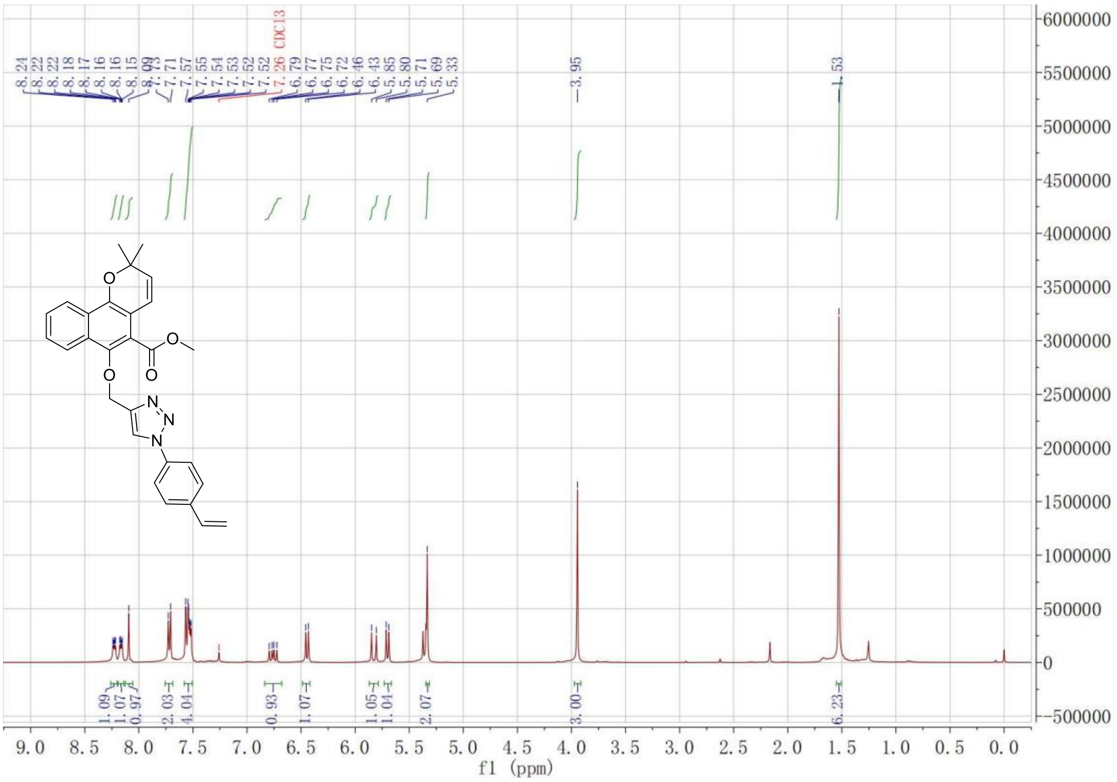

Figure 20-1. ^{1}H NMR spectrum of compound **24**

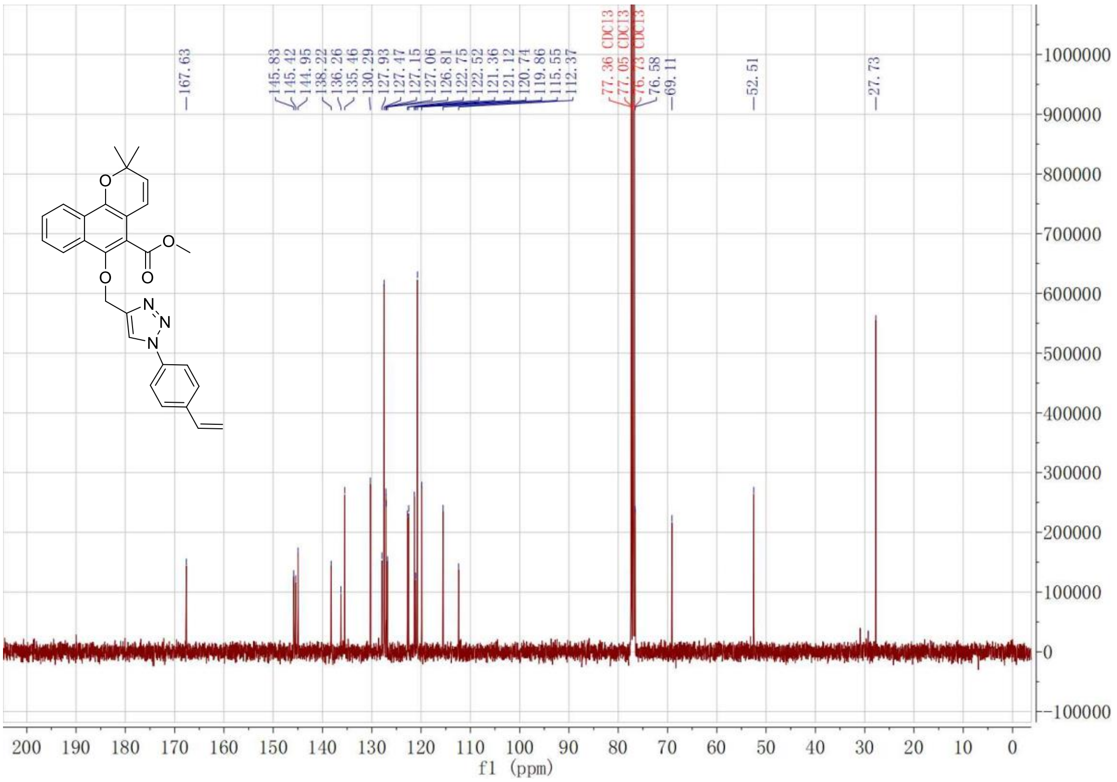

Figure 20-2. ^{13}C NMR spectrum of compound **24**

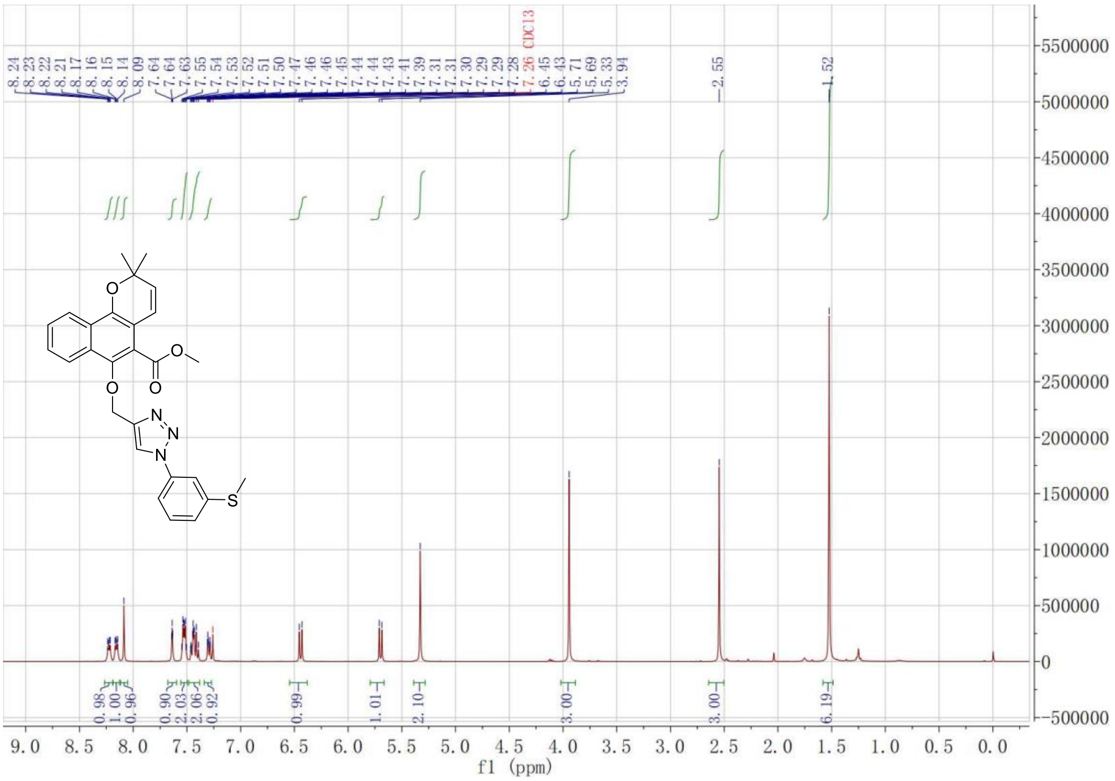

Figure 21-1. ^{1}H NMR spectrum of compound **25**

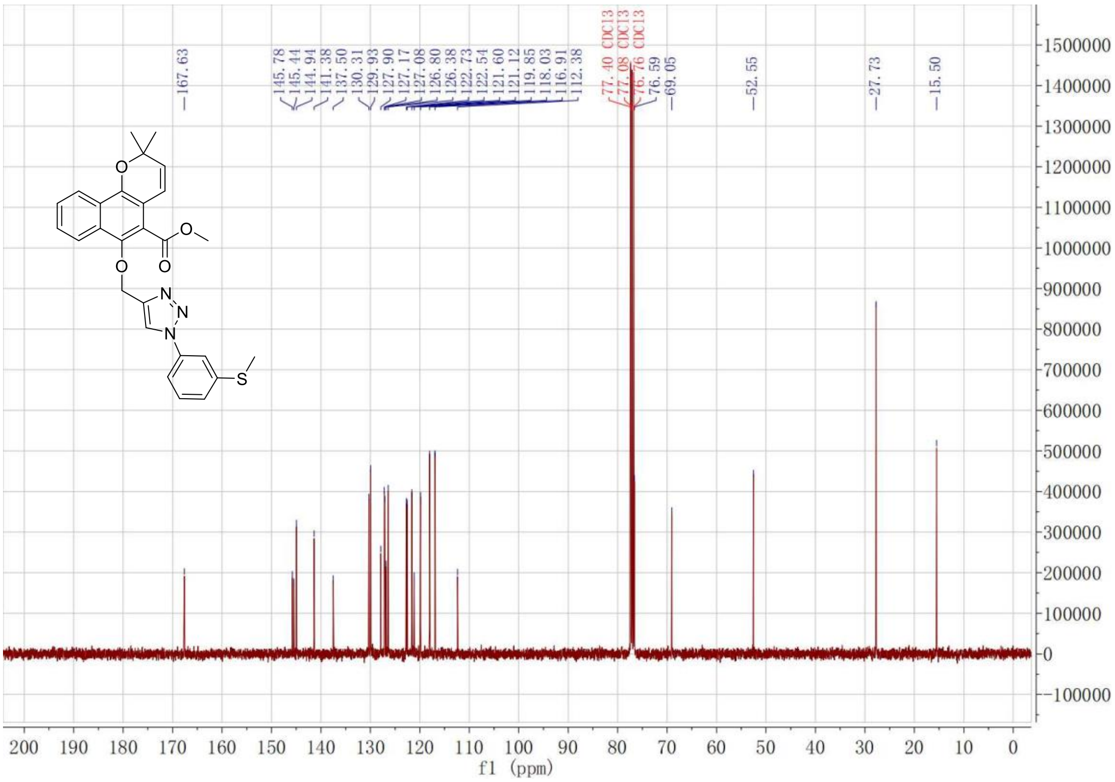

Figure 21-2. ^{13}C NMR spectrum of compound **25**

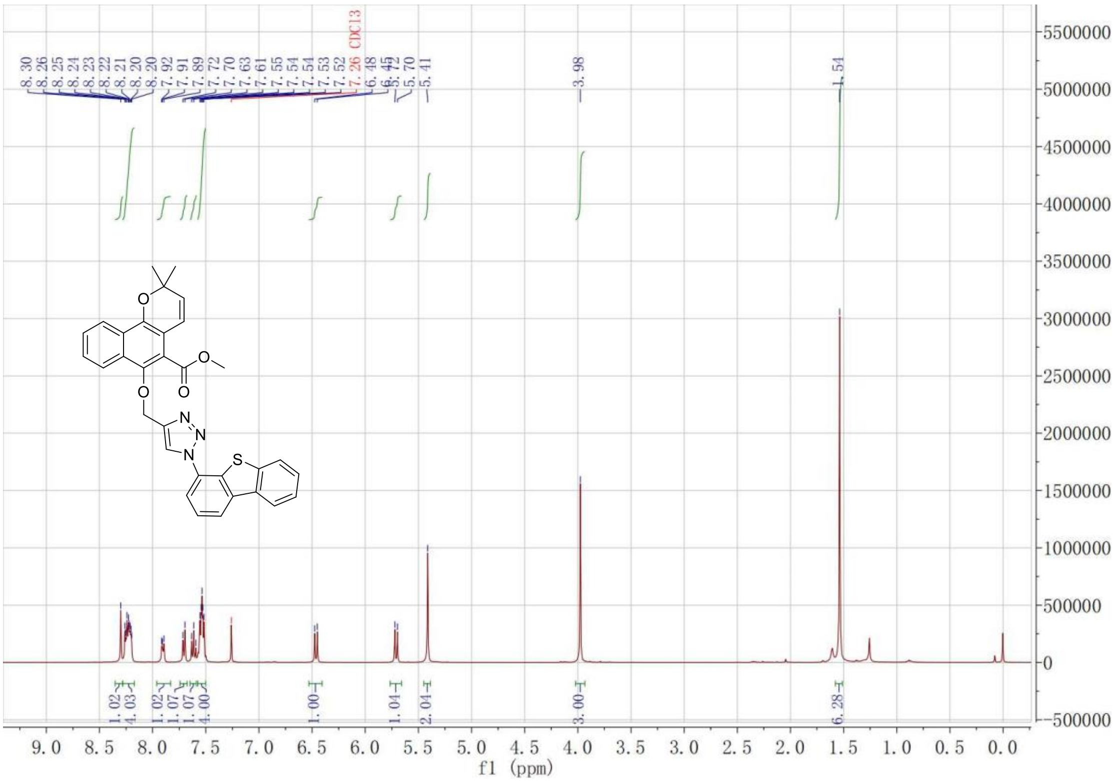

Figure 22-1. ^{1}H NMR spectrum of compound **26**

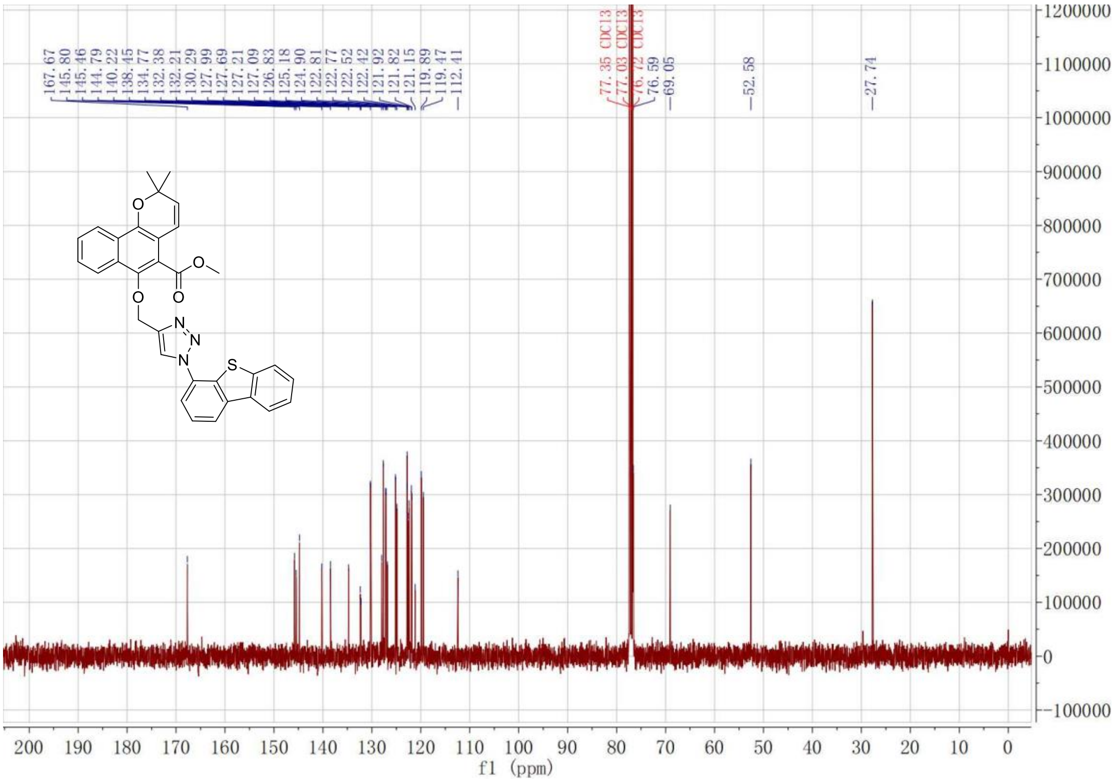

Figure 22-2. ^{13}C NMR spectrum of compound **26**

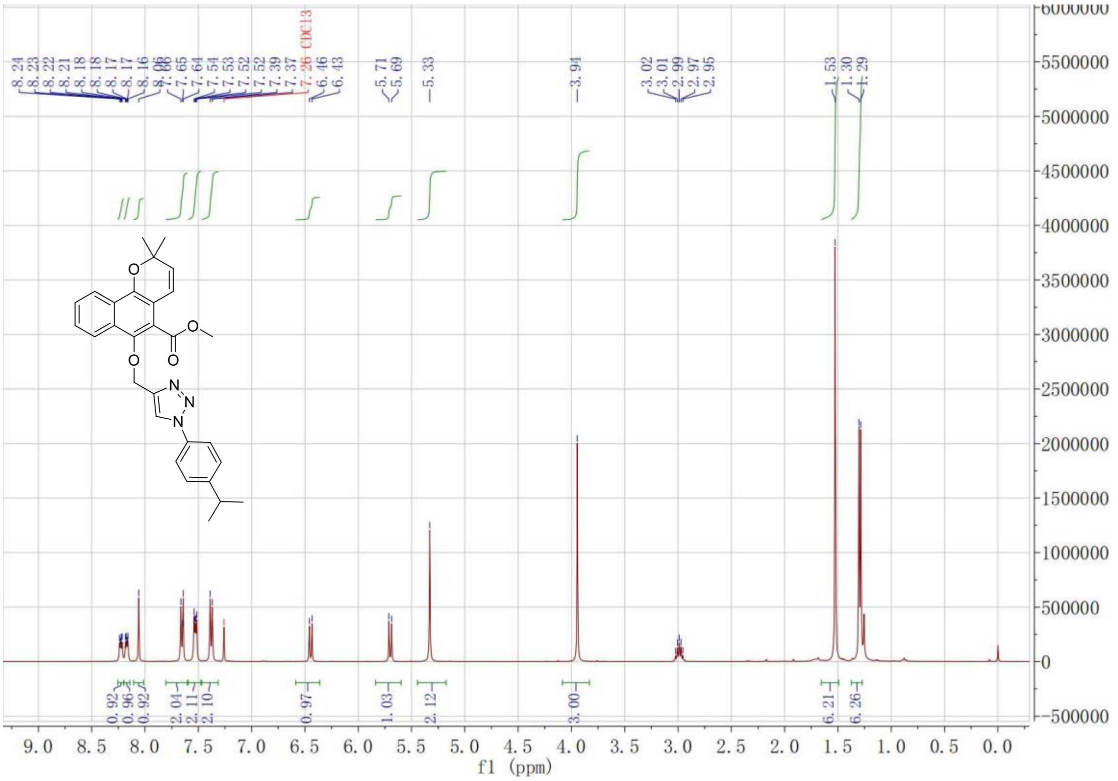

Figure 23-1. ^{1}H NMR spectrum of compound **27**

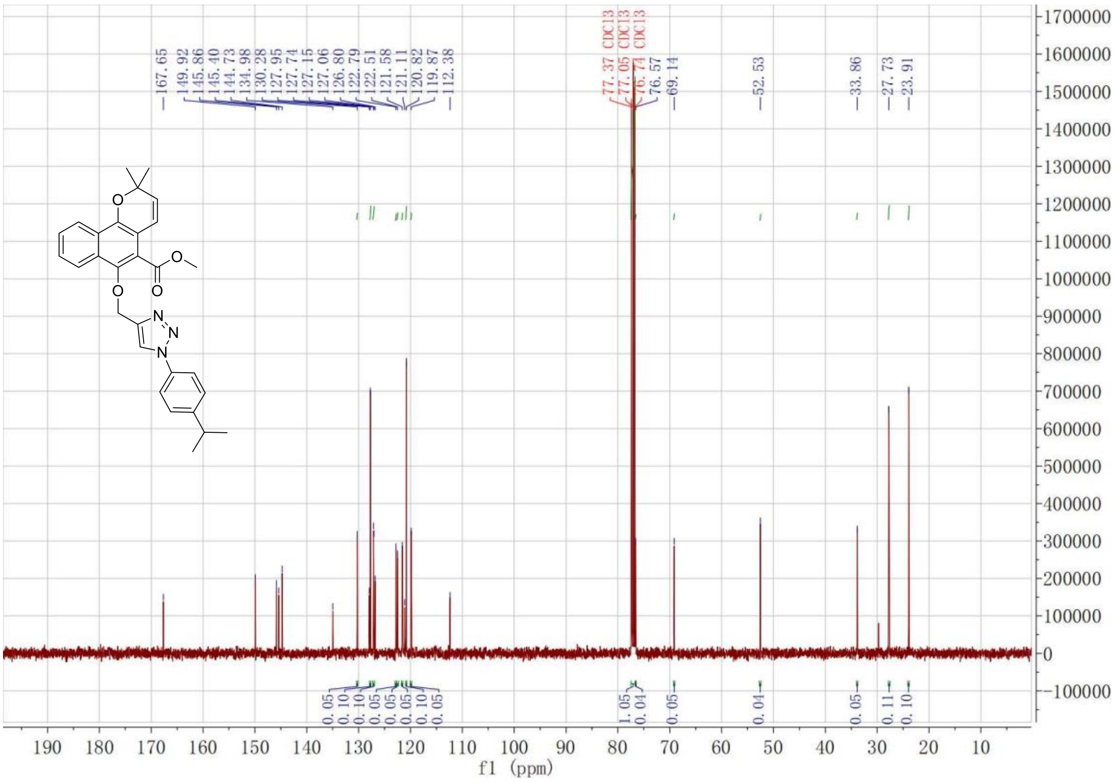

Figure 23-2. ^{13}C NMR spectrum of compound **27**

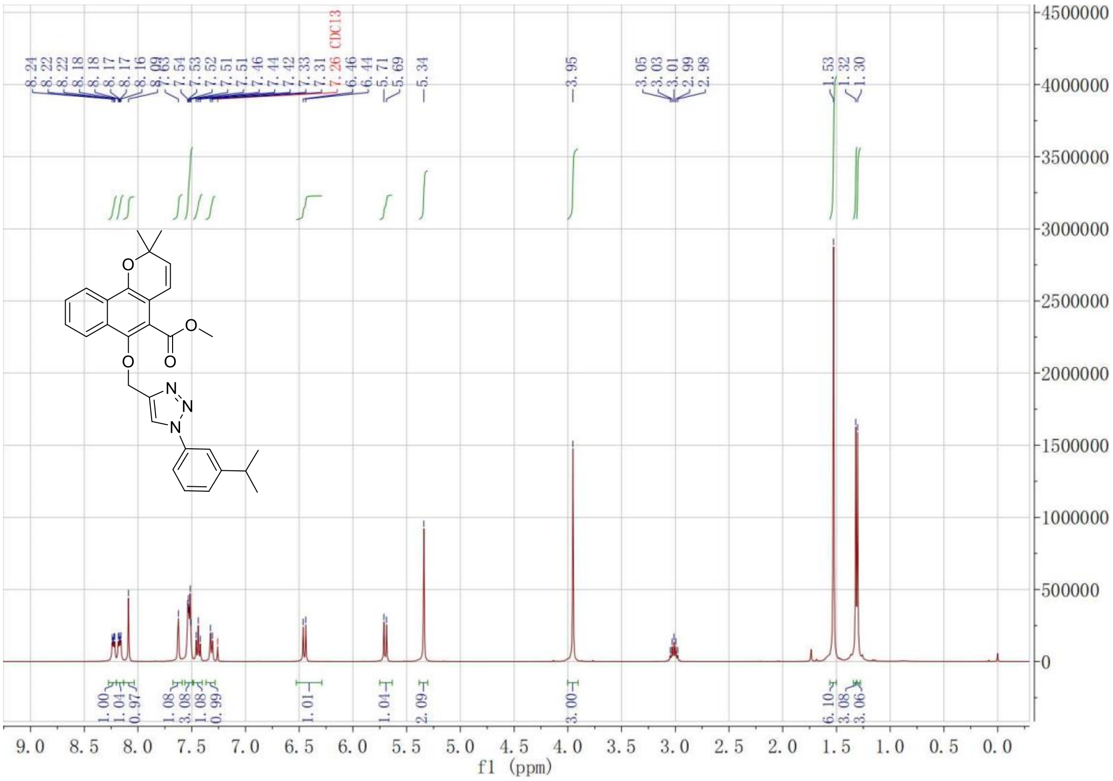

Figure 24-1. ^{1}H NMR spectrum of compound **28**

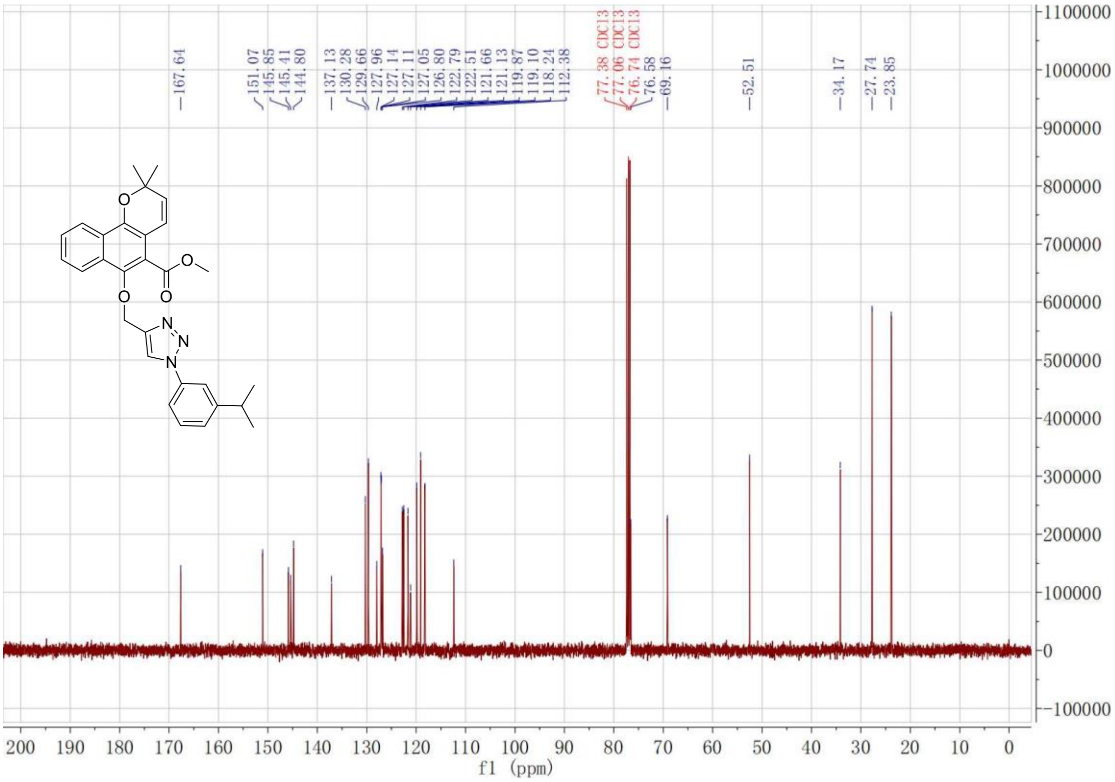

Figure 24-2. ^{13}C NMR spectrum of compound **28**

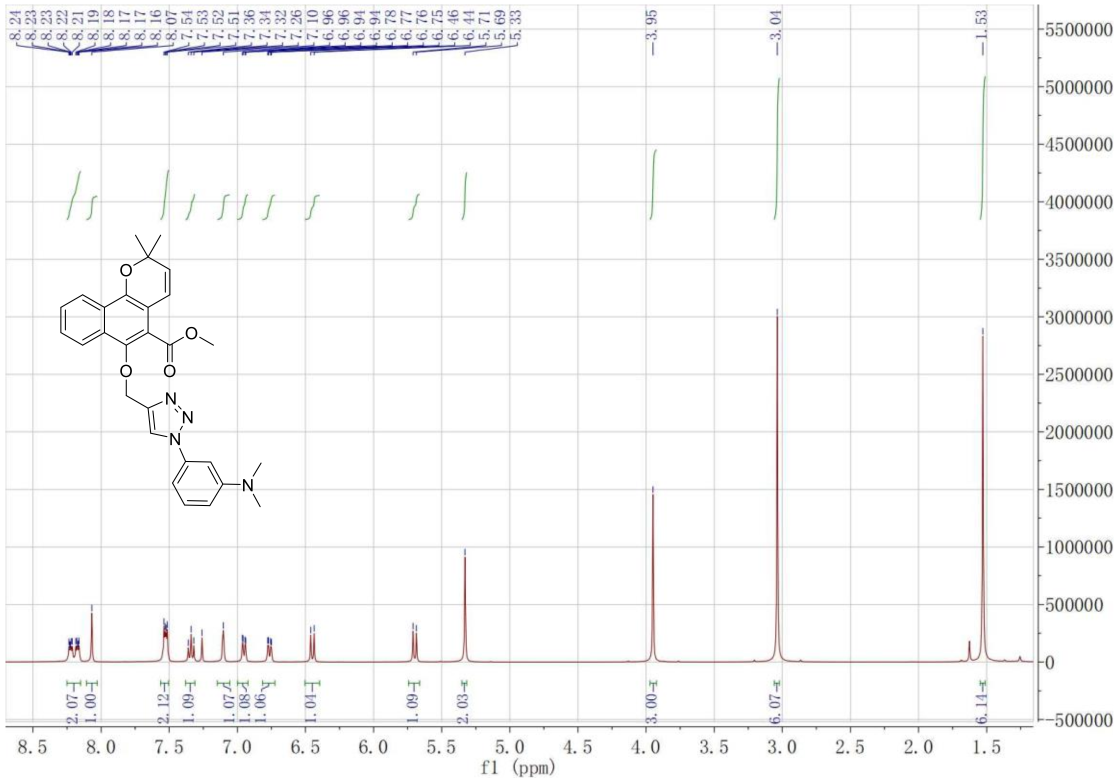

Figure 25-1. ^{1}H NMR spectrum of compound **29**

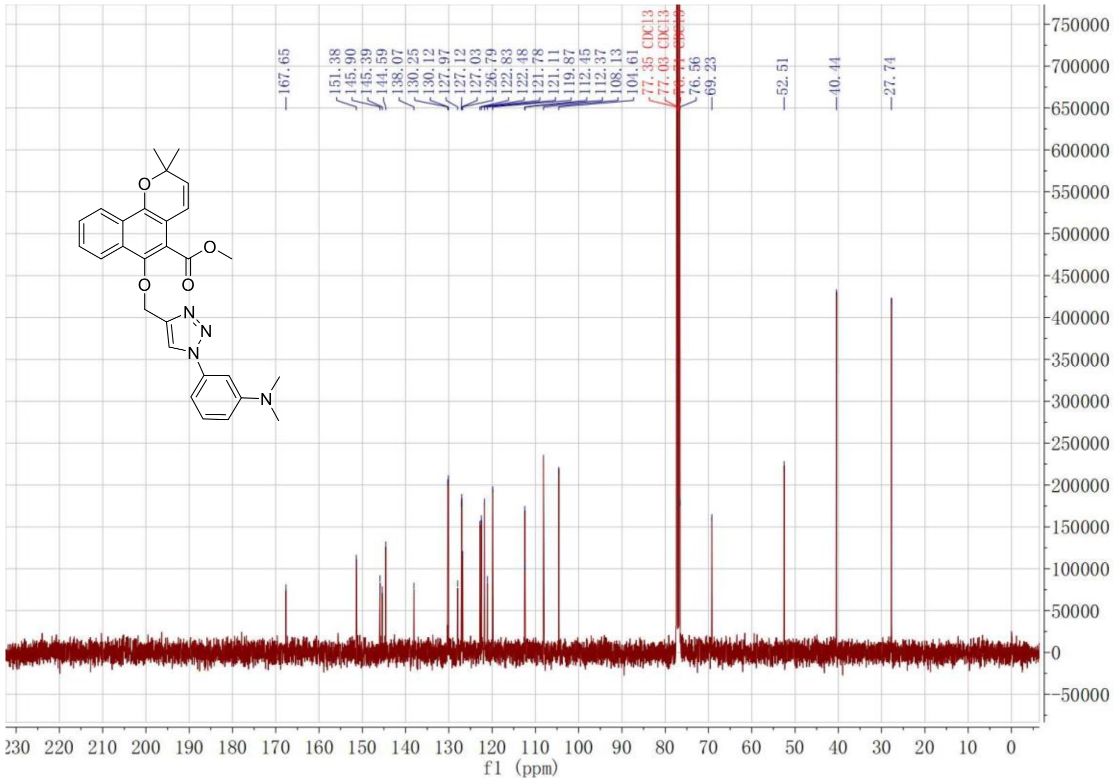

Figure 25-2. ^{13}C NMR spectrum of compound **29**

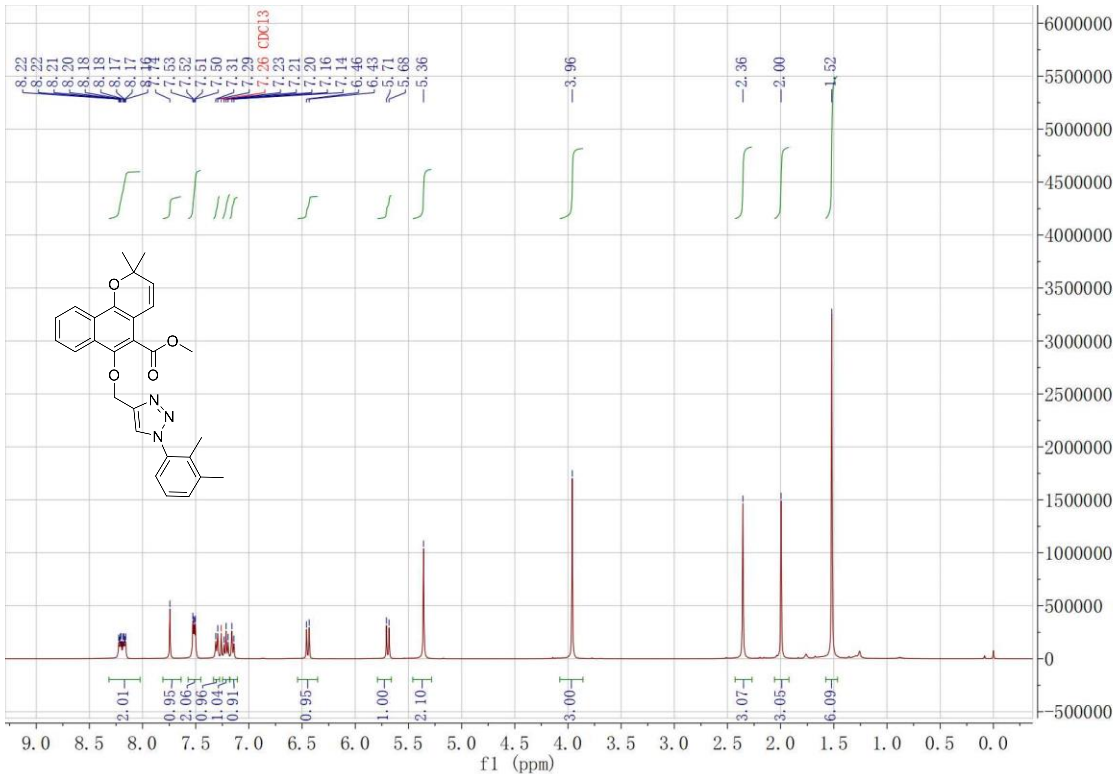

Figure 26-1. ^{1}H NMR spectrum of compound **30**

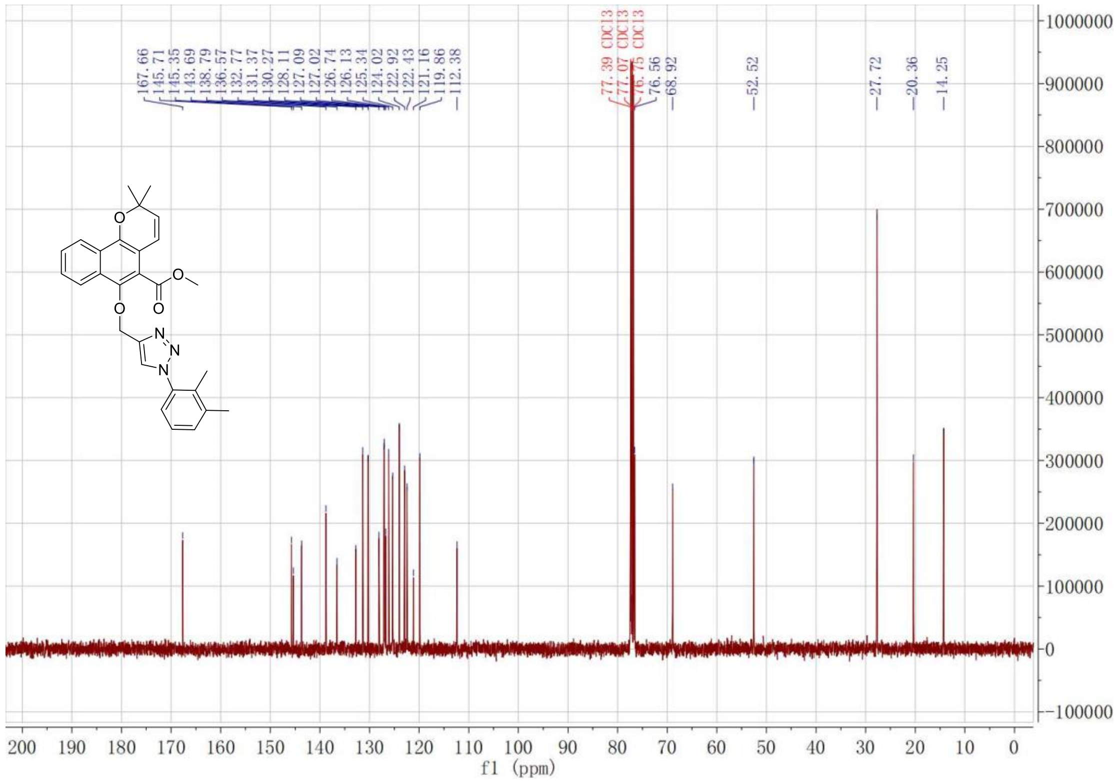

Figure 26-2. ^{13}C NMR spectrum of compound **30**

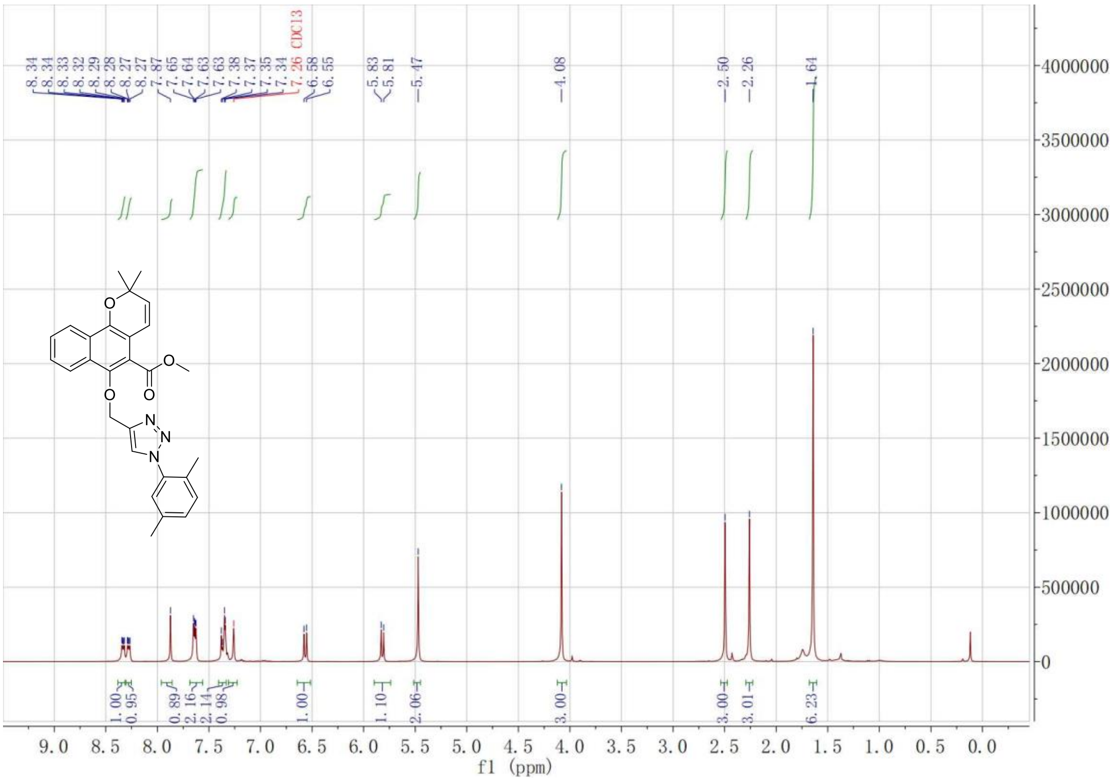

Figure 27-1. ^{1}H NMR spectrum of compound **31**

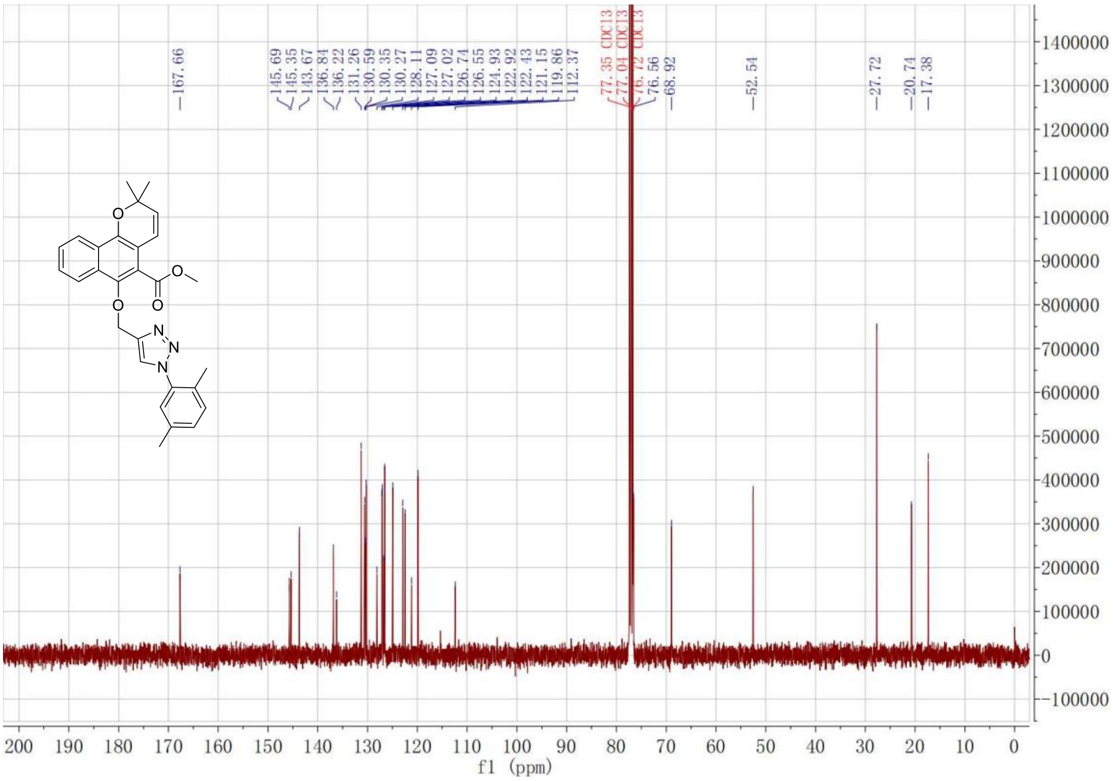

Figure 27-2. ^{13}C NMR spectrum of compound **31**

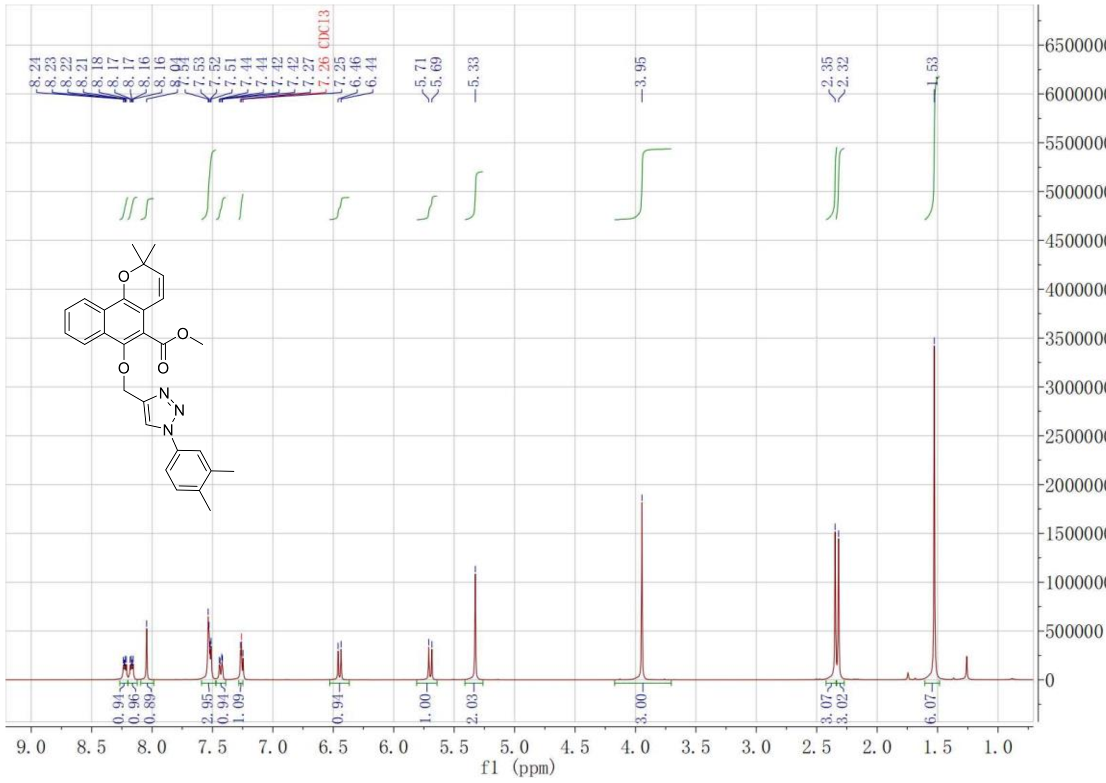

Figure 28-1. ^{1}H NMR spectrum of compound **32**

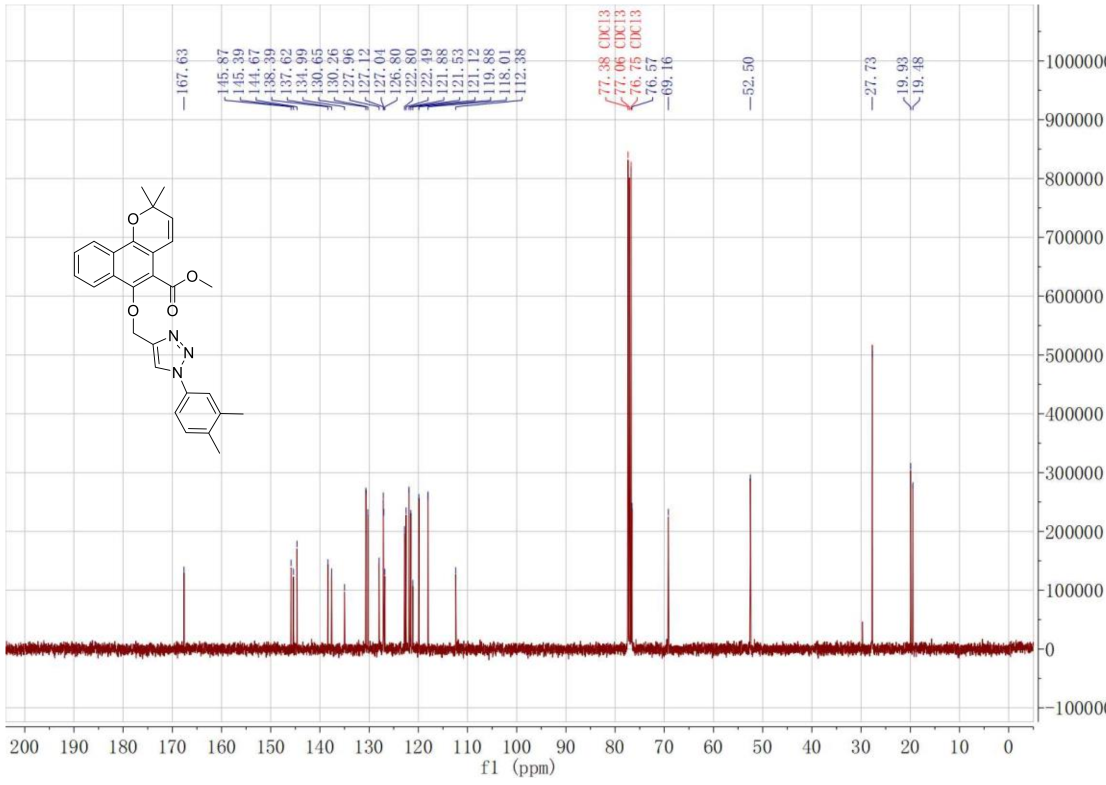

Figure 28-2. ^{13}C NMR spectrum of compound **32**

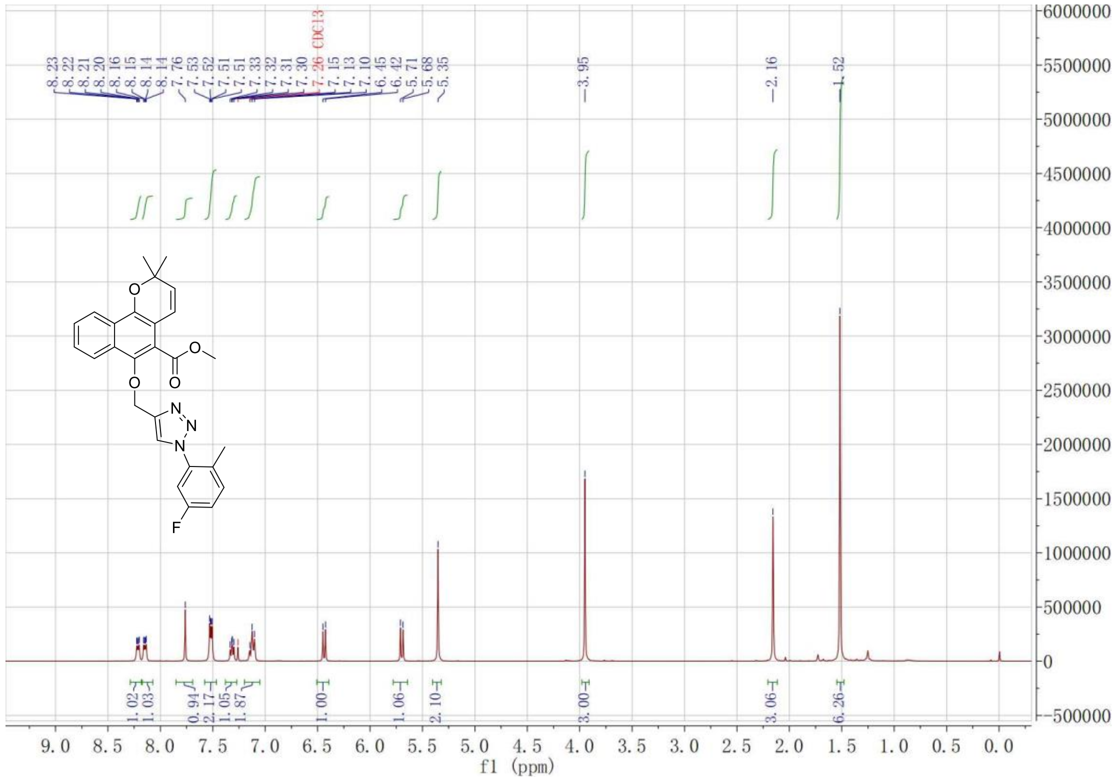

Figure 29-1. ^{1}H NMR spectrum of compound **33**

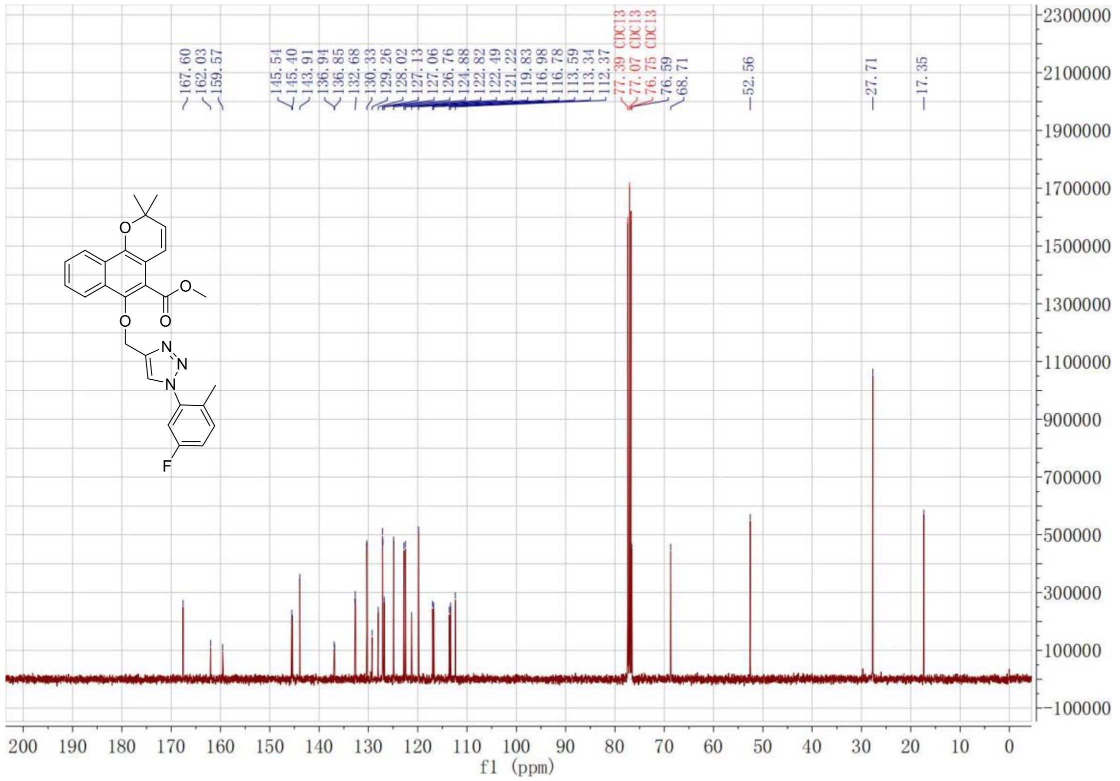

Figure 29-2. ^{13}C NMR spectrum of compound **33**

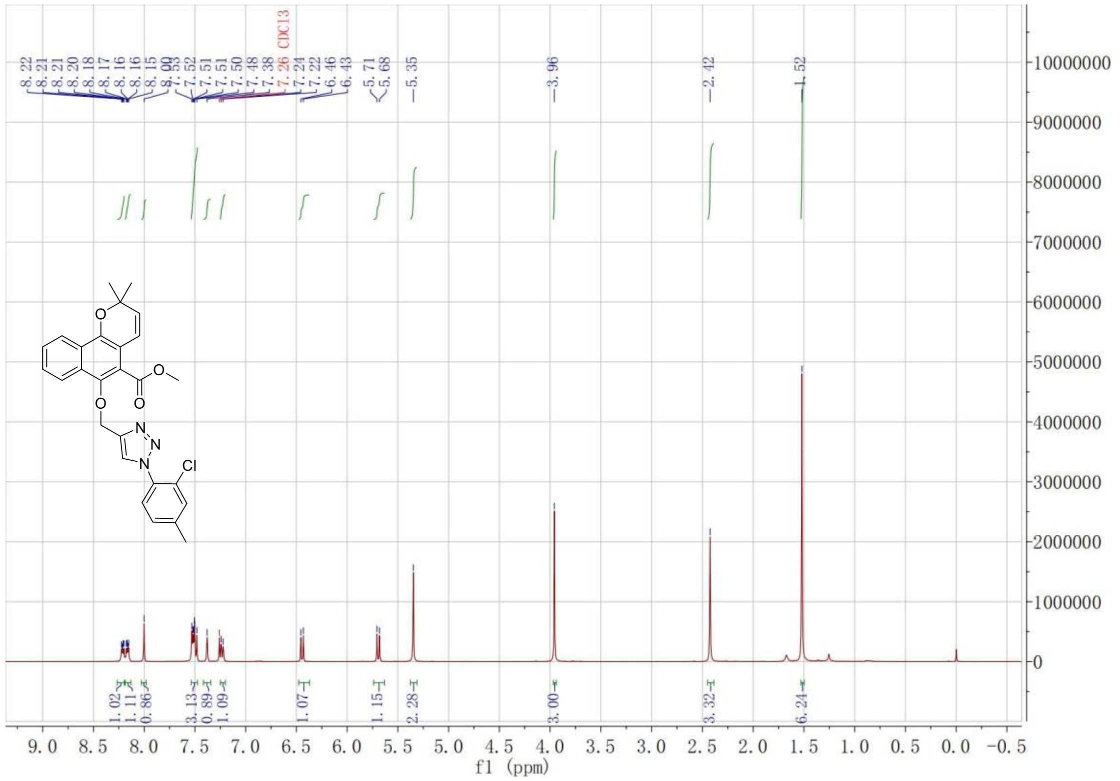

Figure 30-1. ^{1}H NMR spectrum of compound **34**

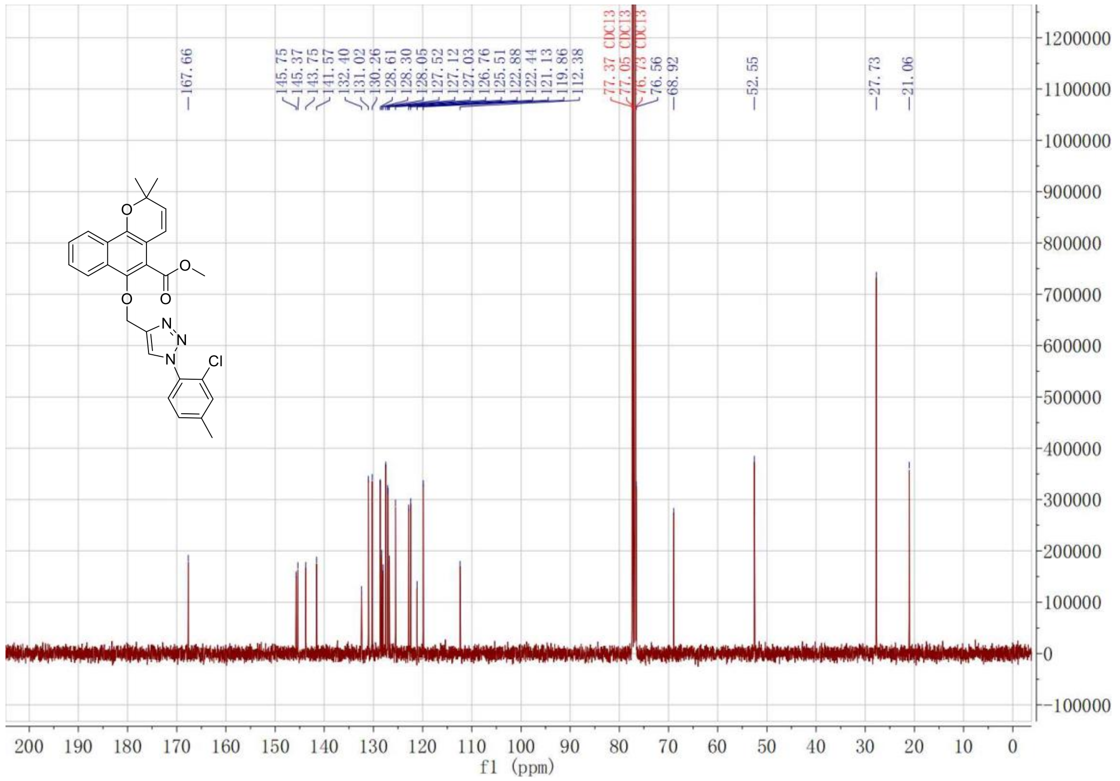

Figure 30-2. ^{13}C NMR spectrum of compound **34**

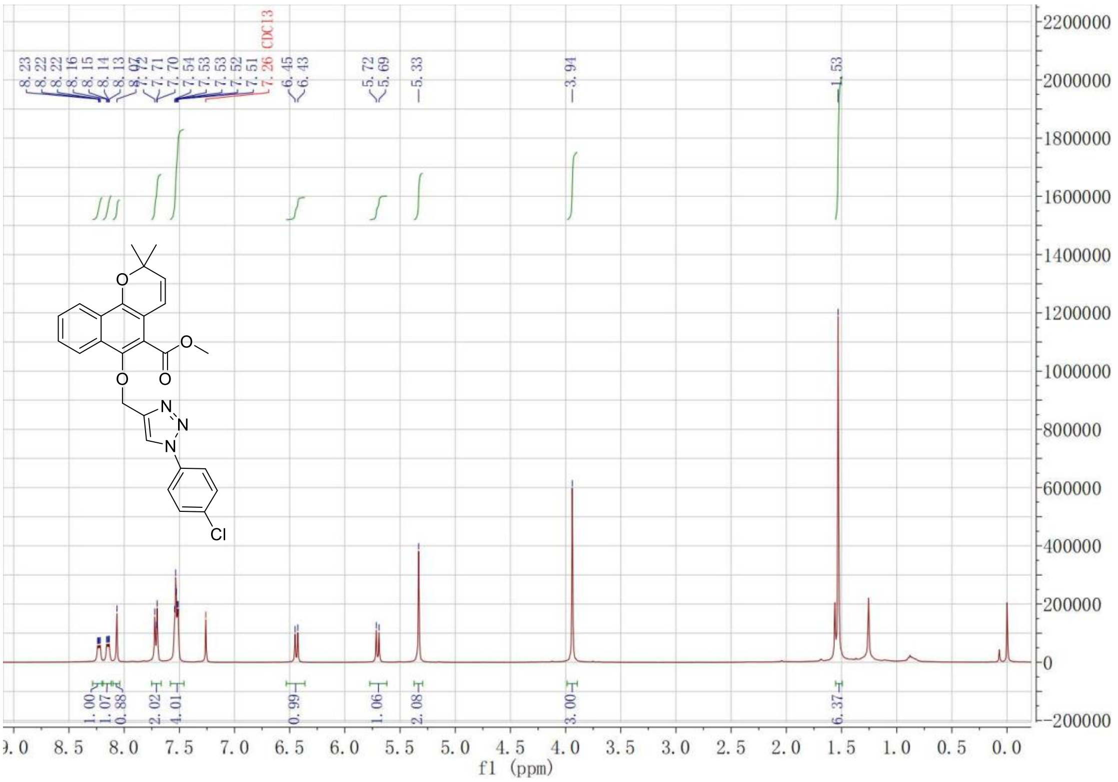

Figure 31-1. ^{1}H NMR spectrum of compound **35**

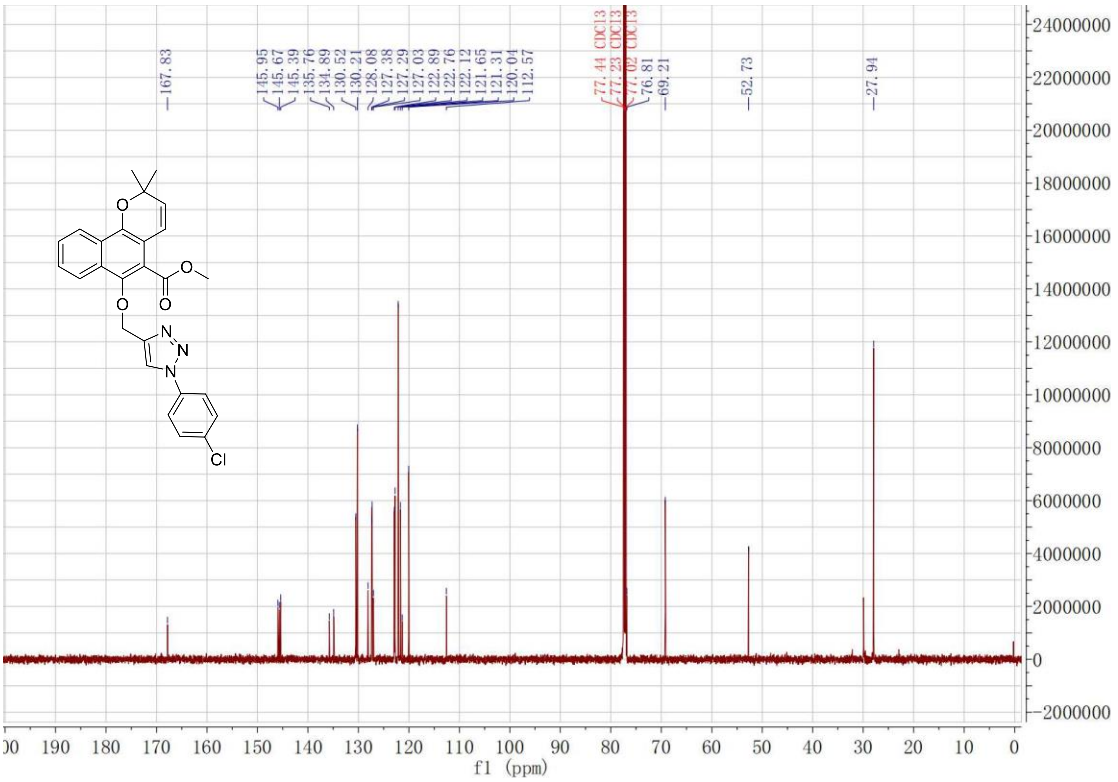

Figure 31-2. ^{13}C NMR spectrum of compound **35**

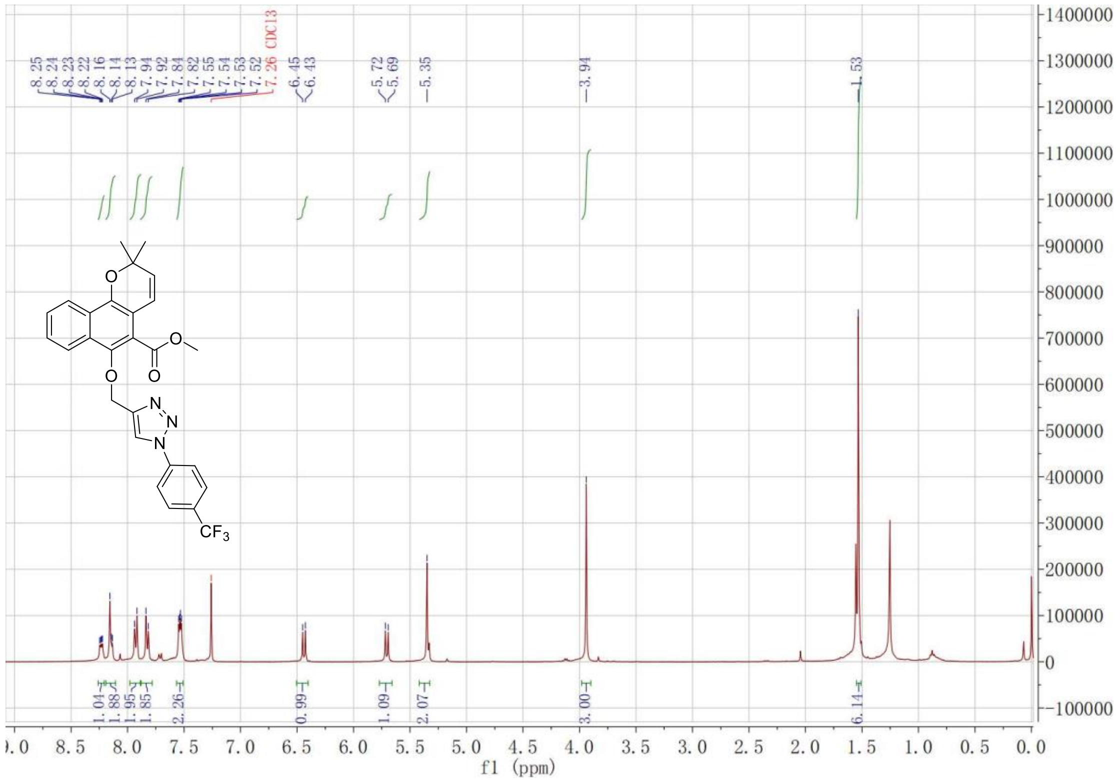

Figure 32-1. ^{1}H NMR spectrum of compound **36**

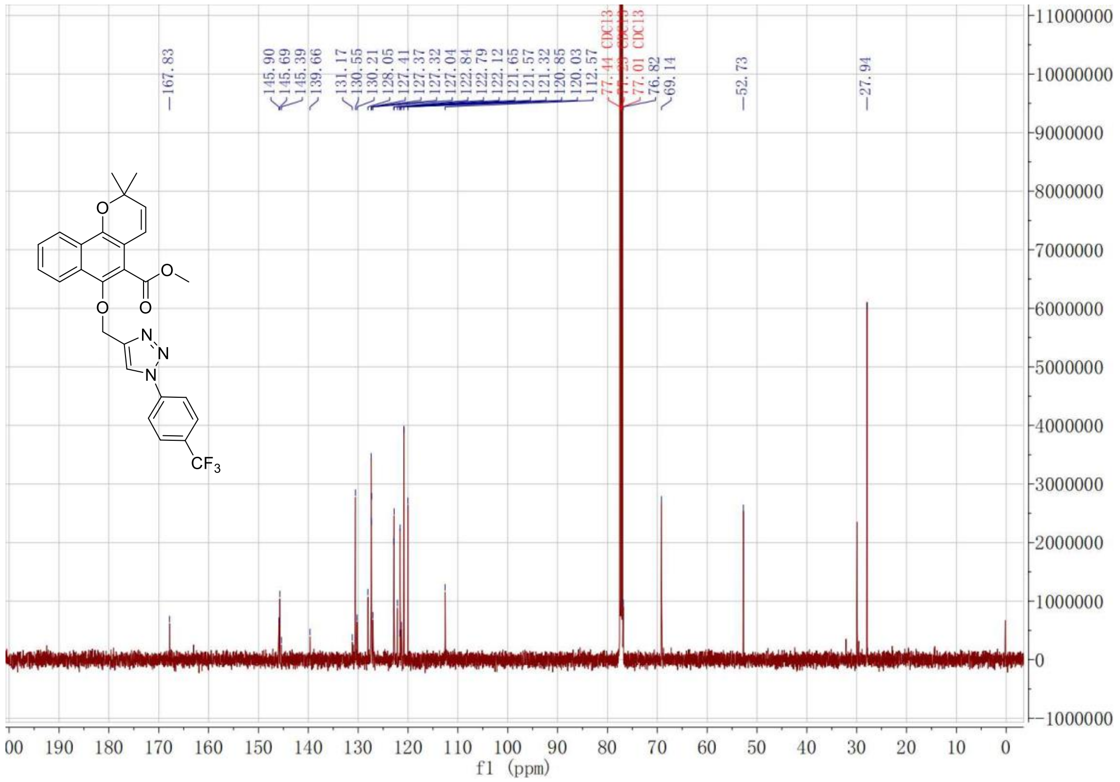

Figure 32-2. ^{13}C NMR spectrum of compound **36**

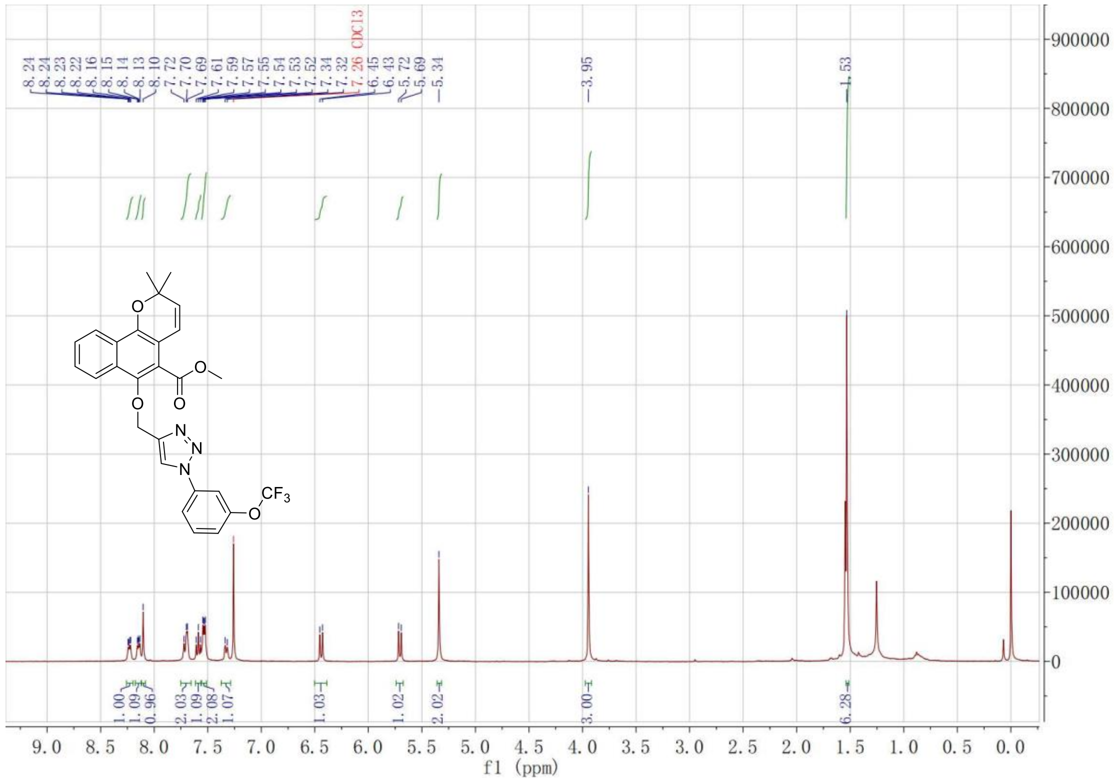

Figure 33-1. ^{1}H NMR spectrum of compound **37**

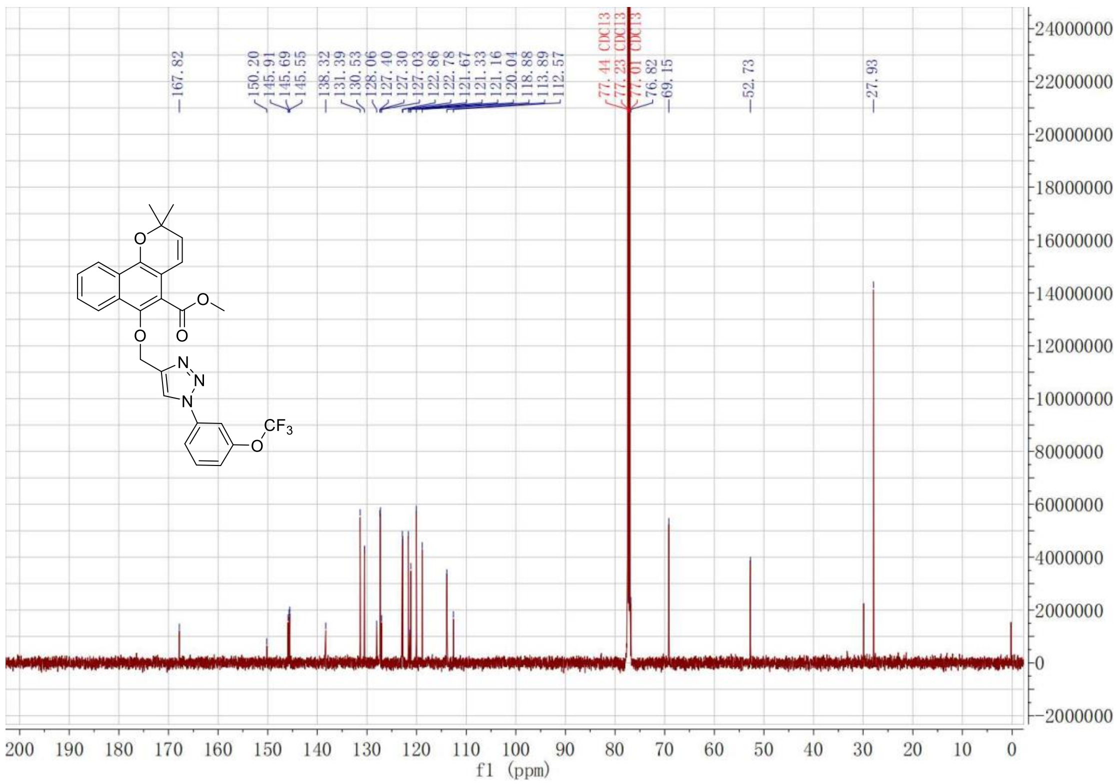

Figure 33-2. ^{13}C NMR spectrum of compound **37**

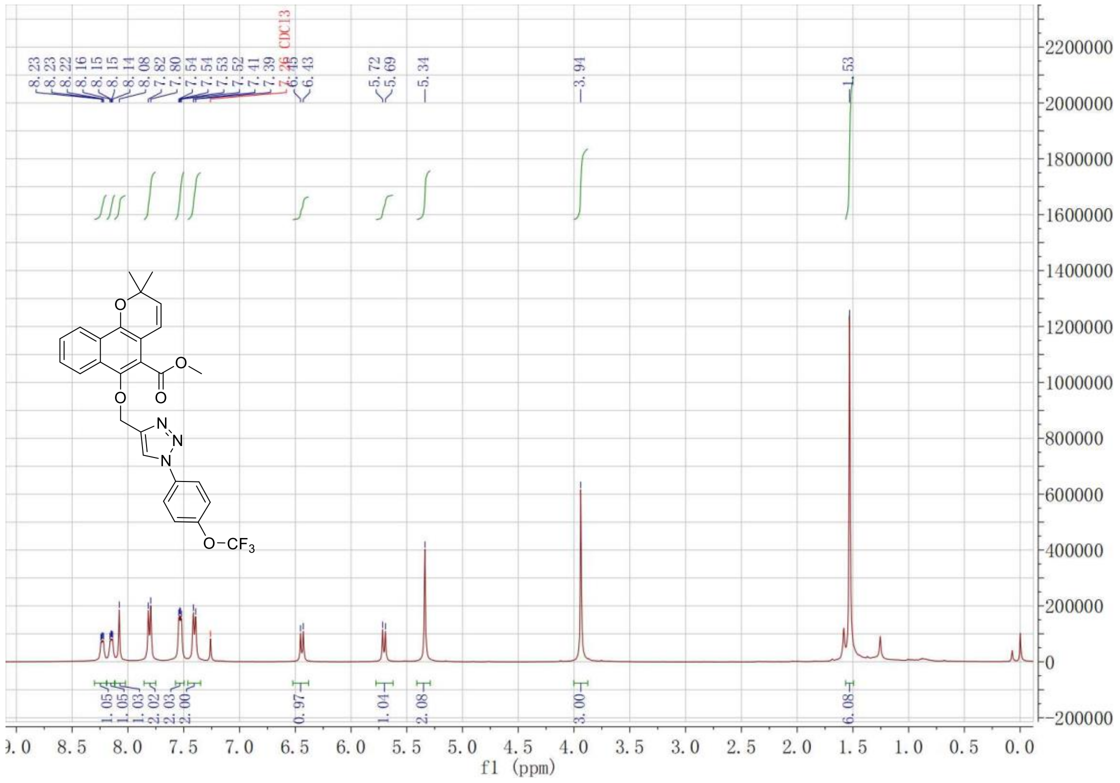

Figure 34-1. ^{1}H NMR spectrum of compound **38**

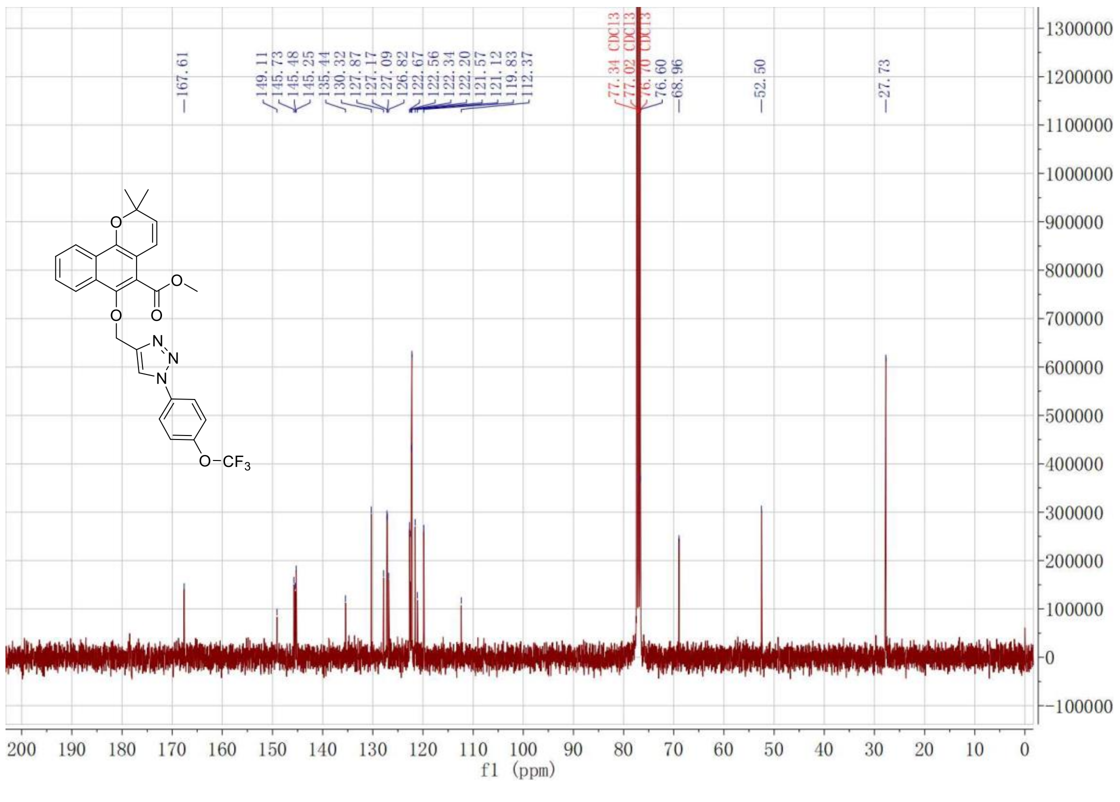

Figure 34-2. ^{13}C NMR spectrum of compound **38**

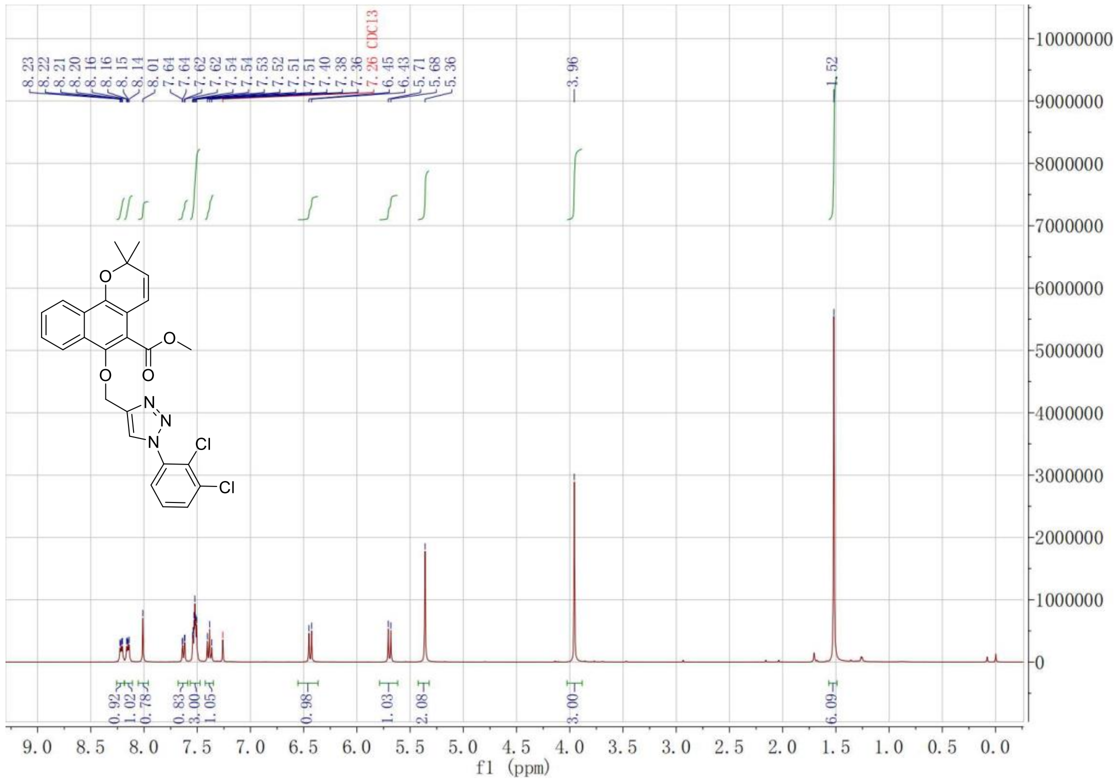

Figure 35-1. ^{1}H NMR spectrum of compound **39**

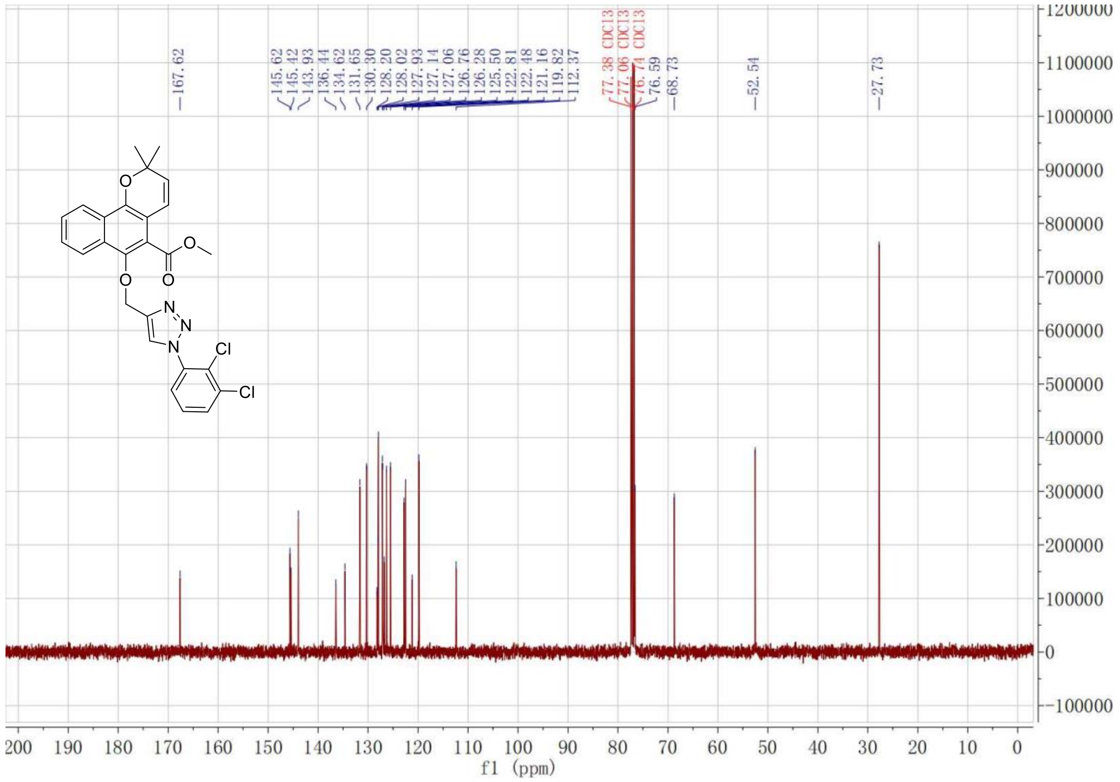

Figure 35-2. ^{13}C NMR spectrum of compound **39**

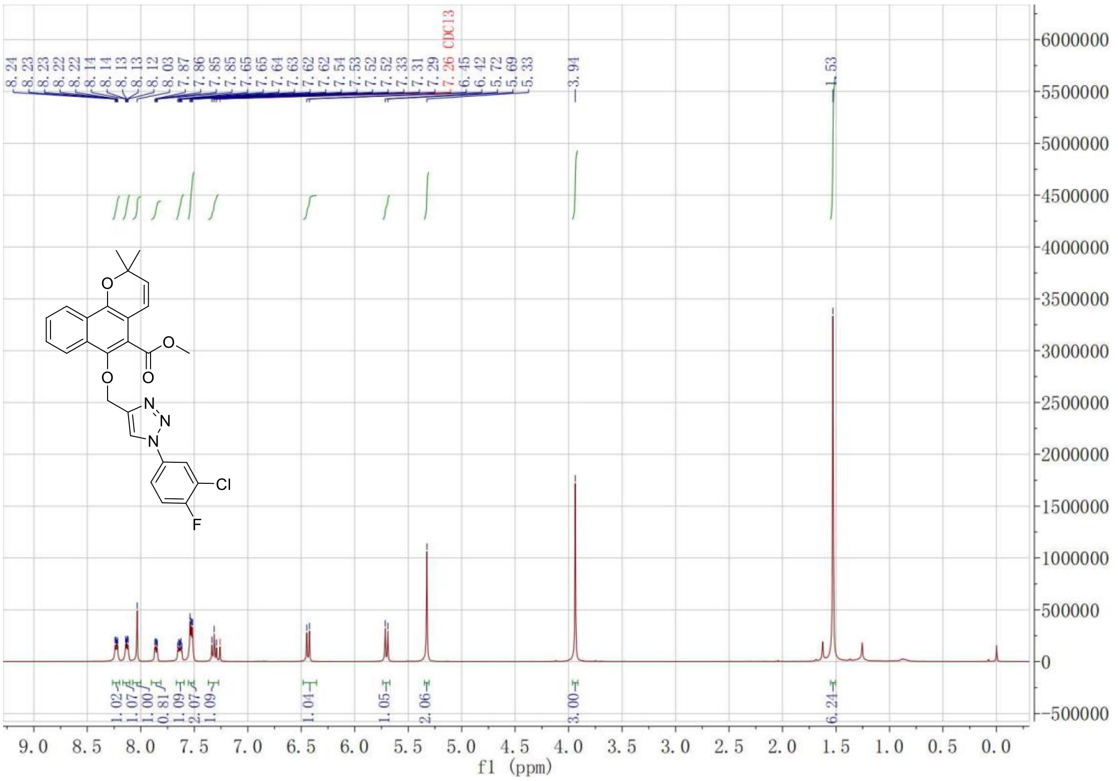

Figure 36-1. ^{1}H NMR spectrum of compound **40**

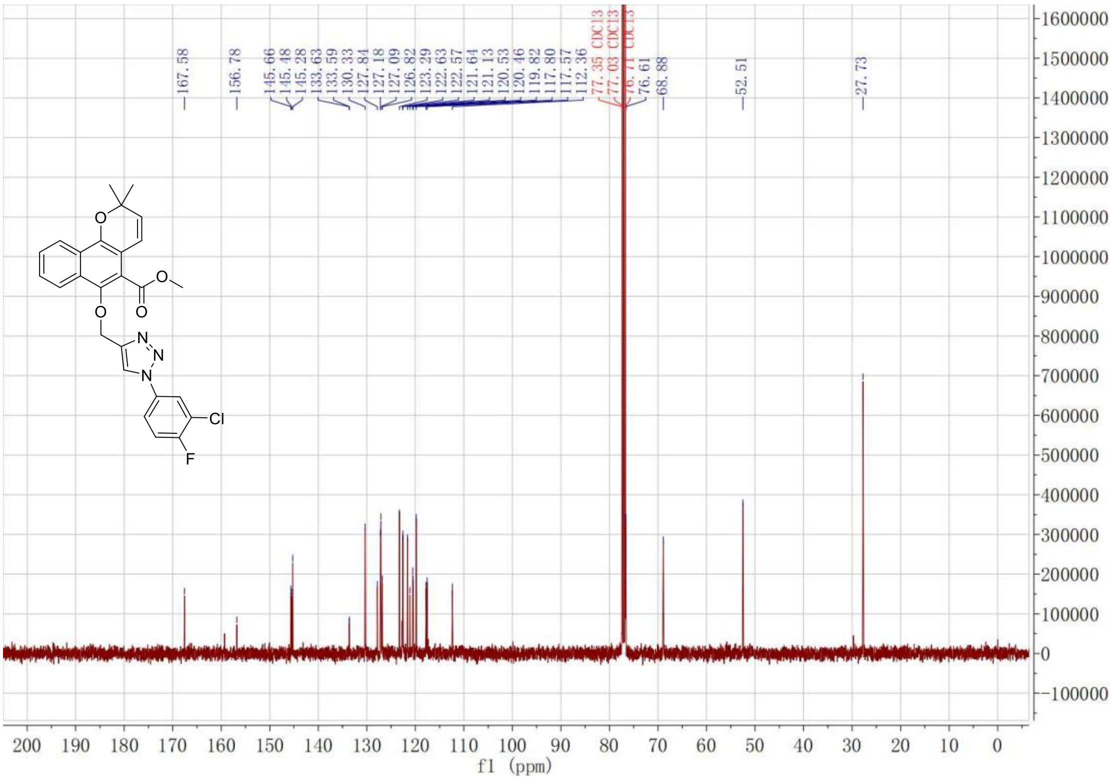

Figure 36-2. ^{13}C NMR spectrum of compound **40**

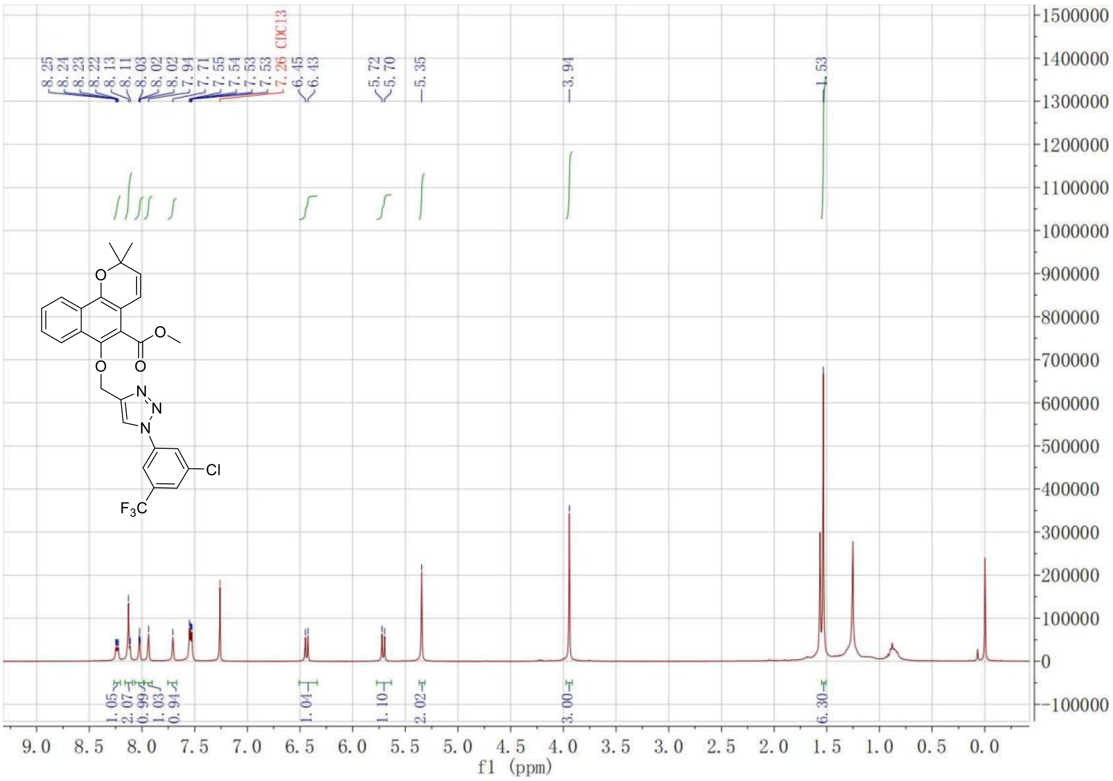

Figure 37-1. ^{1}H NMR spectrum of compound **41**

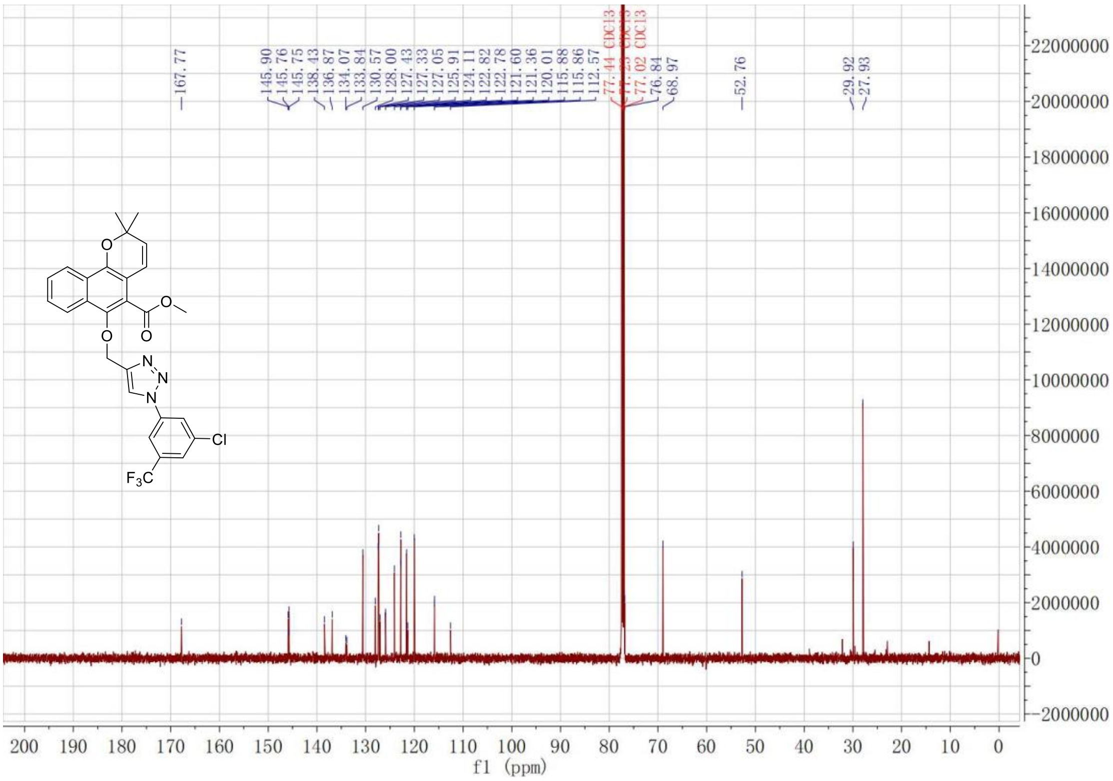

Figure 37-2. ^{13}C NMR spectrum of compound **41**

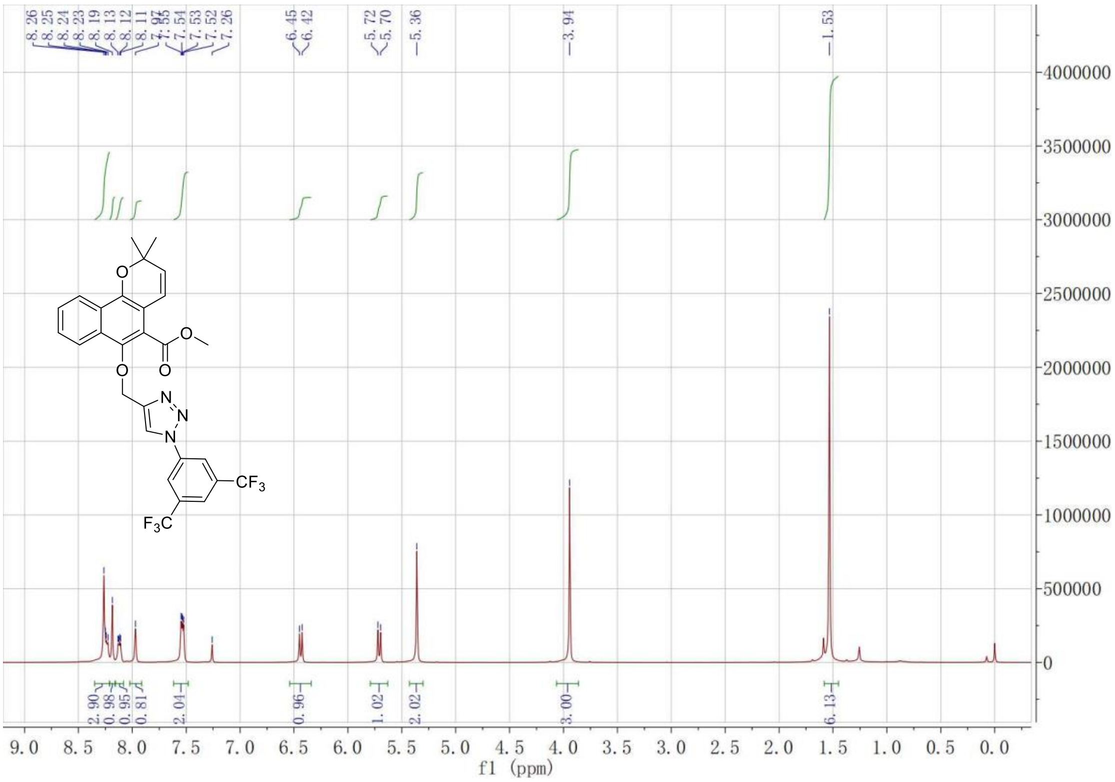

Figure 38-1. ^{1}H NMR spectrum of compound **42**

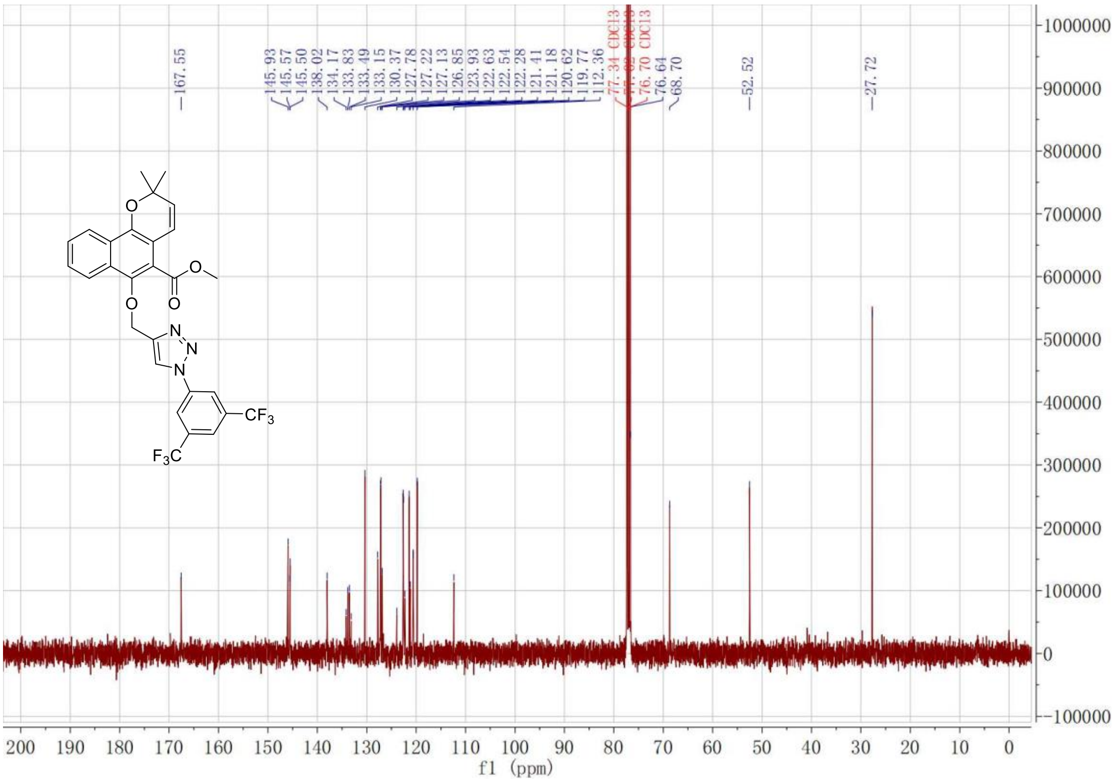

Figure 38-2. ^{13}C NMR spectrum of compound **42**

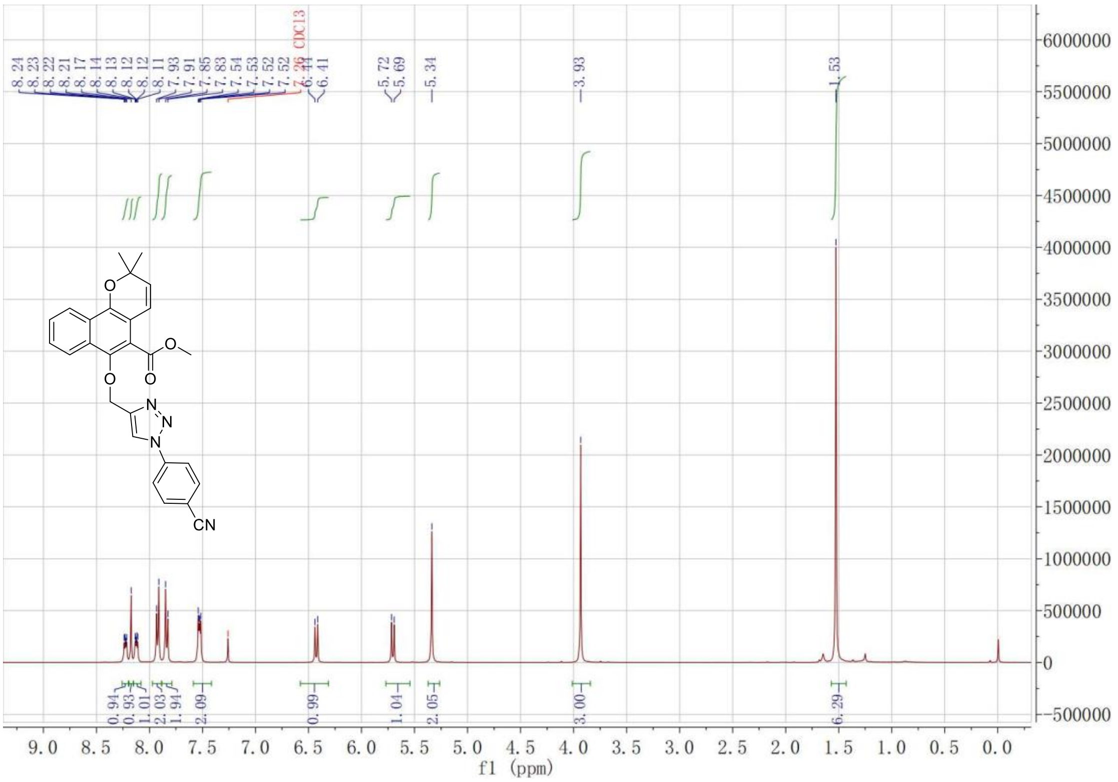

Figure 39-1. ^{1}H NMR spectrum of compound **43**

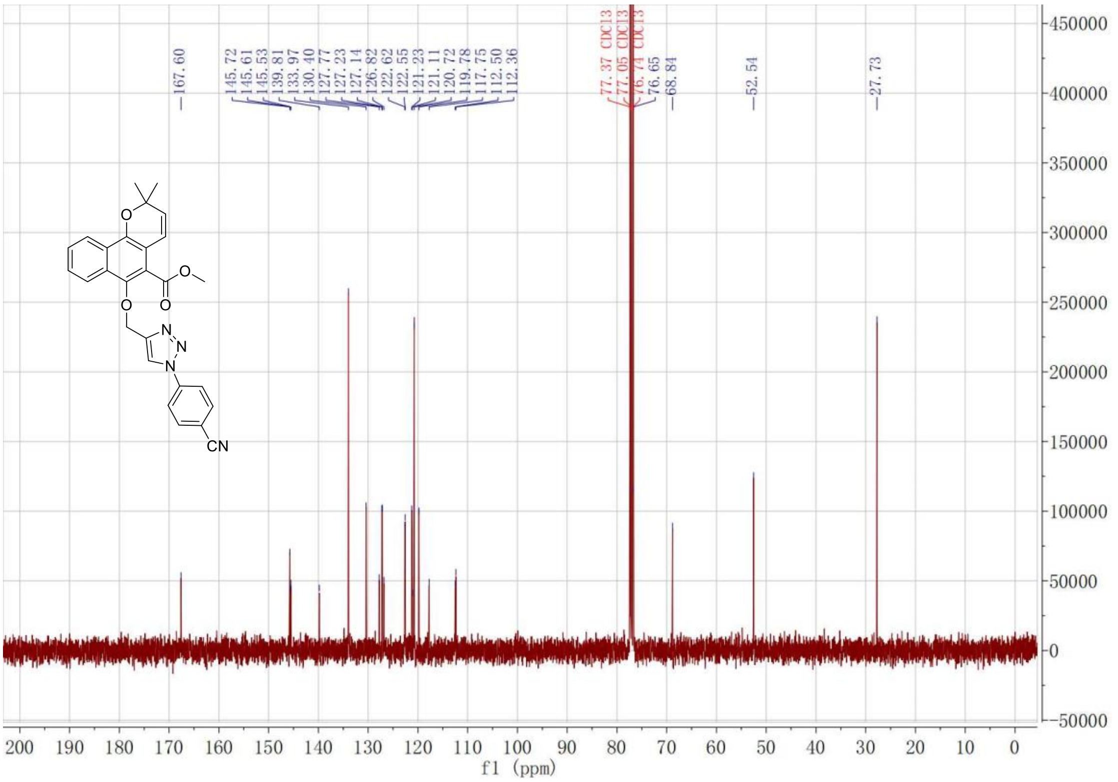

Figure 39-2. ^{13}C NMR spectrum of compound **43**

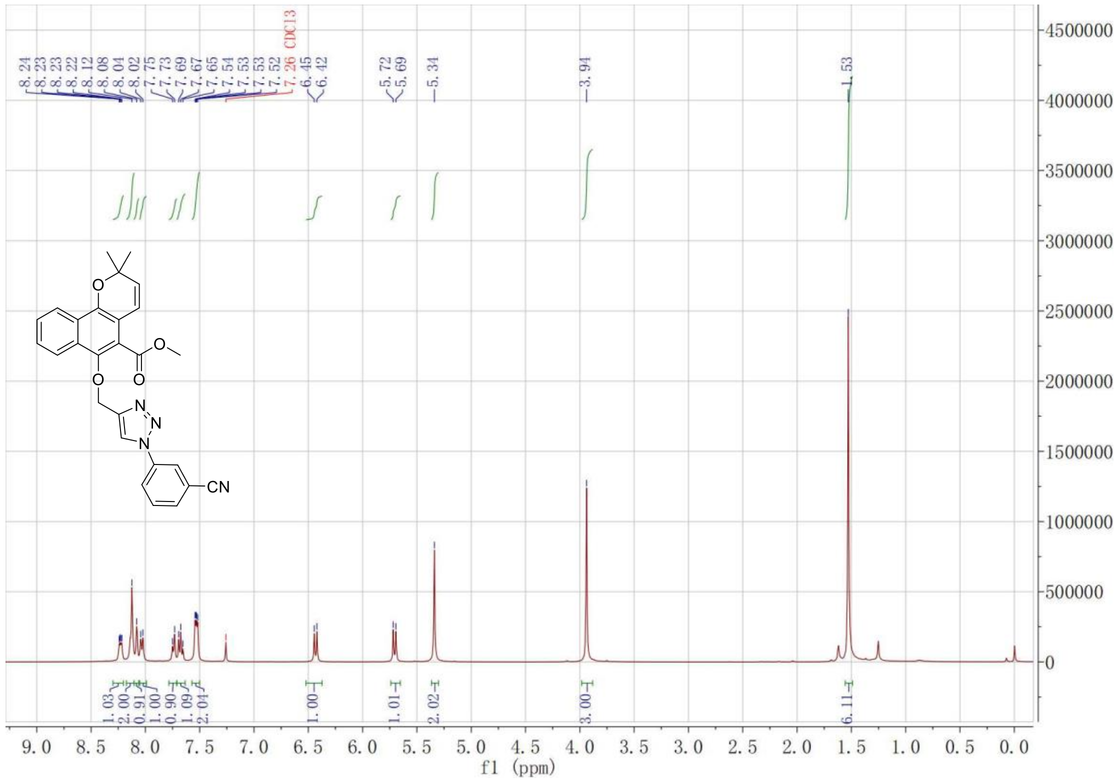

Figure 40-1. ^{1}H NMR spectrum of compound **44**

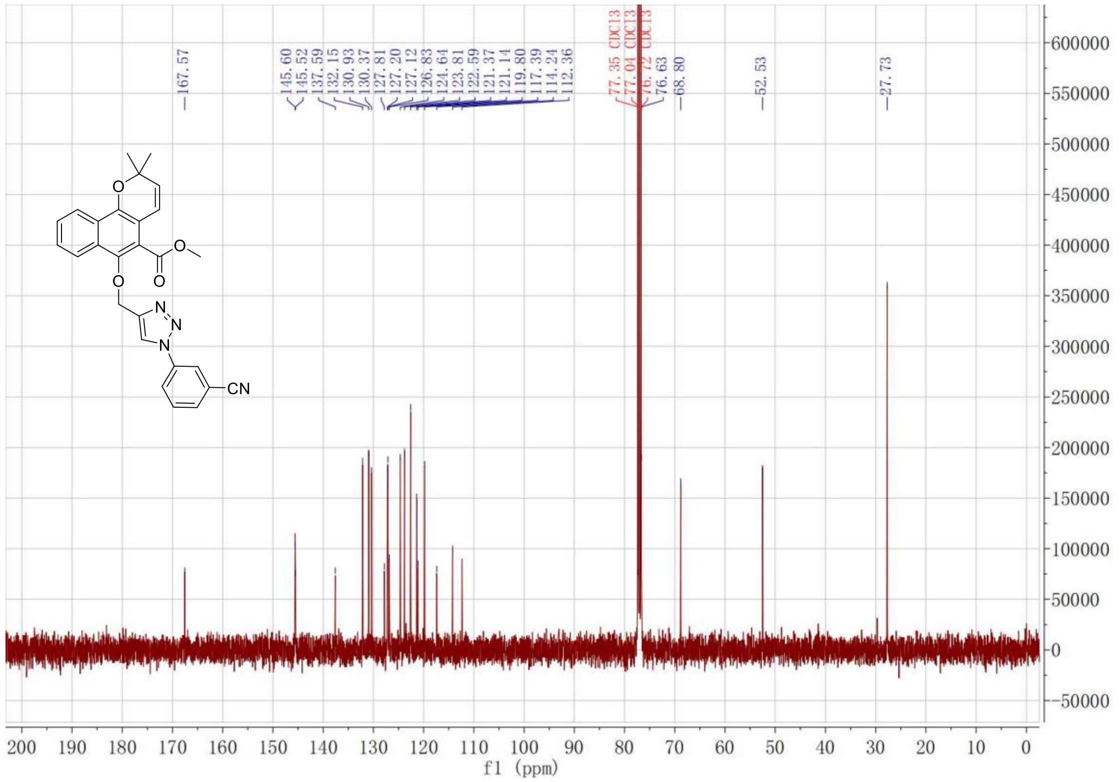

Figure 40-2. ^{13}C NMR spectrum of compound **44**
